# Stereoselective Synthesis of Nucleotide Analog Prodrugs (ProTides) via an Oxazaphospholidine Method

**DOI:** 10.1021/acs.joc.5c00240

**Published:** 2025-05-08

**Authors:** Monta Nakamura, Kiyoshi Kakuta, Kazuki Sato, Takeshi Wada

**Affiliations:** Department of Medicinal and Life Sciences, Faculty of Pharmaceutical Sciences, Tokyo University of Science, 2641 Yamazaki, Noda, Chiba 278-8510, Japan

## Abstract

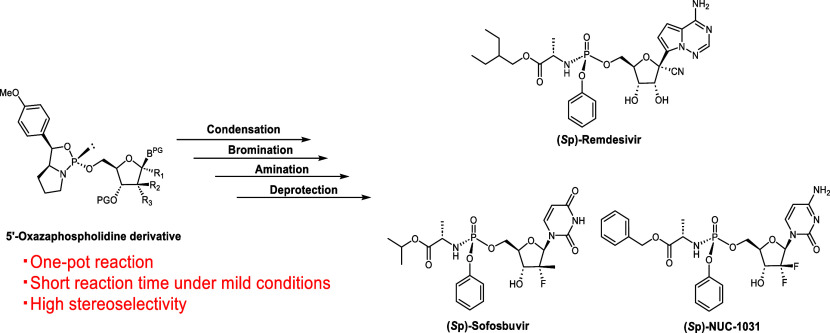

This paper describes the stereocontrolled synthesis of *O-*aryl phosphoramidate nucleotide prodrugs (ProTides), namely Remdesivir, Sofosbuvir, and NUC-1031, using an oxazaphospholidine method. The 5′-phosphoramidate derivatives were synthesized via a one-pot process involving the condensation of nucleoside 5′-oxazaphospholidine derivatives with phenol, bromination of the resulting phosphite using *N*-bromosuccinimide, and subsequent reaction with an amine. The target compounds were obtained with high stereoselectivity (dr >99:1). This one-pot strategy substantially reduced the reaction time from those of traditional phosphorylation methods using P(V) derivatives, providing an efficient and streamlined route to ProTides.

## Introduction

**Scheme 1:**
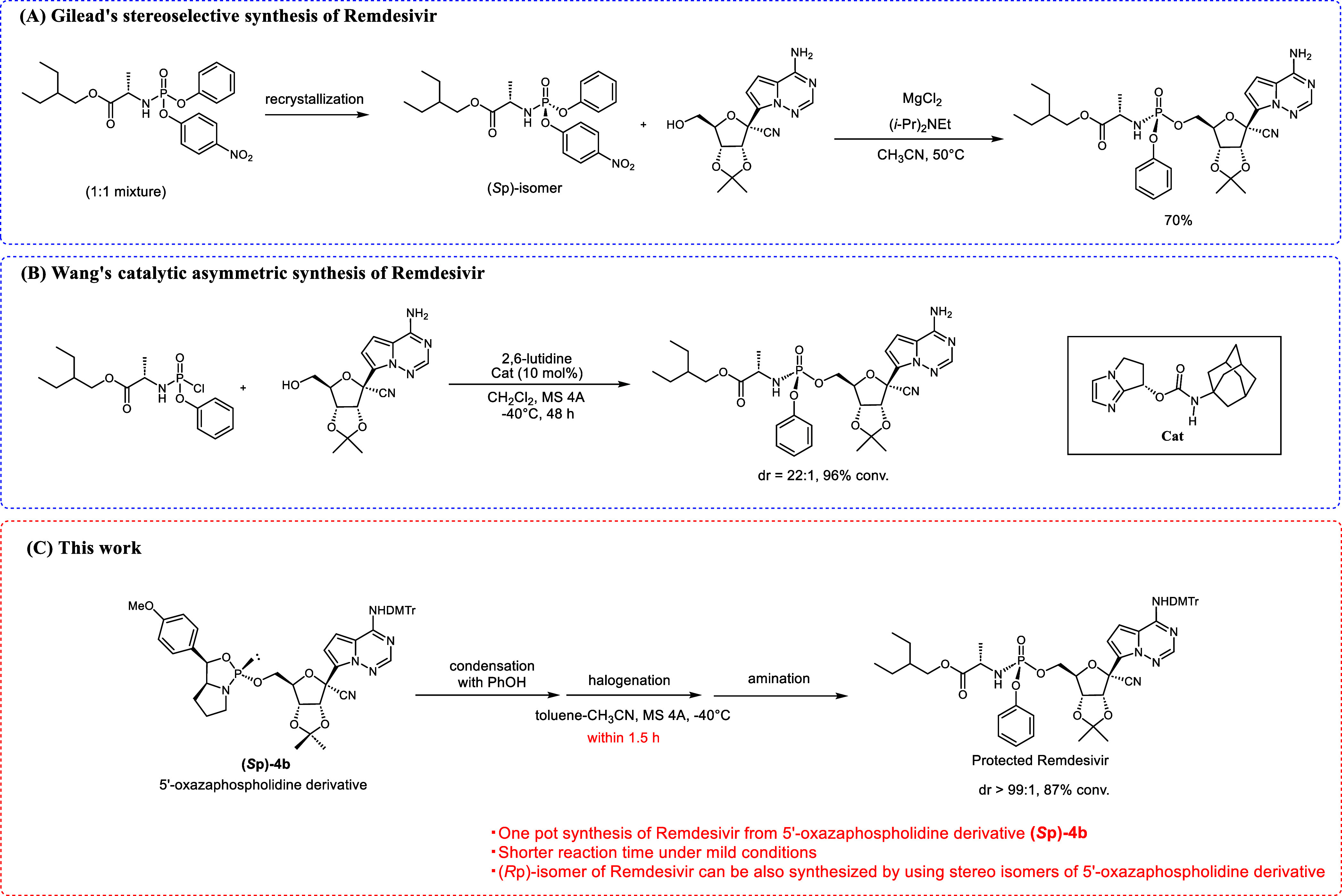
Stereoselective Synthesis of Remdesivir

Nucleoside analogs, widely used as antiviral or anticancer drugs, are taken up by cells and converted into 5′-mono, di-, and triphosphate forms (activated forms) by intracellular kinases.^1,2^ The activated forms are usually incorporated into DNA or RNA by polymerases. The structurally different nucleoside moiety terminates the chain elongation, eliciting antiviral or anticancer activity. The rate-determining step in this process is the 5′-monophosphorylation of nucleoside analogs by intracellular kinases. 5′-Nucleotide derivatives are inefficient owing to their low cell-membrane permeabilities. These limitations have been resolved with various prodrugs,^3^ including ProTides, 5′-phosphoramidate nucleotide prodrugs bearing an *O-*aryl group and an amino acid ester. Developed by McGuigan, the ProTide technology enhances the cell membrane permeabilities of parent nucleotides based on the hydrophobicity of a phosphoramidate moiety, enabling efficient drug delivery.^4,5^ ProTides are rapidly converted into 5′-monophosphate forms by intracellular enzymes such as CES/CatA and Hint1.^6^ Recognizing these advantageous characteristics, the Food and Drug Administration (FDA) has approved ProTides such as Remdesivir **(*****S*****p)-1** for COVID-19 treatment and Sofosbuvir **(*****S*****p)-2** for hepatitis C treatment.^7,8^ The Gemcitabine prodrug NUC-1031 **3** has also been investigated in various clinical trials, but was unfortunately discontinued after the phase 3 trials yielded unsatisfactory results.^9^ Nevertheless, the ProTide technology has been applied to many nucleotide analogs and continues to be rigorously investigated for its promising advantages. Although ProTides improve the properties of parent nucleotides, they exist as diastereomers owing to their chiral phosphorus center. Remdesivir and Sofosbuvir are *S*p isomers with higher pharmacological activities than their *R*p counterparts.^7,8^ In typical ProTides synthesis, a phosphorylating reagent is reacted with the 5′-hydroxy group of a nucleoside analog, yielding a diastereomeric mixture. The desired stereoisomer of ProTides or the phosphorylating reagent is often separated through chiral high-performance liquid chromatography (HPLC)^10^ or recrystallization.^11^ For example, Gilead’s synthesis isolates the stereopure phosphorylating reagent through recrystallization^11^ (Scheme 1A). However, the atomic economy of this method is low because the undesired stereoisomer is discarded. To resolve this problem, Wang and coworkers developed a unique chiral bicyclic imidazole catalyst for the synthesis of a protected Remdesivir with high stereoselectivity^12^ (Scheme 1B). More recently, stereoselective synthesis of ProTides using a Cu complex has been reported.^13^ Although these methods produce ProTides with high stereopurity, the 5′-phosphorylating step generally requires prolonged reaction times or harsh conditions, such as high temperatures, because P(V) phosphorylating reagents are less reactive. Thus, developing a new stereoselective synthesis method remains a substantial challenge. Herein, we aim to develop a novel stereoselective synthesis method for ProTides using highly reactive P(III) compounds, namely, 5′-oxazaphospholidine derivatives (Scheme 1C). In our laboratory, an oxazaphospholidine method was developed for the stereoselective synthesis of *P*-modified oligonucleotides such as phosphorothioates^14^ and boranophosphates.^15^ An oxazaphospholidine is a cyclic phosphoramidite derivative containing a chiral auxiliary. During the stereoselective synthesis of *P*-modified oligonucleotides, a nucleoside 3′-oxazaphospholidine derivative is condensed with a 5′-hydroxy group of nucleosides in the presence of *N*-(cyanomethyl) pyrrolidinium triflate (CMPT), a non-nucleophilic acidic activator, affording stereopure phosphite triester.^14−16^

A chiral auxiliary containing phenyl and methyl groups at the 5-position of the oxazaphospholidine ring yields a phosphite triester intermediate, which is stereoselectively converted to the corresponding *H*-phosphonate diester under acidic conditions, liberating the chiral auxiliary as a stabilized tertiary carbocation.^16^ Subsequent conversion of the *H*-phosphonate diester affords *P*-modified oligonucleotides. Stereopure phosphoramidates can also be synthesized via the Atherton–Todd reaction.^16^ The same strategy can be adopted for a chiral auxiliary with a 4-methoxyphenyl group at the 5-position, whereby the chiral auxiliary is removed by forming a secondary benzyl cation stabilized by the electron-donating effect of the methoxyphenyl group.^15^ We envision that ProTides can be stereoselectively synthesized from nucleoside 5′-oxazaphospholidine derivatives via an *H*-phosphonate diester intermediate.

## Results and Discussion

**Scheme 2:**
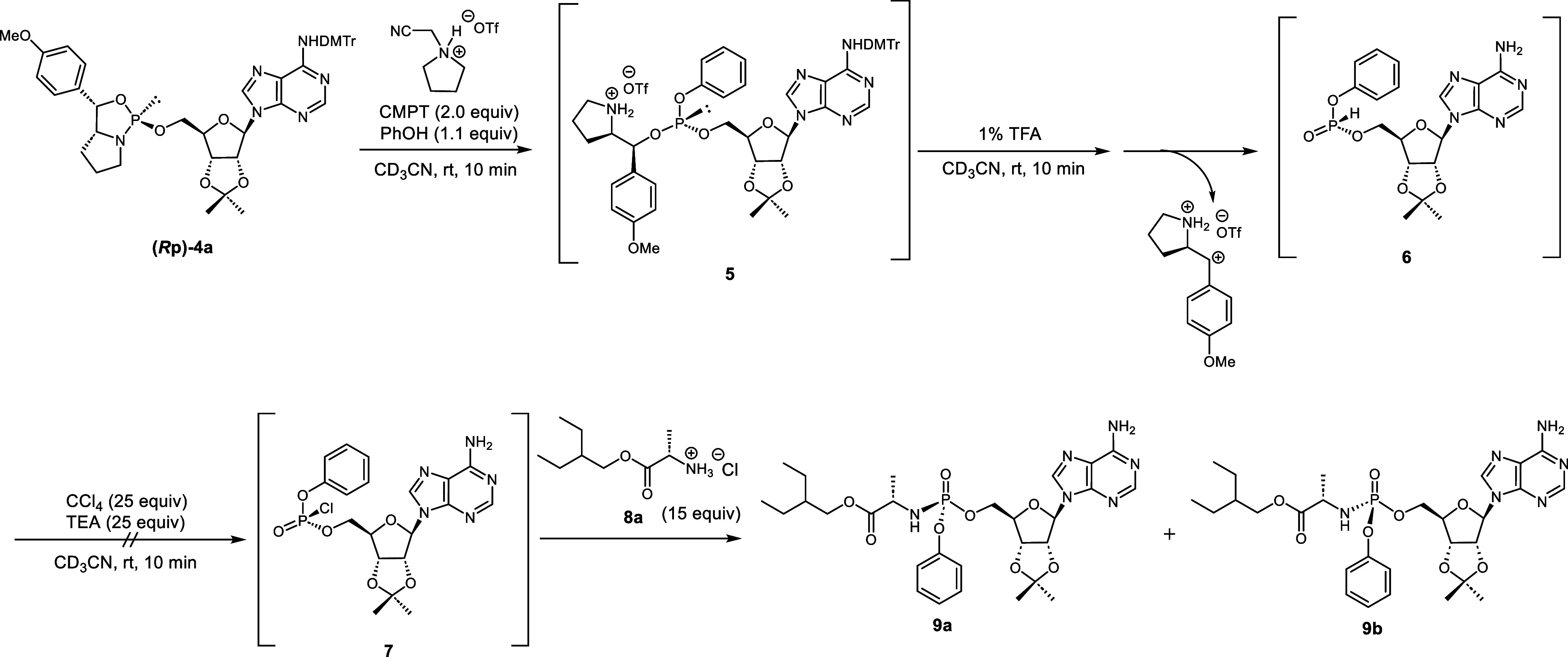
Synthesis of the Model Compound via an *H*-Phosphonate

To test this concept, we synthesized 5′-oxazaphospholidine derivatives containing a 4-methoxyphenyl group at the 5-position of oxazaphospholidine as a model compound. Phosphitylation of the 5′-hydroxy group of an adenosine derivative, whose nucleobase amino group is protected by a DMTr group, yielded the corresponding 5′-oxazaphospholidine derivative **(*****R*****p)-4a** with moderate yield (35%) and high stereoselectivity (trans:cis >99:1) (Supporting Information). Because oxazaphospholidine derivatives and other P(III) compounds are prone to hydrolysis and oxidation, **(*****R*****p)-4a** was converted to the *O-*aryl phosphoramidate through a one-pot process without purification or isolation of the intermediates (phosphite triester **5** and *H-*phosphonate **6**, Scheme 2). The reactions were monitored by ^31^P NMR. When the 5′-oxazaphospholidine derivative **(*****R*****p)-4a** was condensed with phenol (PhOH) in the presence of CMPT at room temperature for 10 min, the ^31^P NMR signal of the oxazaphospholidine derivative (δ_P_ = 153) disappeared and a new signal (δ_P_ = 132) corresponding to a phosphite triester **5** appeared. Next, the chiral auxiliary of the phosphite triester was removed by treatment with 1% trifluoroacetic acid (TFA), followed by the Atherton–Todd reaction using CCl_4_, trimethylamine (TEA), and amino acid ester **8a**. However, this synthesis yielded the *H*-phosphonate derivative **6** rather than the desired *O*-aryl phosphoramidate **9**, indicating that the Atherton–Todd reaction was hindered by salt formation caused by excess TFA and TEA (Scheme 2).

**Scheme 3:**
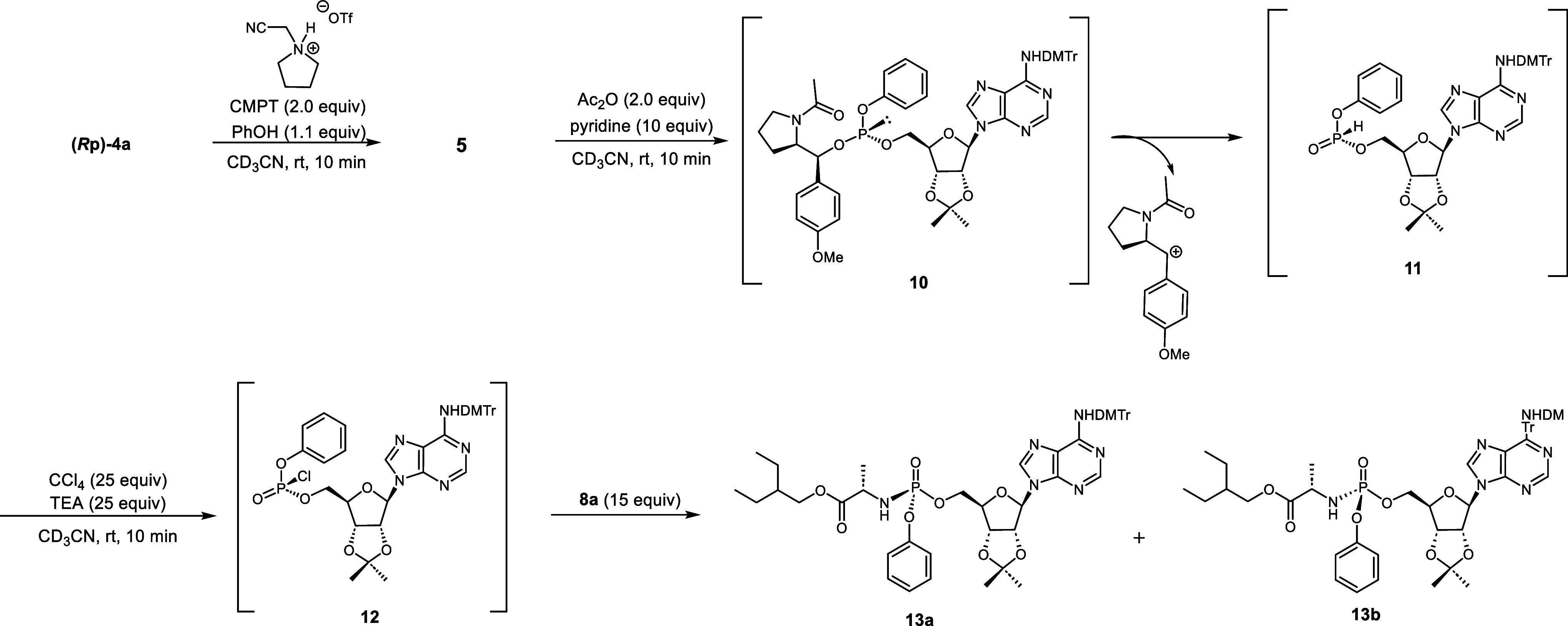
Synthesis of the Model Compound via Acylation of a Phosphite Triester

We expected that the *N*-acylated chiral auxiliary could be easily removed as a monocation species, affording the stereopure *H-*phosphonate diester **11** under mild conditions (Scheme 3). To this end, we added acetic anhydride and pyridine to the reaction mixture containing the phosphite triester intermediate. The ^31^P NMR signal of the phosphite triester completely disappeared within 10 min and a new signal (δ_P_ = 8.9) corresponding to the desired *H-*phosphonate diester appeared, confirming protonation of the phosphite triester and removal of the chiral auxiliary. After adding CCl_4_, TEA, and the amino acid ester **8a** to the reaction mixture, the *H*-phosphonate diester was converted to its phosphoramidate counterpart. The formation of compound **13** was confirmed by ^31^P NMR. Furthermore, after ^31^P NMR spectroscopy, reverse-phase (RP)-HPLC, and electrospray ionization mass spectrometry, the product was confirmed as a stereomixture (dr = 52:48) (Figure S6). The poor stereoselectivity was attributed to nucleophilic attack of pyridine on the phosphorochloridate intermediate **12**. Acylation failed when low nucleophilic bases such as 2,6-lutidine and quinoline were employed during the acylation step.

To bypass the acylation step, we halogenated the phosphite triester **5** through direct formation of a phosphorohalogenate derivative. Among various halogenation conditions, employing *N*-bromosuccinimide (NBS) as the halogenation reagent and adding amino acid ester **8b** best yielded the desired product **13a**. The plausible mechanism is bromophosphonium **11** formation from phosphite triester via the reaction with NBS. Subsequently, the chiral auxiliary is eliminated as a dication, forming the phosphorobromidate intermediate **15**. The nucleophilic attack of amine **8b** on compound **15** produces the final product. Although the one-pot reaction yielded the product at 0 °C, the NMR yield and stereoselectivity were low (28% NMR yield, dr = 70:30). Therefore, we improved the yield by optimizing the reaction temperature (Table 1, Entries 1–3). Lowering the reaction temperature to −40 °C suppressed the main side reactions—hydrolysis and oxidation of the 5′-oxazaphospholidine derivative—improving the stereoselectivity (dr >99:1) but leaving a precipitate in the reaction mixture, likely resulting from the low solubility of **(*****R*****p)-4a**. A mixed solvent of CH_3_CN and toluene effectively dissolved the reactant, yielding a good NMR yield (78%) and excellent stereoselectivity as confirmed by ^31^P NMR and RP-HPLC (Table 1, Entry 4) (dr >99:1, Figure S7). The product **13a** was isolated in 37% yield. In addition, we synthesized the (*S*p)-5′-oxazaphospholidine derivative (46%, trans:cis >99:1) and prepared the corresponding phosphoramidate stereoisomer under the reaction conditions of Entry 4 in Table 1. The stereopurity of the obtained phosphoramidate **13b** was then determined (Supporting Information). The assigned stereochemistries of both obtained phosphoramidates are discussed later. Judging from these results, the method effectively synthesizes both *R*p and *S*p stereoisomers.

**Table 1:**
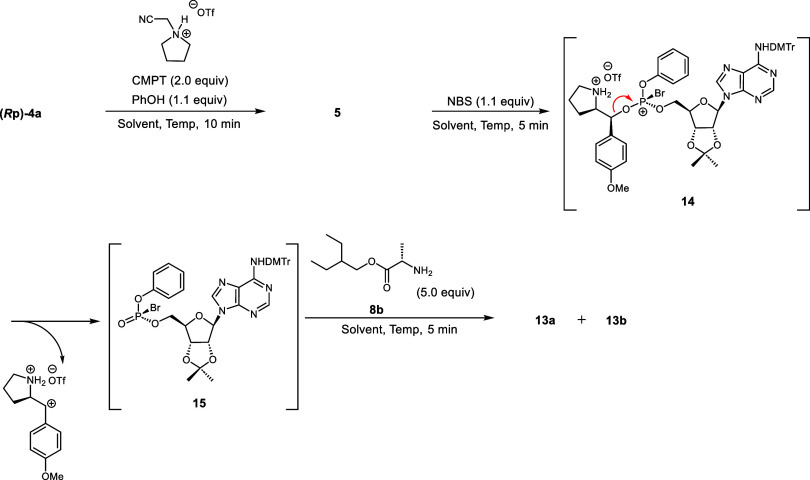
Investigation of Halogenation Reaction Conditions

Entry^a^	Solvent	Temp (°C)	^31^P NMR yield of **13** (%)	Isolated yield of **13** (%)	dr^b^
1	CD_3_CN	0	28	―	70:30
2	CD_3_CN	–20	36	16	88:12
3	CD_3_CN	–40	53	25	>99:1
4	CH_3_CN-toluene^c^	–40	78	39	>99:1

a Entries 1–3 and Entry 4 were conducted on 0.03 and 0.18 mmol scales, respectively.

b Entry 1: Determined from the ^31^P NMR spectrum of crude mixture. Entries 2 and 3: Determined from the ^31^P NMR spectra after purification. Entry 4 was determined from RP-HPLC after deprotection of the DMTr group of the model compound.

c All reagents were added as CH_3_CN solution.

Encouraged by the successful stereoselective synthesis of the model compounds, we synthesized Remdesivir **(*****S*****p)-1** and its stereoisomer **(*****R*****p)-1**. Similarly to **(*****R*****p)-4a**, we also synthesized the 5′-oxazaphospholidine derivative **(*****R*****p)-4b** (46%, trans:cis >99:1) (Supporting Information). The compound **(*****R*****p)-4b** was then condensed with PhOH, followed by bromination and subsequent reaction with compound **8b** to afford **16b** (Table 2, Entry 1). However, the ^31^P NMR spectrum of the crude mixture revealed a low NMR yield of **16b (**only 34%) and a dominance of the byproduct (δ_P_ = 22.9). Although we could not isolate and analyze the unstable byproduct, we confirmed that the byproduct was formed even in the reaction of the 5′-oxazaphospholidine derivative with CMPT and NBS in the absence of PhOH

**Table 2:**
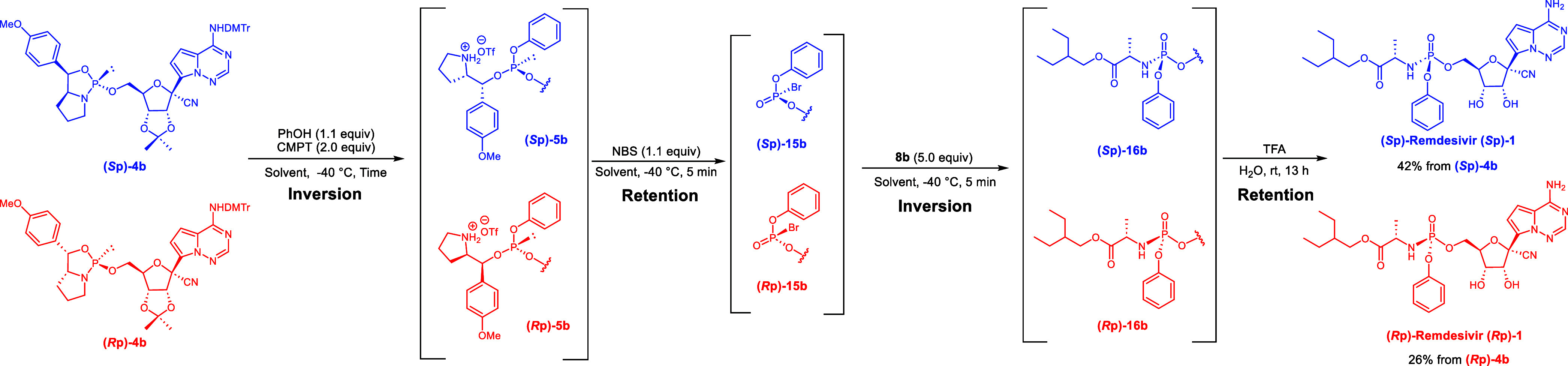
Investigation of the Condensation Conditions of Compound **4b**

Entry^a^	Scale (mmol)	Solvent	Time (min)	^31^P NMR yield of **16b**^b^ (%)	Isolated yield of **1** (%)	dr
1	0.10	CH_3_CN	10	34	-	>99:1^c^
2	0.10	CH_3_CN-toluene	30	51	-	>99:1^c^
3^d^	0.10	CH_3_CN-toluene	60	87	26	>99:1^e^
4^d^	1.1	CH_3_CN-toluene	60	87	42	>99:1^e^

a **(*****R*****p)-4b** and **(*****S*****p)-4b** were used in Entries 1–3 and Entry 4, respectively.

b Determined from the ^31^P NMR spectra of crude mixtures.

c Determined from the ^31^P NMR spectra of crude mixtures.

d Extraction in 0.2 M citric acid aqueous solution.

e Determined from the ^31^P NMR spectra after purification.

(Figure S2), indicating that the condensation reaction was incomplete within 10 min and prompting further investigation of the condensation conditions. As **(*****R*****p)-4b** was not fully dissolved in CH_3_CN, we tried a mixed solvent of CH_3_CN and toluene and extended the condensation reaction time to 60 min (Table 2, Entry 3), obtaining the desired phosphoramidate **16b** as the main product. The 5′-oxazaphospholidine derivative was completely converted to phosphoramidate **16b** within 1.5 h, but separating the desired product from the impurities, mainly comprising a byproduct derived from the reaction of the chiral auxiliary with an amino acid ester, proved challenging. After investigating the extraction conditions for byproduct removal, we found that impurities were effectively removed by washing the organic layer with a 0.2 M citric acid aqueous solution. However, a small amount of phosphoramidate was distributed in the aqueous layer, reducing the isolated yield (Table 2, Entries 3 and 4). The phosphoramidate **16b** residue was deprotected without further purification under acidic conditions. The deprotection reaction was analyze by TLC, and no degradation of the compound was observed. After purification by silica gel column chromatography, the desired compound **1** was isolated in a 39% yield from 5′-oxazaphospholidine derivative **(*****R*****p)-4b** without reducing diastereomer ratio (dr >99:1). One of the reasons for the low yield of the compound **1** is that separation of the product from byproducts by silica gel column chromatography was troublesome and some fraction containing the product and byproducts were discarded. Comparing the ^1^H NMR spectrum with the literature,^17^ the product from **(*****R*****p)-4b** was confirmed as (*R*p)-Remdesivir. As a plausible stereocourse pathway, we propose nucleophilic attack of PhOH on the phosphorus atom of **(*****R*****p)-4b** with inversion of the phosphorus stereochemistry. Bromination of the resultant phosphite triester **5b** with NBS and subsequent amination proceed with retention and inversion, respectively. Based on these results, **13a** and **13b** were identified as (*R*p) and (*S*p) stereoisomers, respectively. After synthesizing (*S*p)-Remdesivir on a 1.0 mmol scale (Table 2, Entry 4), we improved the yield of Remdesivir without loss of the diastereomer ratio, signifying that this synthesis method can realize large-scale synthesis of ProTides. Furthermore, our method requires a much shorter reaction time than conventional synthesis approaches involving 5′-phosphorylation.

Next, we attempted the syntheses of Sofosbuvir **(*****S*****p)-2**, its stereoisomer PSI-7976^8^**(*****R*****p)-2,** and NUC-1031 **3**, which possess non-natural ribose structures. The reactivities of oxazaphospholidine derivatives and PhOH are lower for these compounds than for the model compound and Remdesivir. After optimizing the reaction times, the condensation-reaction completion times of **4c** and **4d** were determined as 2 and 3 h (Table 3), respectively. To obtain Sofosbuvir, PSI-7976, and NUC-1031, we investigated the deprotection conditions of compounds **16c** and **16d**. However, when the TBS group was removed by tetra-*n*-butylammonium fluoride, the product decomposed, as judged by thin-layer chromatography. Consequently, deprotections were conducted using TFA under acidic conditions, successfully obtaining Sofosbuvir **(*****S*****p)-2** (isolated yield from the 5′-oxazaphospholidine derivative: 35%), PSI-7976 **(*****R*****p)-2** (46%), and NUC-1031 **3** (63%) with an excellent stereoselectivities (dr >99:1).

**Table 3:**
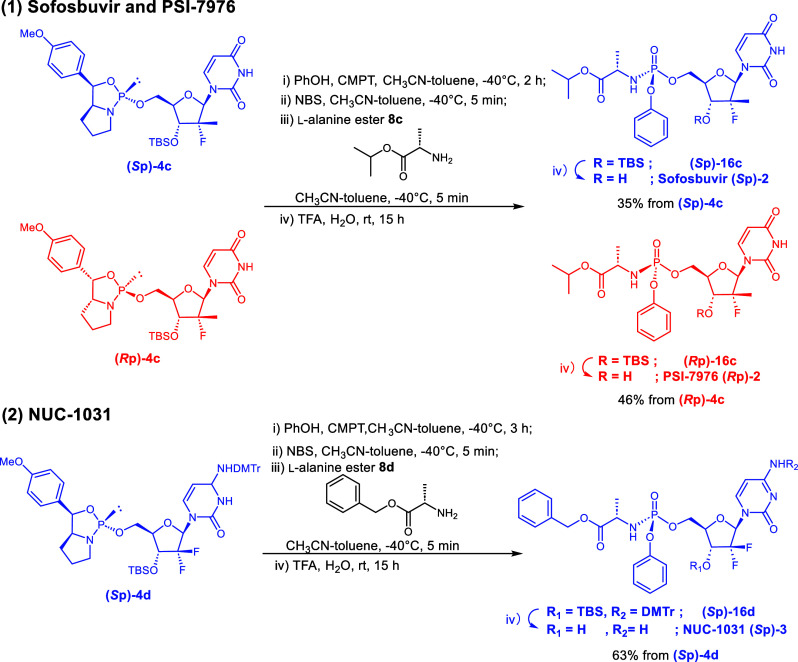
Synthesis Results of Sofosbuvir, PSI-7976, and NUC-1031

Product	Scale (mmol)	^31^P NMR yield of **16**^a^ (%)	Isolated yield of product (%)	dr^b^
**(*****S*****p)-2**	0.16	86	35	>99:1
**(*R*p)-2**	1.0	88	46	>99:1
**(*S*p)-3**	0.10	85	63	>99:1

a Determined from the ^31^P NMR spectra of crude mixtures.

b Determined from the ^31^P NMR spectra after purification.

## Conclusion

The oxazaphospholidine method afforded four kinds of *O-*aryl phosphoramidates and their stereoisomers-two FDA-approved drugs (Remdesivir **(*****S*****p)-1** and Sofosbuvir **(*****S*****p)-2**) and the drug candidate NUC-1031 **3**)—with excellent stereoselectivities (dr >99:1). The desired *O-*aryl phosphoramidates were obtained through the condensation reaction of 5′-oxazaphospholidine derivatives with PhOH, bromination of the resulting phosphite triester using NBS, and subsequent amination. The reactions proceeded under mild conditions within short reaction times and realized the stereoselective synthesis of various ProTides. Furthermore, the synthesis was upscalable to 1.0 mmol with no reduction in stereoselectivity. Although there is a room for improvement of the yields of 5′-oxazaphospholidine derivatives and ProTides, the developed method is expected to be widely applicable to stereoselective syntheses of various phosphoramidate derivatives and should also facilitate drug discovery.

## Experimental Section

### General Information

All the reactions were conducted under Ar atmosphere. Dry organic solvents were prepared by the appropriate relevant procedures. The ^1^H NMR spectra were recorded at 400 or 500 MHz and an internal standard was tetramethylsilane (δ 0.00) or each deuterated solvent signal; CDCl_3_ (δ 7.26), CD_3_CN (δ 1.94), DMSO-*d*_6_ (δ 2.50), or CD_3_OD (δ 3.31). The ^13^C NMR spectra were recorded at 101 or 126 MHz and an internal standard was each deuterated solvent signal; CDCl_3_ (δ 77.16), CD_3_CN (δ 118.26), or DMSO-*d*_6_ (δ 39.52). The ^31^P NMR spectra were recorded at 162 or 202 MHz with 85% H_3_PO_4_ (δ 0.00) as an external standard in CDCl_3_, CD_3_CN, or DMSO-*d*_6_. IR spectra were obtained using an ATR-IR spectrometer. Analytical TLC was performed on commercial glass plated 0.25 mm thickness silica gel layer. Manual silica gel column chromatography was performed using spherical, neutral, 63–210 μm silica gel unless otherwise noted. Automated silica gel column chromatography was performed on amino silica gel (Yamazen UNIVERSAL Premium column (30 μm)) (Yamazen Corporation) using automated flash chromatography system W-prep 2XY (Yamazen Corporation). The detections in RP-HPLC were achieved at 286 nm at a temperature of 30 °C and a flow rate of 0.5 mL/min using a C18 column (5 μm, 100 Å, 3.9 × 150 mm). GS-441524 and amino acid esters were purchased from Sapala Organics and TCI (Tokyo Chemical Industry Co., Ltd.). 5**’**-Oxazaphospholidine derivatives were dissolved in toluene and dried over MS 4A for 2–24 h. CMPT and PhOH were dissolved in CH_3_CN and dried over MS 3A for 24 h.

#### 2-Ethylbutyl l-alaninate (**8b**)

2-Ethylbutyl l-alaninate hydrochloride **8a** (76.3 mg, 0.49 mmol) and KHCO_3_ (76.3 mg, 0.76 mmol) was dissolved in CH_3_CN (0.5 mL) at room temperature and stirred for 6 h. The mixture was filtered to afford compound **8b**. This filtrate was used for amination without further purification.

#### 2-Isopropyl l-alaninate (**8c**)

The same procedure for the synthesis of compound **8b** was applied for isopropyl l-alaninate hydrochloride (84.1 mg, 0.5 mmol) to afford compound **8c**.

#### Benzyl l-alaninate (**8d**)

The same procedure for the synthesis of compound **8b** was applied for benzyl l-alaninate hydrochloride (108.6 mg, 0.5 mmol) to afford compound **8d**.

##### 2-Ethylbutyl((*R*)-(((3a*R*,4*R*,6*R*,6a*R*)-6-(6-((bis(4-methoxyphenyl)(phenyl)methyl)amino)-9*H*-purin-9-yl)-2,2-dimethyltetrahydrofuro[3,4-*d*][1,3]dioxol-4-yl)methoxy)(phenoxy)phosphoryl)-l-alaninate (**13a**)

Compound **(*****R*****p)-4a** (84.4 mg, 0.10 mmol) was dissolved in CD_3_CN (0.3 mL), dried over MS 3A for 24 h, and cooled to −40 °C. A mixture of CMPT (52.3 mg, 0.20 mmol) and PhOH (9.3 mg, 0.10 mmol) in CD_3_CN (0.3 mL), which was dried over MS 3A for 24 h, was added to the solution. After stirred for 10 min at −40 °C, NBS (19.6 mg, 0.11 mmol) in CD_3_CN (0.1 mL) was added to the reaction mixture at −40 °C and stirred for 5 min, then 3.3 M CD_3_CN solution of l-alanine ester **8b** (0.15 mL, 5.0 mmol) was added to the reaction mixture and stirred at room temperature for 3 h. The mixture was diluted with EtOAc (10 mL) and washed with saturated NaHCO_3_ aqueous solution (3 × 10 mL). The organic layer was dried Na_2_SO_4_, filtered, and concentrated under reduced pressure. The residue was purified by silica gel column chromatography (neutral silica gel, 40 g, toluene–EtOAc 8:2–7:3, v/v) as an eluent and preparative TLC (hexane–EtOAc 7:3, v/v) to give **10a** as a colorless foam (23.0 mg, 25%) (dr = 99:1).

^1^H NMR (500 MHz, CDCl_3_) δ 8.01 (s, 1H), 7.85 (s, 1H), 7.27–7.25 (m, 2H), 7.18–7.15 (m, 6H), 7.11 (dt, *J* = 10.2, 1.3 Hz, 2H), 7.02 (dt, *J* = 8.0, 0.7 Hz, 1H), 6.94–6.93 (m, 2H), 6.83 (s, 1H), 6.71 (dt, *J* = 8.9, 2.9 Hz, 4H), 5.97 (d, *J* = 2.6 Hz, 1H), 4.92 (dd, *J* = 6.2, 2.4 Hz, 1H), 4.77 (dd, *J* = 6.3, 2.6 Hz, 1H), 4.44–4.42 (m, 1H), 4.31–4.27 (m, 1H), 4.22–4.18 (m, 1H), 3.99–3.91 (m, 3H), 3.69 (s, 6H), 3.57 (t, *J* = 10.6 Hz, 1H), 1.50 (s, 3H), 1.45–1.40 (m, 1H), 1.29–1.22 (m, 11H), 0.80–0.78 (td, *J* = 7.6, 0.6 Hz, 6H); ^13^C{^1^H} NMR (126 MHz, CDCl_3_) δ 173.8 (d, ^3^*J*_PC_ = 7.7 Hz), 158.4, 154.3, 152.7, 150.7 (d, ^2^*J*_PC_ = 6.8 Hz), 148.4, 145.5, 138.9, 137.6, 137.5, 131.1, 130.2, 129.8, 128.9, 127.0, 125.1, 121.6, 120.1 (d, ^3^*J*_PC_ = 5.0 Hz), 114.5, 113.3, 91.6, 85.3 (d, ^3^*J*_PC_ = 8.0 Hz), 84.3, 81.3, 70.8, 67.7, 66.5, (d, ^2^*J*_PC_ = 5.1 Hz), 55.4, 50.5 (d, ^2^*J*_PC_ = 1.5 Hz), 40.3, 29.8, 27.2, 25.2, 23.3, 23.3, 21.1 (d, ^3^*J*_PC_ = 4.6 Hz), 11.1, 11.1; ^31^P{^1^H} NMR (202 MHz, CDCl_3_) δ 3.23; IR (neat, cm^–1^) 2932, 1737, 1602, 1508, 1469, 1373, 1209, 1179, 1154, 1031, 930, 827, 768, 690, 647, 582, 508; HRMS (ESI/QTOF) [M + H]^+^ Calcd for C_49_H_58_N_6_O_10_P 921.3946; Found 921.3956.

##### 2-Ethylbutyl (*S*)-(((3a*R*,4*R*,6*R*,6a*R*)-6-(6-((bis(4-methoxyphenyl)(phenyl)methyl)amino)-9*H*-purin-9-yl)-2,2-dimethyltetrahydrofuro[3,4-*d*][1,3]dioxol-4-yl)methoxy)(phenoxy)phosphoryl)-l-alaninate (**13b**)

Compound **(*****S*****p)-4a** (168.8 mg, 0.20 mmol) was dissolved in toluene (1.4 mL), dried over MS 4A for 24 h, cooled to −40 °C. A mixture of CMPT (102.5 mg, 0.39 mmol) and PhOH (19.0 mg, 0.20 mmol) in CH_3_CN (0.7 mL), which was dried over MS 3A for 24 h, was added over 1 min to the solution. After stirred for 10 min at −40 °C, NBS (39.5 mg, 0.22 mmol) in CH_3_CN (0.6 mL) was added to the reaction mixture at −40 °C and stirred for 5 min, then 1.0 M CH_3_CN solution of l-alanine ester **8b** (1.0 mL, 1.0 mmol) was added to the reaction mixture and stirred at room temperature for 6 min the mixture was diluted with CH_2_Cl_2_ (30 mL) and washed with 0.2 M citric acid aqueous solution (30 mL) and saturated NaHCO_3_ aqueous solution (30 mL). The organic layer was dried Na_2_SO_4_, filtered, and concentrated under reduced pressure to give crude of **10b** (223.7 mg). The residue (168.6 mg) was purified by silica gel column chromatography (neutral silica gel 40–50 μm, 10 g, toluene–EtOAc (7:3, v/v) containing a 1% triethylamine) as an eluent to give **10b** as a colorless foam (53.5 mg, 39%) (dr >99:1).

^1^H NMR (400 MHz, CDCl_3_) δ 8.08 (s, 1H), 7.93 (s, 1H), 7.33 (dt, *J* = 8.0, 1.7 Hz, 2H), 7.27–7.21 (m, 6H), 7.19 (dt, *J* = 10.2, 1.3 Hz, 2H), 7.11–7.07 (m, 1H), 7.02–7.00 (m, 2H), 6.91 (s, 1H), 6.78 (dt, *J* = 8.9, 2.9 Hz, 4H), 6.04 (d, *J* = 2.6 Hz, 1H), 4.99 (dd, *J* = 6.3, 2.6 Hz, 1H), 4.84 (dd, *J* = 6.3, 2.6 Hz, 1H), 4.51–4.49 (m, 1H), 4.38–4.34 (m, 1H), 4.29–4.24 (m, 1H), 4.06–3.98 (m, 3H), 3.76 (s, 6H), 3.66 (t, *J* = 10.0 Hz, 1H), 1.60 (s, 3H), 1.53–1.46 (m, 1H), 1.36–1.26 (m, 11H), 0.87 (dt, *J* = 3.1, 0.5 Hz, 6H); ^13^C{^1^H} NMR (126 MHz, CDCl_3_) δ 173.8 (d, ^3^*J*_PC_ = 7.8 Hz), 158.4, 154.3, 152.7, 150.7 (d, ^2^*J*_PC_ = 7.1 Hz), 148.3, 145.5, 138.9, 137.5, 137.5, 130.2, 129.7, 128.9, 128.0, 126.9, 125.1, 121.6, 120.2 (d, ^3^*J*_PC_ = 4.7 Hz), 114.5, 113.3, 91.6, 85.3 (d, ^3^*J*_PC_ = 8.2 Hz), 84.3, 81.3, 70.8, 67.7, 66.4 (d, ^2^*J*_PC_ = 5.0 Hz), 55.3, 50.5 (d, ^2^*J*_PC_ = 1.5 Hz), 40.3, 27.1, 25.2, 23.3, 23.3, 21.1 (d, ^3^*J*_PC_ = 4.9 Hz), 11.1, 11.1; ^31^P{^1^H} NMR (202 MHz, CDCl_3_) δ 3.31; IR (neat, cm^–1^) 2958, 2983, 1737, 1602, 1508, 1491, 1469, 1373, 1292, 1210, 1179, 1154, 1068, 1031, 930, 866, 827, 768, 690, 646, 582, 510; HRMS (ESI/QTOF) [M + H]^+^ Calcd for C_49_H_58_N_6_O_10_P 921.3946; Found 921.3948.

### General Procedure of the Synthesis of ProTides

Compound **(*****S*****p)-4b** (0.95 g, 1.1 mmol), **(*****R*****p)-4b** (88.4 mg, 0.10 mmol), **(*****S*****p)-4c** (98.5 mg, 0.16 mmol), **(*****R*****p)-4c** (0.61 g, 1.0 mmol), and **(*****S*****p)-4d** (90.3 mg, 0.10 mmol) was dissolved in toluene (0.7 mL for the synthesis of **(*****R*****p)-1** and **3**; 7.0 mL for the synthesis of **(*****S*****p)-1** and **(*****R*****p)-2**; and 1.4 mL for the **(*****S*****p)-2**), dried over MS 4A for 2 h, and cooled to −40 °C. A mixture of CMPT (0.56 g, 2.2 mmol for **(*****S*****p)-1**; 51.1 mg, 0.20 mmol for **(*****R*****p)-1**; 86.2 mg, 0.33 mmol for **(*****S*****p)-2**; 0.52 g, 2.0 mmol for **(*****R*****p)-2**; and 53.5 mg, 0.21 mmol for **3**) and PhOH (0.11 g, 1.2 mmol for **(*****S*****p)-1**; 11.2 mg, 0.12 mmol for **(*****R*****p)-1**; 16.8 mg, 0.18 mmol for **(*****S*****p)-2**; 0.10 g, 1.1 mmol for **(*****R*****p)-2**; and 11.3 mg, 0.12 mmol for **3**) in CH_3_CN (3,0 mL for **(*****S*****p)-1** and **(*****R*****p)-2**; 0.3 mL for **(*****R*****p)-1** and **3**; and 0.6 mL for **(*****S*****p)-2**), which was dried over MS 3A for 24 h, was added to the solution. After stirred for designated time (1 h for **(*****S*****p)-1** and **(*****R*****p)-1**; 2 h for **(*****S*****p)-2** and **(*****R*****p)-2**; and 3 h for **3)** at −40 °C, NBS (194.9 mg, 1.1 mmol for **(*****S*****p)-1**; 19.7 mg, 0.11 mmol for **(*****R*****p)-1**; 0.45 mg, 0.16 mmol for **(*****S*****p)-2**; 0.20 g, 1.1 mmol for **(*****R*****p)-2**; and 19.8 mg, 0.11 mmol for **3**) in CH_3_CN (3.0 mL for **(*****S*****p)-1** and **(*****R*****p)-2**; 0.3 mL for **(*****R*****p)-1** and **3**; and 0.6 mL for **(*****S*****p)-2**) was added to the reaction mixture at −40 °C and stirred for 5 min, then CH_3_CN solution of l-alanine ester (**8b** (5.0 mL, 5.0 mmol) for **(*****S*****p)-1**; **8b** (1.0 mL, 0.05 mmol) for **(*****R*****p)-1**; **8c** (1.0 mL, 1.0 mmol) for **(*****S*****p)-2**; **8c** (5.0 mL, 5.0 mmol) for **(*****R*****p)-2**; and **8d** (0.5 mL, 0.5 mmol) for **3**) was added to the reaction mixture and stirred at room temperature for 5 min.

#### (***S*****p**)-Remdesivir **(*****S*****p)-1**

The mixture was diluted with EtOAc (100 mL) and washed with 0.2 M citrate acid aqueous solutions (2 × 50 mL) and saturated NaHCO_3_ aqueous solutions (2 × 50 mL). The organic layer was dried MgSO_4_, filtered, and concentrated under reduced pressure. The residue was diluted with H_2_O (2.0 mL) and cooled to 0 °C. TFA-H_2_O (36:2 v/v) (38 mL) was added dropwise to the solution and stirred for 13 h. The reaction mixture was concentrated to a small volume under reduced pressure and neutralized with cooled saturated NaHCO_3_ aqueous solution until pH 7. The aqueous solution was extracted with EtOAc (3 × 100 mL) and the organic layers were combined. The organic layer was dried MgSO_4_, filtered, and concentrated under reduced pressure. The residue was purified by automated silica gel column chromatography (amino silica gel, 40 g L size) using a linear gradient of CH_2_Cl_2_–MeOH (92:8–86:14, v/v) as an eluent to give **(*****S*****p)-1** as a colorless foam (0.27 g, 42%) (dr >99:1). The ^1^H and ^31^P spectra corresponded with the literature data.^17^

^1^H NMR (500 MHz, DMSO-*d*_6_) δ 8.0–7.8 (br, 3H), 7.35 (t, *J* = 7.3 Hz, 2H), 7.20–7.16 (m, 3H), 6.89 (d, *J* = 4.5 Hz, 1H), 6.83 (d, *J* = 4.5 Hz, 1H), 6.33 (d, *J* = 6.0 Hz, 1H), 6.04 (dd, *J* = 13.0, 10.1 Hz, 1H), 5.37 (d, *J* = 5.8 Hz, 1H), 4.64 (t, *J* = 5.4 Hz, 1H), 4.46–4.44 (m, 1H), 4.28–4.21 (m, 2H), 4.11–4.06 (m, 1H), 3.97–3.92 (m, 2H), 3.88–3.79 (m, 2H), 1.45–1.37 (m, 1H), 1.27–1.20 (m, 7H), 0.79 (t, *J* = 7.4 Hz, 6H); ^13^C{^1^H} NMR (126 MHz, DMSO-*d*_6_) δ 173.3 (d, ^3^*J*_PC_ = 4.0 Hz), 155.6, 150.7 (d, ^2^*J*_PC_ = 6.4 Hz), 147.9, 129.6, 124.5, 123.5, 120.2, 120.1, 117.0, 116.6, 116.4, 110.4, 100.9, 82.2 (d, ^2^*J*_PC_ = 8.3 Hz), 78.9, 74.1, 69.9, 66.1, 65.1 (d, ^3^*J*_PC_ = 4.2 Hz), 62.8, 62.6, 49.9, 22.6, 22.6, 19.7 (d, ^3^*J*_PC_ = 7.2 Hz), 10.8, 10.8; ^31^P{^1^H} NMR (202 MHz, DMSO-*d*_6_) δ 4.85; HRMS (ESI/QTOF) [M + H]^+^ Calcd for C_27_H_36_N_6_O_8_P 603.2327; Found 603.2317.

#### (***R*****p**)-Remdesivir **(*****R*****p)-1**

The mixture was diluted with EtOAc (20 mL) and washed with 0.2 M citrate acid aqueous solutions (3 × 20 mL) and saturated NaHCO_3_ aqueous solutions (2 × 20 mL). The organic layer was dried MgSO_4_, filtered, and concentrated under reduced pressure. The residue was diluted with H_2_O (0.2 mL) and cooled to 0 °C by ice bath. TFA-H_2_O (36:2 v/v) (3.8 mL) was added dropwise to the solution and stirred for 16 h. The reaction mixture was concentrated to a small volume under reduced pressure and neutralized with cooled saturated NaHCO_3_ aqueous solution until pH 7. The aqueous solution was extracted with EtOAc (2 × 50 mL) and the organic layers were combined. The organic layer was dried MgSO_4_, filtered, and concentrated under reduced pressure. The residue was purified by automated silica gel column chromatography (amino silica gel, 40 g L size) using a linear gradient of CH_2_Cl_2_–MeOH (92:8–86:14, v/v) as an eluent to give **(*****R*****p)-1** as a colorless foam (15.7 mg, 26%) (dr >99:1). The ^1^H, ^13^C, and ^31^P NMR spectra corresponded with the literature data.^17^

^1^H NMR (500 MHz, DMSO-*d*_6_) δ 8.5–8.3 (br, 1H), 8.3–8.2 (br, 1H), 8.00 (s, 1H), 7.33 (t, *J* = 5.2 Hz, 2H), 7.17–7.13 (m, 3H), 7.01 (d, *J* = 4.5 Hz, 1H), 6.88 (d, *J* = 4.5 Hz, 1H), 6.03 (dd, *J* = 13.3, 10.0 Hz, 1H), 4.63 (d, *J* = 4.9 Hz, 1H), 4.29–4.25 (m, 2H), 4.15–4.10 (m, 1H), 3.97–3.88 (m, 2H), 3.79–3.72 (m, 1H), 1.47–1.40 (m, 1H), 1.29–1.23 (m, 4H), 1.16 (d, *J* = 7.0 Hz, 3H), 0.80 (t, *J* = 7.4 Hz, 6H); ^13^C{^1^H} NMR (126 MHz, DMSO-*d*_6_) δ 173.3 (d, ^3^*J*_PC_ = 4.0 Hz), 155.6, 150.7 (d, ^2^*J*_PC_ = 6.4 Hz), 147.9, 129.5, 124.5, 123.5, 120.2, 120.1, 117.0, 117.0, 116.6, 110.4, 100.9, 82.3 (d, ^2^*J*_PC_ = 8.3 Hz), 78.7, 74.1, 69.9, 66.1, 62.8, 62.6, 49.9, 22.6, 22.5, 19.6 (d, ^3^*J*_PC_ = 7.3 Hz), 10.8, 10.8; ^31^P{^1^H} NMR (202 MHz, DMSO-*d*_6_) δ 4.83; HRMS (ESI/QTOF) [M + H]^+^ Calcd for C_27_H_36_N_6_O_8_P 603.2327; Found 603.2314.

#### Sofosbuvir **(*****S*****p)-2**

The mixture was diluted with EtOAc (30 mL) and washed with 0.2 M citrate acid aqueous solutions (3 × 10 mL) and saturated NaHCO_3_ aqueous solutions (3 × 10 mL). The organic layer was dried MgSO_4_, filtered, and concentrated under reduced pressure. The residue was diluted with H_2_O (0.4 mL) and cooled to 0 °C by ice bath. TFA-H_2_O (36:2 v/v) (7.6 mL) was added dropwise to the solution and stirred for 16 h. The reaction mixture was neutralized with cooled saturated NaHCO_3_ aqueous solution until pH 7. The aqueous solution was extracted with EtOAc (2 × 100 mL) and the organic layers were combined. The organic layer was dried MgSO_4_, filtered, and concentrated under reduced pressure. The residue was purified with silica gel column chromatography (neutral silica gel 40–50 μm, 15 g, CH_2_Cl_2_–MeOH = 98:2–95:5, v/v) as an eluent to give Sofosbuvir **(*****S*****p)-2** as a colorless foam (30.0 mg, 35%) (dr >99:1). The ^1^H, ^13^C, and ^31^P NMR spectra corresponded with the literature data.^18^

^1^H NMR (500 MHz, DMSO-*d*_6_) δ 11.53 (brs, 1H), 7.56 (d, *J* = 7.2 Hz, 1H), 7.37 (t, *J* = 7.9 Hz, 2H), 7.22 (d, *J* = 8.4 Hz, 2H), 7.18 (t, *J* = 7.3 Hz, 1H), 6.06 (dd, *J* = 12.6, 10.4 Hz, 2H), 5.85 (d, *J* = 5.1 Hz, 1H), 5.54 (d, *J* = 8.0 Hz, 1H), 4.89–4.82 (m, 1H), 4.39–4.35 (m, 1H), 4.26–4.21 (m, 1H), 4.02–3.99 (m, 1H), 3.85–3.70 (m, 2H), 1.27–1.22 (m, 6H), 1.15 (d, *J* = 6.2 Hz, 6H); ^13^C{^1^H} NMR (126 MHz, DMSO-*d*_6_) δ 172.6 (d, ^3^*J*_PC_ = 5.2 Hz), 162.6, 157.6, 150.7 (d, ^2^*J*_PC_ = 6.3 Hz), 150.4, 129.6, 124.6, 120.1 (d, *J* = 4.7 Hz), 102.3, 100.3 (d, *J* = 181.2 Hz), 71.4, 68.0, 64.7, 49.8, 21.4 (d, *J* = 4.5 Hz), 19.8 (d, ^3^*J*_PC_ = 6.5 Hz), 16.5 (d, *J* = 25.3 Hz); ^31^P{^1^H} NMR (202 MHz, DMSO-*d*_6_) δ 4.97; HRMS (ESI/QTOF) [M + Na]^+^ Calcd for C_23_H_29_FN_3_NaO_9_P 552.1523; Found 552.1503.

#### PSI-7976 **(*R*p)-2**

The mixture was diluted with EtOAc (100 mL) and washed with 0.2 M citrate acid aqueous solutions (3 × 50 mL) and saturated NaHCO_3_ aqueous solutions (2 × 50 mL). The organic layer was dried MgSO_4_, filtered, and concentrated under reduced pressure. The residue was diluted with H_2_O (2.0 mL) and cooled to 0 °C by ice bath. TFA-H_2_O (36:2 v/v) (38 mL) was added dropwise to the solution and stirred for 16 h. The reaction mixture was neutralized with cooled saturated NaHCO_3_ aqueous solution until pH 7. The aqueous solution was extracted with EtOAc (3 × 150 mL) and the organic layers were combined. The organic layer was dried MgSO_4_, filtered, and concentrated under reduced pressure. The residue was purified with silica gel column chromatography (neutral silica gel 40–50 μm, 20 g, CH_2_Cl_2_–MeOH = 98:2–95:5, v/v) as an eluent to give PSI-7976 **(*****R*****p)-2** as a colorless foam (0.24 g, 46%) (dr >99:1). The ^1^H, ^13^C, and ^31^P NMR spectra corresponded with the literature data.^18^

^1^H NMR (500 MHz, DMSO-*d*_6_) δ 7.56 (d, *J* = 7.7 Hz, 1H), 7.37 (t, *J* = 7.9 Hz, 2H), 7.20–7.16 (m, 3H), 6.13–6.02 (m, 2H), 5.92 (s, 1H), 5.57 (d, *J* = 8.1 Hz, 1H), 4.88–4.81 (m, 1H), 4.41 (dd, *J* = 11.0, 4.6 Hz, 1H), 4.29–4.24 (m, 1H), 4.05 (dd, *J* = 6.4, 2.9 Hz, 1H), 3.78–3.70 (m, 2H), 1.26 (s, 1H), 1.22–1.18 (m, 5H), 1.15 (dd, *J* = 6.2, 3.6 Hz, 6H);^13^C{^1^H} NMR (126 MHz, DMSO-*d*_6_) δ 172.6 (d, ^3^*J*_PC_ = 5.3 Hz), 162.8, 150.7 (d, ^2^*J*_PC_ = 6.3 Hz), 150.4, 129.7, 124.6, 120.0 (d, *J* = 4.8 Hz), 102.3, 100.3 (d, *J* = 181.0 Hz), 71.5, 68.0, 64.6, 49.8, 21.4 (d, *J* = 4.4 Hz), 19.8 (d, ^3^*J*_PC_ = 6.6 Hz), 16.5 (d, *J* = 25.3 Hz); ^31^P{^1^H} NMR (202 MHz, DMSO-*d*_6_) δ 4.29; HRMS (ESI/QTOF) [M + Na]^+^ Calcd for C_23_H_29_FN_3_NaO_9_P 552.1523; Found 552.1509.

#### NUC-1031 (**3**)

The mixture was diluted with EtOAc (25 mL) and washed with 0.2 M citrate acid aqueous solutions (3 × 25 mL). The organic layer was dried MgSO_4_, filtered, and concentrated under reduced pressure. The residue was diluted with H_2_O (0.2 mL) and cooled to 0 °C by ice bath. TFA-H_2_O (36:2 v/v) (3.8 mL) was added dropwise to the solution and stirred for 14 h. The reaction mixture was neutralized with cooled saturated NaHCO_3_ aqueous solution until pH 7. The aqueous solution was extracted with EtOAc (3 × 50 mL) and the organic layers were combined. The organic layer was dried MgSO_4_, filtered, and concentrated under reduced pressure. The residue was purified with silica gel column chromatography (neutral silica gel 20 g, CH_2_Cl_2_–MeOH = 98:2–85:15, v/v) as an eluent to give NUC-1031 **3** as a colorless foam (36.6 mg, 63%) (dr >99:1). The ^1^H, ^13^C, and ^31^P NMR spectra corresponded with the literature data.^18^

^1^H NMR (500 MHz, CD_3_OD) δ 7.81 (d, *J* = 7.5 Hz, 1H), 7.39–7.32 (m, 7H), 7.23–7.17 (m, 3H), 6.23 (t, *J* = 8.2 Hz, 1H), 5.92 (d, *J* = 7.5 Hz, 1H), 5.15 (s, 2H), 5.09–5.00 (m, 1H), 4.07–3.98 (m, 2H), 3.94 (dd, *J* = 6.4, 1.9 Hz, 2H), 3.86 (dd, *J* = 12.8, 3.1 Hz, 1H), 1.38 (dd, *J* = 7.1, 1.0 Hz, 3H); ^13^C{^1^H} NMR (126 MHz, CD_3_OD) δ 174.5 (d, ^3^*J*_PC_ = 4.6 Hz), 167.6, 157.6, 151.9 (d, ^2^*J*_PC_ = 7.2 Hz), 142.6, 137.3, 130.8, 129.6, 129.4, 129.4, 126.3, 124.8 (d, *J* = 3.7 Hz), 122.7, 122.7, 122.7 (d, *J* = 3.7 Hz), 96.6, 86.1 (t, *J =* 35.1 Hz), 81.1 (d, *J* = 4.4 Hz), 79.5, 73.6 (td, J = 5.2 Hz), 68.0, 60.0, 51.7, 20.3 (d, ^3^*J*_PC_ = 7.5 Hz); ^31^P{^1^H} NMR (202 MHz, CD_3_OD) δ 3.93; HRMS (ESI/QTOF) [M + H]^+^ Calcd for C_25_H_28_F_2_N_4_O_8_P 581.1607; Found 581.1598.

## Data Availability

The data underlying this study are available in the published article and its online Supporting Information.

## Supporting Information Available

Experimental details, HPLC profiles, copies of ^1^H, ^13^C, and ^31^P NMR spectra of new compounds and final products (PDF)
