# Correction to “Stereoselective Synthesis of Nucleotide Analog Prodrugs (ProTides) via an Oxazaphospholidine Method”

**DOI:** 10.1021/acs.joc.5c02213

**Published:** 2025-10-16

**Authors:** Monta Nakamura, Kiyoshi Kakuta, Kazuki Sato, Takeshi Wada

In the original publication, the stereo­chemistries of the 5′-oxaza­phos­phol­idine derivatives **4b**–**4d** were incorrect. Our re-evaluation of the stereo­chemical pathway of phosphor­amidates from 5′-oxaza­phos­pho­lidine derivatives revealed an inconsistency in the original manuscript. To address this, we conducted a detailed review of the compounds used in this study.

Then, we became aware that we utilized (*R*p)-5′-oxaza­phos­phol­idine derivatives as (*S*p)-counterparts, and vice versa. Thus, (*S*p)-ProTides were not synthesized from “(*S*p)-5′-oxaza­phos­phol­idine derivatives” but from “(*R*p)-5′-oxaza­phos­phol­idine derivatives”, and the original assignments of 5′-oxaza­phos­phol­idine derivatives **(** ***R*** **p)-4b**, **(** ***R*** **p)-4c**, **(** ***S*** **p)-4b**, **(** ***S*** **p)-4c**, and **(** ***S*** **p)-4d** in Tables 2 and 3 should be replaced as **(** ***S*** **p)-4b**, **(** ***S*** **p)-4c**, **(** ***R*** **p)-4b**, **(** ***R*** **p)-4c**, and **(** ***R*** **p)-4d**.

On the other hand, the stereo­chemical assignment of the 5′-oxaza­phos­phol­idine derivative **(** ***R*** **p)-4a** in the original Schemes 2 and 3 and Table 1 was correct; however, the stereo­chemical assignments of the intermediates were incorrect. Based on the stereo­chemical pathway from 5′-oxaza­phos­phol­idine derivatives to ProTides, the (*S*p)-model compound **13a** and the (*R*p)-model compound **13b** were synthesized from the (*R*p)-5′-oxaza­phos­phol­idine derivative **(** ***R*** **p)-4a** and the (*S*p)-5′-oxaza­phos­phol­idine derivative **(** ***S*** **p)-4a**, respectively.

These errors also affect the graphical abstract, Experimental Section, and Supporting Information. We present herein the corrected stereochemistries of these compounds (Schemes 1–3 and Tables 1–3); the corrected experimental details for **13a**, **13b**, and the general procedure for the synthesis of ProTides; and revised Supporting Information with the corrected ^1^H, ^13^C, and ^31^P NMR spectra and experimental details for the 5′-oxaza­phos­phol­idine derivatives.

We express sincere regret for these errors in the published article.

Corrected Table of Contents graphic/graphical Abstract:
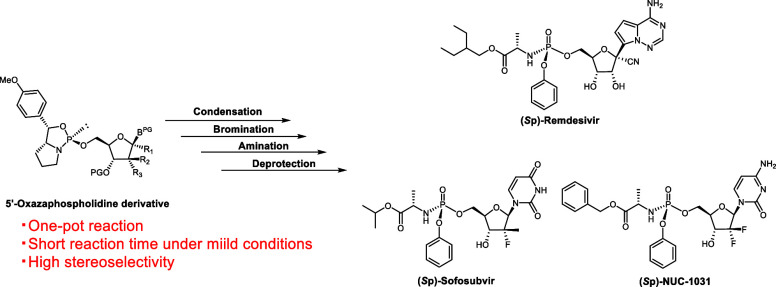


**Scheme 1:**
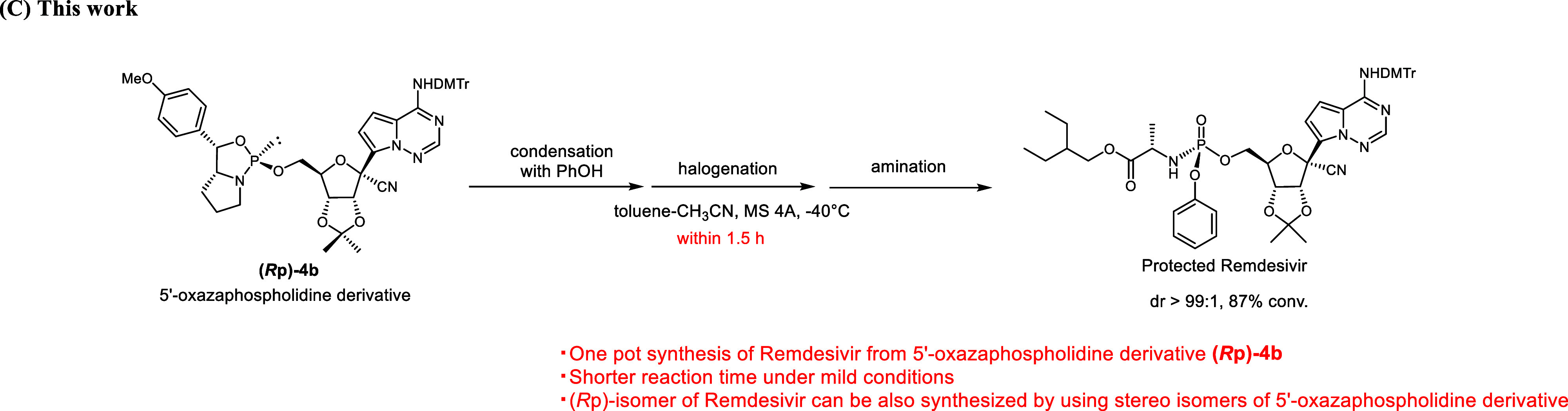
Stereoselective Synthesis of Remdesivir (Part C, Corrected)

**2:**
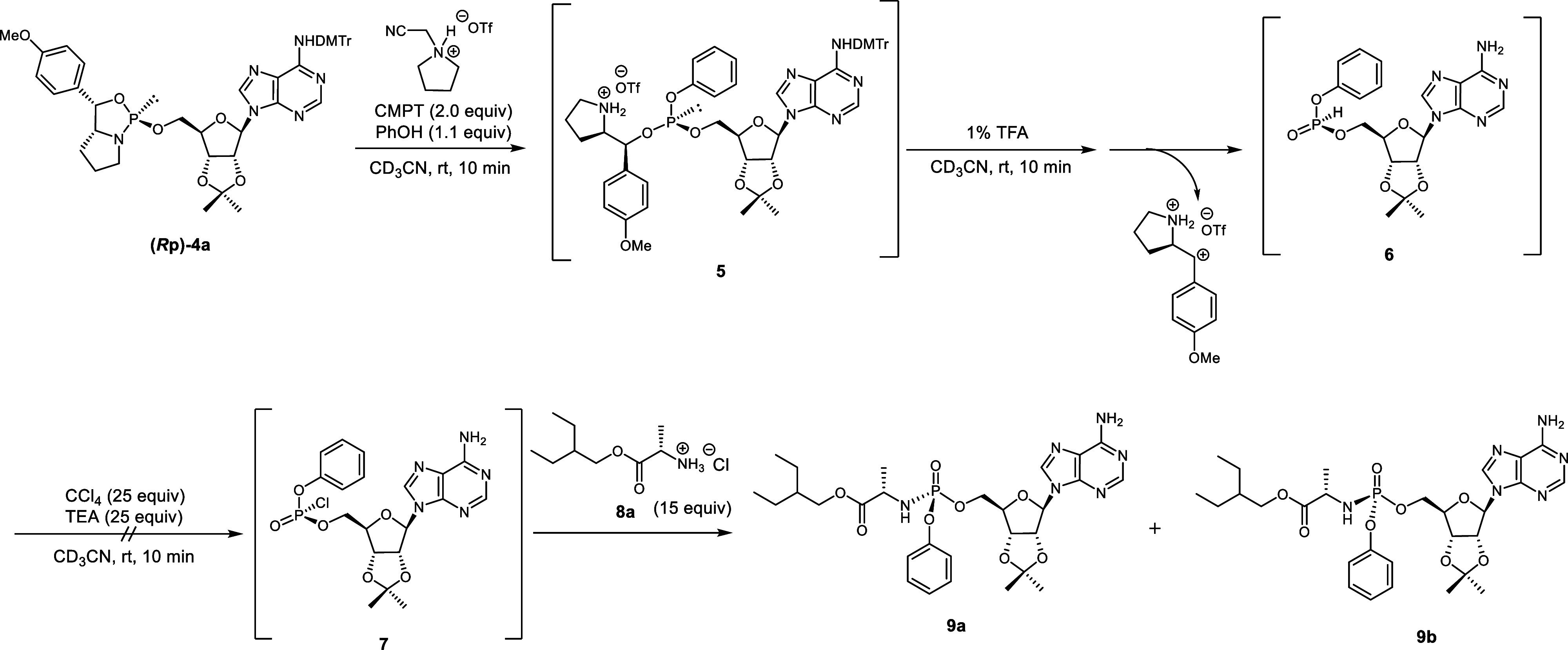
Synthesis of the Model Compound via an H-Phosphonate (Corrected)

**Scheme 3:**
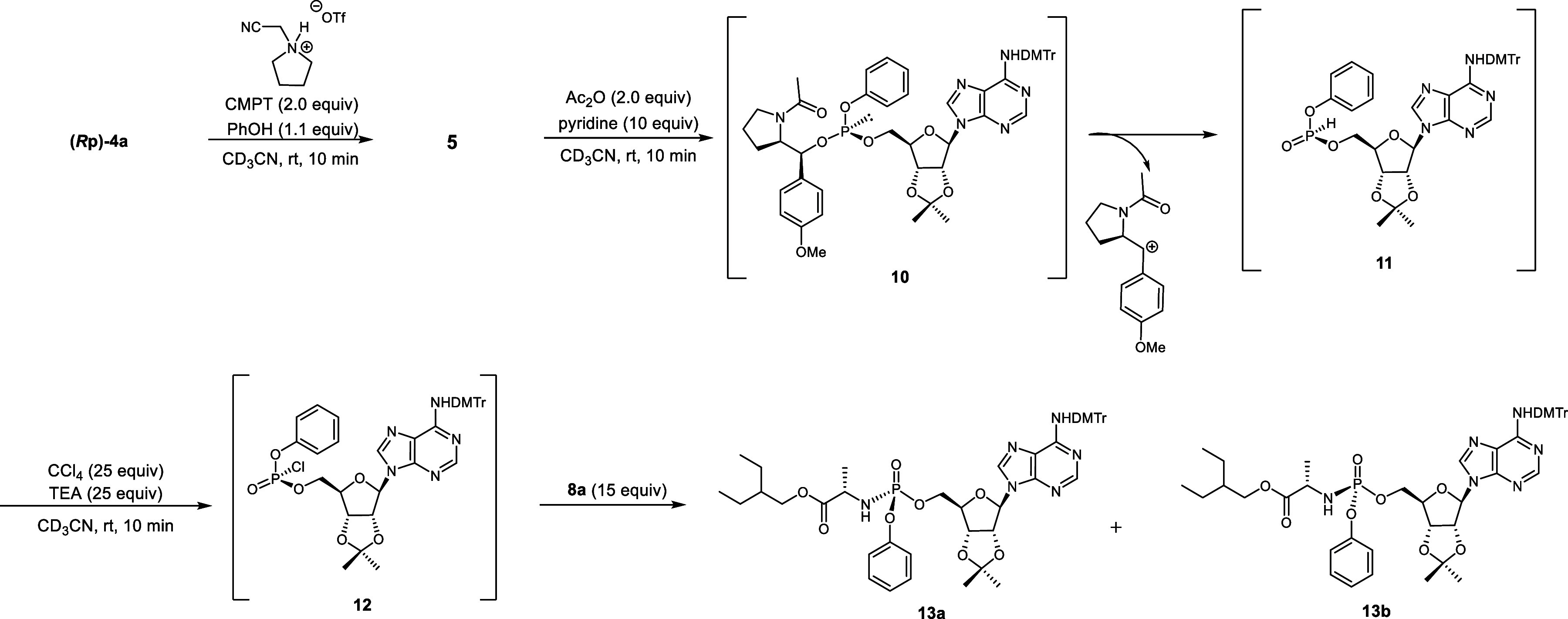
Synthesis of the Model Compound via Acylation of a Phosphite Triester (Corrected)

**Table 1:**
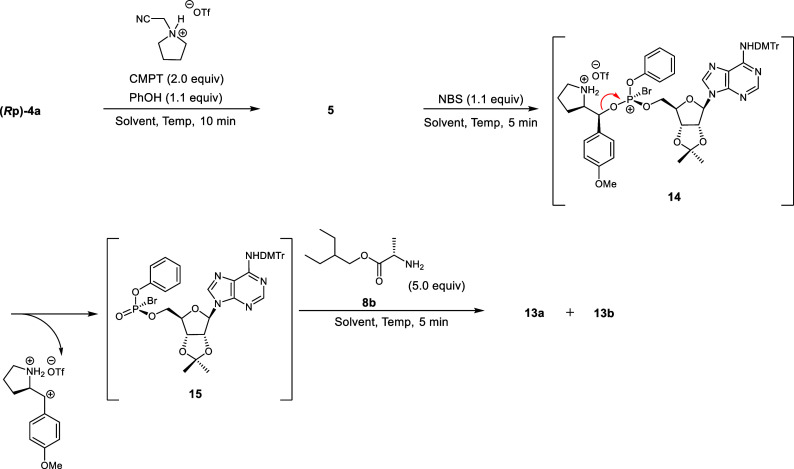
Investigation of Halogenation Reaction Conditions (Corrected)

Entry^a^	Solvent	Temp (°C)	^31^P NMR yield of **13** (%)	Isolated yield of **13** (%)	dr^b^
1	CD_3_CN	0	28	–	70:30
2	CD_3_CN	–20	36	16	88:12
3	CD_3_CN	–40	53	25	>99:1
4	CH_3_CN-toluene^c^	–40	78	39	>99:1

a Entries 1–3 and Entry 4 were conducted on 0.03 and 0.18 mmol scales, respectively.

b Entry 1: Determined from the ^31^P NMR spectrum of crude mixture. Entries 2 and 3: Determined from the ^31^P NMR spectra after purification. Entry 4 was determined from RP-HPLC after deprotection of the DMTr group of the model compound.

c All reagents were added as CH_3_CN solution.

**2:**
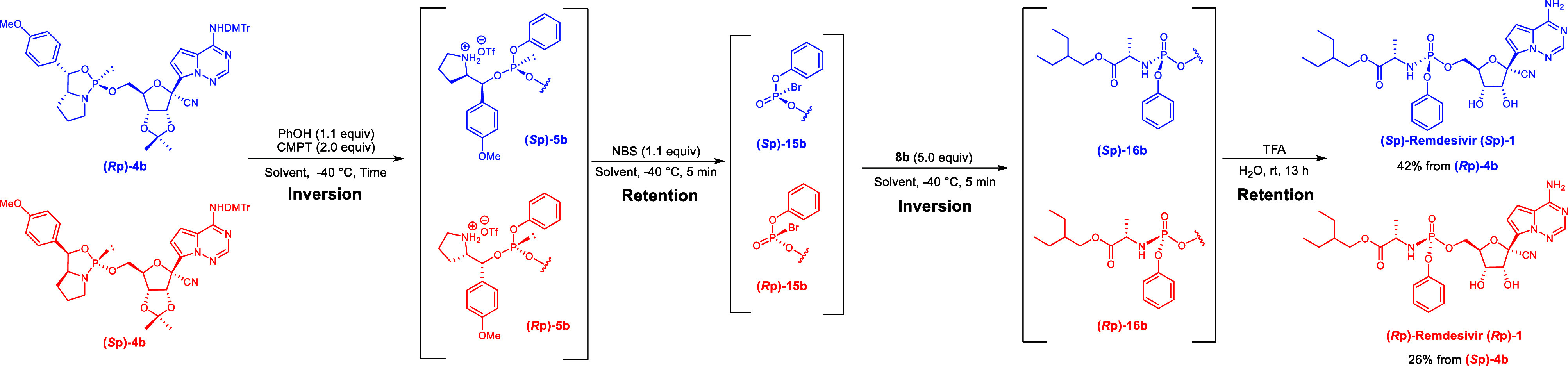
Investigation of the Condensation Conditions of Compound **4b** (Corrected)

Entry^a^	Scale (mmol)	Solvent	Time (min)	^31^P NMR yield of **16b** ^c^ (%)	Isolated yield of **1** (%)	dr
1	0.10	CH_3_CN	10	34	–	>99:1^d^
2	0.10	CH_3_CN-toluene	30	51	–	>99:1^d^
3^b^	0.10	CH_3_CN-toluene	60	87	26	>99:1^e^
4^b^	1.1	CH_3_CN-toluene	60	87	42	>99:1^e^

a **(** ***S*** **p)-4b** and **(** ***R*** **p)-4b** were used in Entries 1–3 and Entry 4, respectively.

b Determined from the ^31^P NMR spectra of crude mixtures.

c Determined from the ^31^P NMR spectra of crude mixtures.

d Extraction in 0.2 M citric acid aqueous solution.

e Determined from the ^31^P NMR spectra after purification.

**Table 3:**
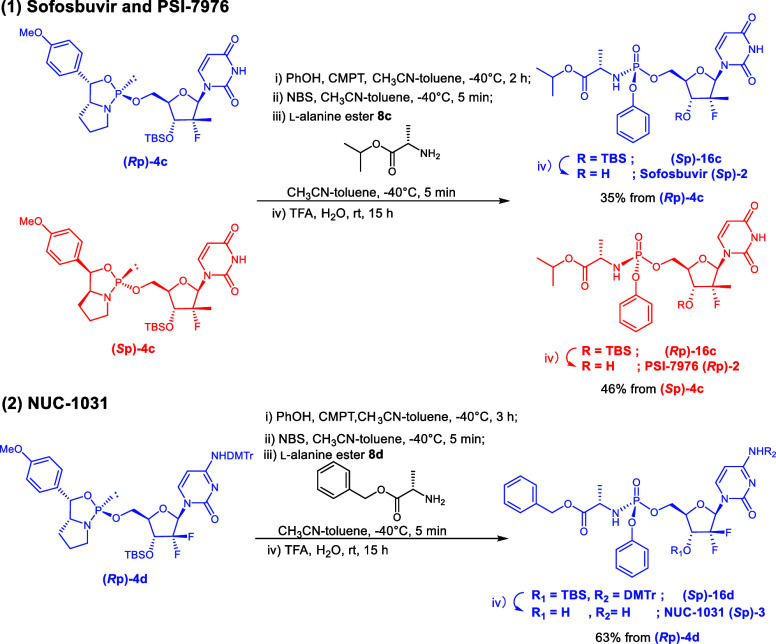
Synthesis Results of Sofosbuvir, PSI-7976, and NUC-1031 (Corrected)

Product	Scale (mmol)	^31^P NMR yield of **16** ^a^ (%)	Isolated yield of product (%)	dr^b^
**(*S*p)-2**	0.16	86	35	>99:1
**(*R*p)-2**	1.0	88	46	>99:1
**(*S*p)-3**	0.10	85	63	>99:1

a Determined from the ^31^P NMR spectra of crude mixtures.

b Determined from the ^31^P NMR spectra after purification.

## Experimental Section

### General Information

Corrected for **13a** and **13b**:

#### 2-Ethylbutyl (*S*)-(((3a*R*,4*R*,6*R*,6a*R*)-6-(6-((bis­(4-methoxyphenyl)­(phenyl)­methyl)­amino)-9H-purin-9-yl)-2,2-dimethyltetrahydrofuro­[3,4-*d*]­[1,3]­dioxol-4-yl)­methoxy)­(phenoxy)­phosphoryl)-l-alaninate (**13a**)

Compound **(** ***R*** **p)-4a** (168.8 mg, 0.20 mmol) was dissolved in toluene (1.4 mL), dried over MS 4A for 24 h, and cooled to −40 °C. A mixture of CMPT (102.5 mg, 0.39 mmol) and PhOH (19.0 mg, 0.20 mmol) in CH_3_CN (0.7 mL), which was dried over MS 3A for 24 h, was added over 1 min to the solution. After stirred for 10 min at −40 °C, NBS (39.5 mg, 0.22 mmol) in CH_3_CN (0.6 mL) was added to the reaction mixture at −40 °C and stirred for 5 min, then 1.0 M CH_3_CN solution of l-alanine ester **8b** (1.0 mL, 1.0 mmol) was added to the reaction mixture and stirred at room temperature for 6 min. The mixture was diluted with CH_2_Cl_2_ (30 mL) and washed with 0.2 M citric acid aqueous solution (30 mL) and saturated NaHCO_3_ aqueous solution (30 mL). The organic layer was dried over Na_2_SO_4_, filtered, and concentrated under reduced pressure to give the crude **13a** (223.7 mg). The residue (168.6 mg) was purified by silica gel column chromatography (neutral silica gel 40–50 μm, 10 g) using toluene–EtOAc (7:3, v/v containing 1% triethyl­amine) as an eluent to give **13a** as a colorless foam (53.5 mg, 37%) (dr >99:1). Please refer to the revised Supporting Information for the spectral data.

#### 2-Ethylbutyl­((*R*)-(((3a*R*,4*R*,6*R*,6a*R*)-6-(6-((bis­(4-methoxyphenyl)­(phenyl)­methyl)­amino)-9H-purin-9-yl)-2,2-dimethyltetrahydrofuro­[3,4-*d*]­[1,3]­dioxol-4-yl)­methoxy)­(phenoxy)­phosphoryl)-l-alaninate (**13b**)

Compound **(** ***S*** **p)-4a** (84.4 mg, 0.10 mmol) was dissolved in CD_3_CN (0.3 mL), dried over MS 3A for 24 h, and cooled to −40 °C. A mixture of CMPT (52.3 mg, 0.20 mmol) and PhOH (9.3 mg, 0.10 mmol) in CD_3_CN (0.3 mL), which was dried over MS 3A for 24 h, was added to the solution. After stirred for 10 min at −40 °C, NBS (19.6 mg, 0.11 mmol) in CD_3_CN (0.1 mL) was added to the reaction mixture at −40 °C and stirred for 5 min, then 3.3 M CD_3_CN solution of l-alanine ester **8b** (0.15 mL, 5.0 mmol) was added to the reaction mixture and stirred at room temperature for 3 h. The mixture was diluted with EtOAc (10 mL) and washed with saturated NaHCO_3_ aqueous solution (3 × 10 mL). The organic layer was dried over Na_2_SO_4_, filtered, and concentrated under reduced pressure. The residue was purified by silica gel column chromatography (neutral silica gel, 40 g) using toluene–EtOAc (8:2–7:3, v/v) as an eluent and preparative TLC (hexane–EtOAc 7:3, v/v) to give **13b** as a colorless foam (23.0 mg, 25%) (dr = 99:1). Please refer to the revised Supporting Information for the spectral data.

### General Procedure of the Synthesis of ProTides

Compound **(** ***R*** **p)-4b** (0.95 g, 1.1 mmol), **(** ***S*** **p)-4b** (88.4 mg, 0.10 mmol), **(** ***R*** **p)-4c** (98.5 mg, 0.16 mmol), **(** ***S*** **p)-4c** (0.61 g, 1.0 mmol), or **(** ***R*** **p)-4d** (90.3 mg, 0.10 mmol) was dissolved in toluene (0.7 mL for the synthesis of **(** ***R*** **p)-1** and **3**; 7.0 mL for the synthesis of **(** ***S*** **p)-1** and **(** ***R*** **p)-2**; and 1.4 mL for the **(** ***S*** **p)-2**), dried over MS 4A for 2 h, and cooled to −40 °C. A mixture of CMPT (0.56 g, 2.2 mmol for **(** ***S*** **p)-1**; 51.1 mg, 0.20 mmol for **(** ***R*** **p)-1**; 86.2 mg, 0.33 mmol for **(** ***S*** **p)-2**; 0.52 g, 2.0 mmol for **(** ***R*** **p)-2**; and 53.5 mg, 0.21 mmol for **3**) and PhOH (0.11 g, 1.2 mmol for **(** ***S*** **p)-1**; 11.2 mg, 0.12 mmol for **(** ***R*** **p)-1**; 16.8 mg, 0.18 mmol for **(** ***S*** **p)-2**; 0.10 g, 1.1 mmol for **(** ***R*** **p)-2**; and 11.3 mg, 0.12 mmol for **3**) in CH_3_CN (3,0 mL for **(** ***S*** **p)-1** and **(** ***R*** **p)-2**; 0.3 mL for **(** ***R*** **p)-1** and **3**; and 0.6 mL for **(** ***S*** **p)-2**), which was dried over MS 3A for 24 h, was added to the solution. After stirred for designated time (1 h for **(** ***S*** **p)-1** and **(** ***R*** **p)-1**; 2 h for **(** ***S*** **p)-2** and **(** ***R*** **p)-2**; and 3 h for **3)** at −40 °C, NBS (194.9 mg, 1.1 mmol for **(** ***S*** **p)-1**; 19.7 mg, 0.11 mmol for **(** ***R*** **p)-1**; 0.45 mg, 0.16 mmol for **(** ***S*** **p)-2**; 0.20 g, 1.1 mmol for **(** ***R*** **p)-2**; and 19.8 mg, 0.11 mmol for **3**) in CH_3_CN (3.0 mL for **(** ***S*** **p)-1** and **(** ***R*** **p)-2**; 0.3 mL for **(** ***R*** **p)-1** and **3**; and 0.6 mL for **(** ***S*** **p)-2**) was added to the reaction mixture at −40 °C and stirred for 5 min, then CH_3_CN solution of l-alanine ester (**8b** (5.0 mL, 5.0 mmol) for **(** ***S*** **p)-1**; **8b** (1.0 mL, 0.05 mmol) for **(** ***R*** **p)-1**; **8c** (1.0 mL, 1.0 mmol) for **(** ***S*** **p)-2**; **8c** (5.0 mL, 5.0 mmol) for **(** ***R*** **p)-2**; and **8d** (0.5 mL, 0.5 mmol) for **3**) was added to the reaction mixture and stirred at room temperature for 5 min.

## Supplementary Material



## Notes

Experimental details, HPLC profiles, copies of ^1^H, ^13^C, and ^31^P NMR spectra of new compounds and final products (corrected) (PDF)

