# Supplementary material for: Stereoselective Synthesis of Nucleotide Analog Prodrugs (ProTides) via an Oxazaphospholidine Method
Source: J Org Chem. 2025 May 8;90(19):6434–42. doi: 10.1021/acs.joc.5c00240 (PMC12090215; doi:10.1021/acs.joc.5c00240)
Supplement: Supplementary file 1 — jo5c00240_si_001.pdf [file jo5c00240_si_001.pdf]

Supporting Information

Stereoselective synthesis of nucleotide analog prodrugs (ProTides) via an oxazaphospholidine method

Monta Nakamura[†], Kiyoshi Kakuta[†], Kazuki Sato[†], Takeshi Wada[†,*]

[†]Department of Medicinal and Life Sciences, Faculty of Pharmaceutical Sciences, Tokyo University of Science, 2641 Yamazaki, Noda, Chiba 278-8510, Japan

Table of Contents

1. General information……………………………………………………………………...S2
2. Experimental section……………………………………………………………………..S3
2.1 Synthesis of protected nucleosides……………………………………………………..S3
2.2 Synthesis of 5'-oxazaphospholidine derivatives…………………………………………S5
3. Additional Information…………………………………………………………………S12
3.1 Reaction monitoring by ^{31}P NMR……………………………………………..………...S12
3.2 Plausible epimerization mechanism……………………………………………………..S15
3.3 Investigation of 3'-*O*-protecting groups of a Sofosbuvir derivative……………………..S16
4. Copies of RP-HPLC profiles and ^{1}H, ^{13}C, and ^{31}P NMR spectra………………………….………S17
4.1 Copies of RP-HPLC profiles……………………………………………………………..S17
4.2 Copies of ^{1}H, ^{13}C, and ^{31}P NMR spectra……………………..…………………………S19
5. References……………………………………………………………..……………………..S68

1. General information

All the reactions were conducted under Ar atmosphere. Dry organic solvents were prepared by the appropriate relevant procedures. The ^{1}H NMR spectra were recorded at 400 or 500 MHz and the internal standard were tetramethylsilane (δ 0.00) or each deuterated solvent signal; $CDCl_3$ (δ 7.26), CD_3CN (δ 1.94), DMSO-d6 (δ 2.50), or CD_3OD (δ 3.31). The ^{13}C NMR spectra were recorded at 101 or 126 MHz and the internal standard were deuterated solvent signal; $CDCl_3$ (δ 77.16), CD_3CN (δ 118.26), CD_3OD (δ 49.00), or DMSO-d6 (δ 39.52). The ^{31}P NMR spectra were recorded at 162 MHz or 202 MHz with 85% H_3PO_4 (δ 0.00) as the external standard in $CDCl_3$, CD_3CN, or DMSO-d6. Hydrogen multiplicity information was obtained by DEPT spectra. IR spectra were obtained using an ATR-IR spectrometer. Analytical TLC was performed on commercial glass plated 0.25 mm thickness silica gel layer. Manual silica gel column chromatography was performed using spherical, neutral, 63–210 µm silica gel unless otherwise noted. Automated silica gel column chromatography was performed on amino silica gel (Yamazen UNIVERSAL Premium column (30 µm)) (Yamazen Corporation) using automated flash chromatography system W-prep 2XY (Yamazen Corporation). The detections in RP-HPLC were achieved at 286 nm at a temperature of 30 °C and a flow rate of 0.5 mL/min using a C18 column (5 µm, 100 Å, 3.9 × 150 mm). GS-441524 and amino acid esters were purchased from Sapala Organics and TCI (Tokyo Chemical Industry Co., Ltd.). 5'-Oxazaphospholidine derivatives were dissolved in toluene and dried by MS 4A for 2–24h. CMPT and PhOH were dissolved in CH_3CN and dried by MS 3A for 24 h.

2. Experimental section

2.1 Synthesis of protected nucleosides

Scheme S1. Synthesis of compound S2

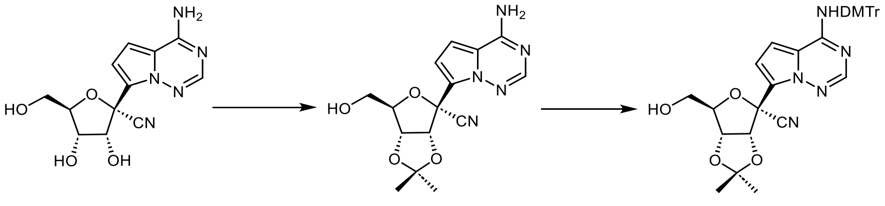

GS-441524 **S1** **S2**

((3aR,4R,6S,6aS)-6-(4-Aminopyrrolo[2,1-f][1,2,4]triazin-7-yl)-2,2-dimethyltetrahydrofuro[3,4-d][1,3]dioxol-4-yl)methanol (S1)[1]:

GS-441524[1] (5.82 g, 20 mmol) was dissolved in acetone (800 mL) and triethyl orthoformate (21 mL, 12.7 mmol) and *p*-toluene sulfonic acid monohydrate (16.75 g, 88 mmol) were successively added to the mixture at rt. After stirred for 3 h, the solution was neutralized with a saturated $NaHCO_3$ aqueous solution (150 mL) and concentrated to a small volume under reduced pressure. The residue was diluted with $CHCl_3$ (150 mL) and washed with saturated $NaHCO_3$ aqueous solutions (3 × 150 mL). The aqueous layers were combined and back-extracted with $CHCl_3$ (150 mL). The organic layers were combined, dried over Na_2SO_4, filtered, and concentrated under reduced pressure to give **S1** as a colorless solid (6.33 g, 96%). The ^{1}H NMR spectrum corresponded with the literature data[1].

((3aR,4R,6S,6aS)-6-(4-((bis(4-methoxyphenyl)(phenyl)methyl)amino)pyrrolo[2,1-f][1,2,4]triazin-7-yl)-2,2-dimethyltetrahydrofuro[3,4-d][1,3]dioxol-4-yl)methanol (S2):

Compound **S1** (6.33 g, 19.1 mmol) was dried by repeated coevaporation with dry pyridine and dissolved in dry pyridine (76.5 mL). TMSCl (5.5 mL, 43 mmol) was added to the reaction mixture at 30 ºC. After stirred at 30 ºC for 6 h, DMTrCl (10.9 g, 32 mmol) was added to the reaction mixture and stirred at 30 ºC for 44 h. After the reaction mixture was cooled to 0 ºC, 5% $NaHCO_3$ aqueous solution–pyridine (1:1, v/v) (100 mL) was added to the reaction mixture, and stirred at rt for 20 min. The reaction mixture was diluted with $CHCl_3$ (200 mL) and the organic layer was separated. Then, the organic layer was washed with saturated $NaHCO_3$ aqueous solution (3 × 100 mL). The aqueous layers were combined, and back-extracted with $CHCl_3$ (150 mL). The organic layers were combined, dried over Na_2SO_4, filtered, and concentrated under reduced pressure. The residue was purified by automated silica gel column chromatography (neutral silica gel, 54 g 2L size) using a linear gradient of EtOAc–hexane (1:9–6:4, v/v) containing a 1.0% triethylamine as an eluent to give **S2** as a colorless foam (10.9 g, 90%).

^{1}H NMR (500 MHz, DMSO-d6) δ 8.73 (s, 1H), 7.36 (d, *J* = 4.6 Hz, 1H), 7.33 (td, *J* = 7.3, 1.3 Hz, 2H) 7.28-7.24 (m, 6H), 7.19-7.15 (tt, *J* = 1.1, 7.0 1H), 6.92 (d, J = 4.6 Hz, 1H), 6.84 (dt, J = 3.9, 2.54 Hz, 4H), 5.32 (d, J = 6.6 Hz, 1H), 5.00 (t, J = 5.6 Hz, 1H), 4.84 (dd, J = 6.6, 3.1 Hz, 1H), 4.29 (td, J = 5.2, 3.2 Hz, 1H), 3.71 (s, 6H), 3.57-3.45 (m, 2H), 1.61 (s, 3H), 1.34 (s, 3H); **^{13}C NMR** (126 MHz, DMSO-d6) δ 157.5, 153.3, 146.2, 144.8, 136.7, 136.6, 129.9, 128.4, 127.4, 126.1, 122.4, 117.4, 116.1, 115.3, 112.7, 110.4, 101.3, 85.4, 83.9, 81.4, 79.8, 79.1, 69.9, 60.8, 54.9, 25.8, 25.0; **IR** (neat, cm^{-1}) 3424, 2953, 1601, 1508, 1464, 1178, 1031, 827, 725, 580; **HRMS (ESI/QTOF)** $[M + H]^+$ Calcd for $C_{36}H_{36}N_5O_6$ 634.2660; Found 634.2663.

Scheme S2. Synthesis of compound S4

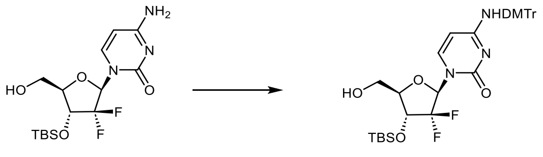

6-*N*-(4,4'-Dimethoxytrityl)-3'-*O-tert*-Buthyldimethylsilyl-2'-deoxy-2'-α-fluoro-2β-C-methyl uridine (S4):

Compound **S3**[2] (1.26 g, 3.3 mmol) was dried by repeated coevaporation with dry pyridine and dissolved in dry pyridine (13.2 mL). TMSCl (0.93 mL, 7.3 mmol) was added to the reaction mixture and stirred at room temperature for 4 h. DMTrCl (1.34 g, 3.9 mmol) was added to the reaction mixture and stirred for 16 h, then quenched with MeOH (5 mL) and saturated $NaHCO_3$ aqueous solution (30 mL). The reaction mixture was extracted with AcOEt (3 × 30 mL) and the organic layers were combined, dried over $MgSO_4$, filtered, and concentrated under reduced pressure. The residue was purified by automated silica gel column chromatography (neutral silica gel, 40 g L size) using a linear gradient of EtOAc–hexane (4:6–13:7, v/v) containing a 1.0% triethylamine as an eluent to give **S4** as a colorless foam (1.83 g, 65%).

^{1}H NMR (500 MHz, DMSO-d6) δ 8.55 (s, 1H), 7.57 (d, J = 7.6 Hz, 1H), 7.28-7.25 (t, J = 7.4 2H), 7.21-7.16 (m, 3H), 7.13-7.22 (d, J = 8.8 Hz, 4H), 6.83 (d, J = 8.8 Hz, 4H), 6.30 (d, J = 7.6 Hz, 1H), 5.99 (t, J = 8.2 Hz, 1H), 5.18 (t, J = 4.9 Hz, 1H), 4.31 (q, J = 10.6 Hz, 1H), 3.72 (s, 6H), 3.57-3.52 (m, 1H), 0.86 (s, 9H), 0.09 (d, J = 8.0 Hz, 6H); **^{13}C NMR** (126 MHz, DMSO-d6) δ 163.3, 157.4, 153.4, 144.8, 139.5, 136.7, 129.8, 128.4, 127.4, 126.1, 112.7, 96.9, 83.5, 79.1, 70.1 (t, J = 22.3 Hz), 69.5, 58.7, 54.9, 25.3, 17.6, -5.0, -5.1; **IR** (neat, cm^{-1}) 2930, 1650, 1493, 1409, 1250, 1206, 1178, 1080, 1032, 835, 754, 701, 582, 460; **HRMS (ESI/QTOF)** $[M + H]^+$ Calcd for $C_{36}H_{44}F_2N_3O_6Si$ 680.2967; Found 680.2963.

2.2 Synthesis of 5'-oxazaphospholidine derivatives

Scheme S3. Synthesis of phosphitylation reagent

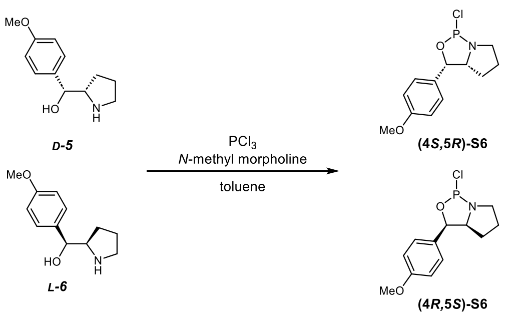

Compound (4*S*,5*R*)-S6 and **(4*R*,5*S*)-S6**[3]**:**

These compounds were synthesized according to the method of reference 3. These compounds were used for phosphitylation without purification.

Scheme S4. Synthesis of 5'-oxazaphospholidine derivatives

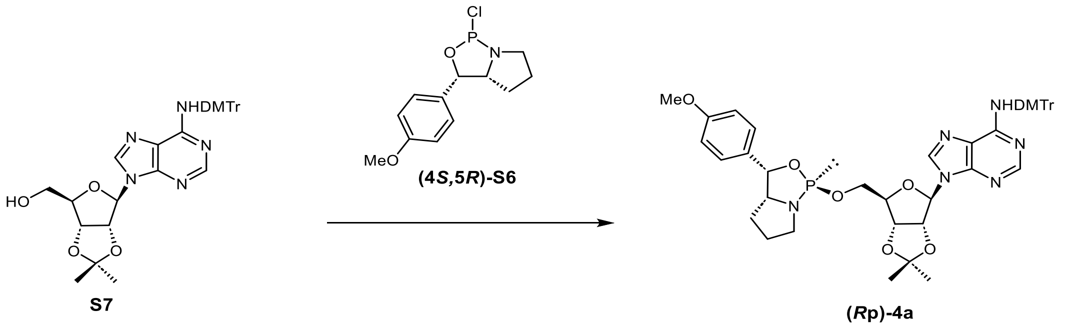

***N*-(Bis(4-methoxyphenyl)(phenyl)methyl)-9-((3a*R*,4*R*,6*R*,6a*R*)-6-((((1*R*,3*S*,3a*R*)-3-(4-methoxyphenyl)tetrahydro-1H,3H-pyrrolo[1,2-c][1,3,2]oxazaphosphol-1-yl)oxy)methyl)-2,2-dimethyltetrahydrofuro[3,4-d][1,3]dioxol-4-yl)-9H-purin-6-amine (*R*p)-4a**:

Compound **S7**[4] (1.83 g, 3.0 mmol) was dried by repeated coevaporation with dry pyridine, dry toluene, and dry THF, and then dissolved in THF (15 mL). Triethylamine (2.92 mL, 21 mmol) was added to the mixture. After the mixture was cooled to −78 °C, a 1.25 M THF solution of **(4*S*,5*R*)-S6** (6.0 mL, 7.5 mmol) was added dropwise over 20 min. Then, the mixture was warmed to rt and stirred for 1 h. The mixture was cooled to 0 °C and diluted with $CHCl_3$ (200 mL). The mixture was washed with saturated $NaHCO_3$ aqueous solutions (3 × 50 mL) and combined aqueous solution was back-extracted with $CHCl_3$ (150 mL). The organic layers were combined, dried over Na_2SO_4, filtered, and concentrated under reduced pressure. The residue was purified by manual silica gel column chromatography (amino silica gel, 80 g, toluene–EtOAc (9:1, v/v) containing a 0.1% triethylamine) as an eluent to give **(*R*p)-4a** as a colorless foam (0.88 g, 35%).

^{1}H NMR (500 MHz, $CDCl_3$) δ 8.07 (s, 1H), 8.03 (s, 1H), 7.33–7.29 (m, 2H), 7.22 (dt, *J* = 8.8, 1.9 Hz, 4H), 7.15 (tt, *J* = 9.1, 2.3 Hz, 3H), 6.87–6.82 (m, 4H), 6.80–6.75 (m, 4H), 6.12 (d, *J* = 2.6 Hz), 5.67 (d, *J* = 6.3 Hz, 1H), 5.32 (dd, *J* = 6.2, 2.6 Hz, 1H), 5.00 (dd, *J* = 6.2, 2.6 Hz, 1H), 4.47–4.45 (m, 1H), 4.01–3.97 (m, 2H), 3.82–3.72 (m, 10H), 3.59–3.51 (m, 1H), 3.15–3.09 (m, 1H), 1.61–1.53 (m, 5H), 1.35 (s, 3H), 1.23–1.17 (m, 1H), 1.02–0.94 (m, 1H); **^{13}C{^{1}H} NMR** (126 MHz, $CDCl_3$) δ 159.1, 158.4, 154.2, 152.6, 148.7, 145.6, 139.0, 137.6, 130.2 (d, $^3J_{PC}$ = 3.8 Hz), 128.9, 128.9, 128.0, 126.9, 126.8, 121.3, 114.4, 114.3, 114.2, 113.9, 113.7, 113.3, 90.9, 86.0 (d, $^2J_{PC}$ = 5.2 Hz), 84.5, 82.8 (d, $^2J_{PC}$ = 9.8 Hz), 81.9, 81.9, 70.8, 70.7, 67.7 (d, $^2J_{PC}$ = 3.1 Hz), 63.5 (d, $^2J_{PC}$ = 9.4 Hz), 55.4, 55.3,

55.3, 47.3 (d, $^{2}J_{PC}$ = 34.8 Hz), 28.1, 27.3, 26.1 (d, $^{3}J_{PC}$ = 3.1 Hz), 25.4; **^{31}P{^{1}H} NMR** (202 MHz, CDCl3) δ 153.96; **IR** (neat, cm^{-1}) 1601, 1508, 1464, 1177, 1031, 827, 726, 702, 581, 427, 411; **HRMS (ESI/QTOF)** $[M + H]^+$ Calcd for $C_{46}H_{50}N_6O_8P$ 845.3422; Found 845.3424.

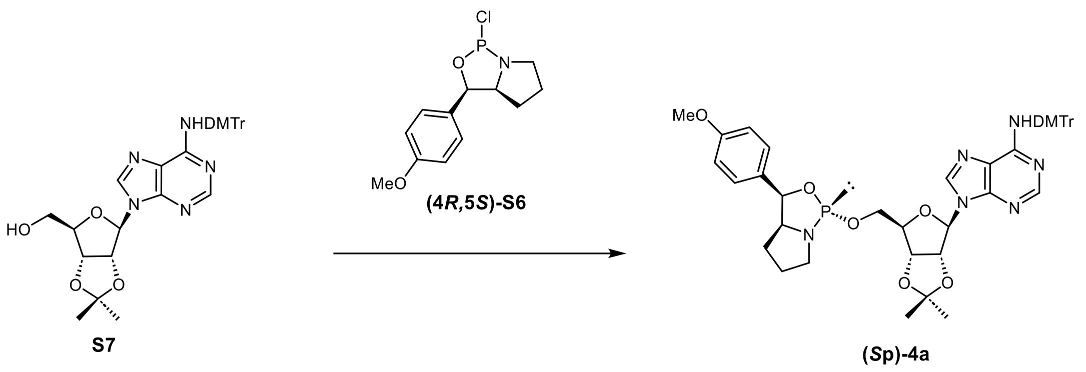

***N*-(Bis(4-methoxyphenyl)(phenyl)methyl)-9-((3a*R*,4*R*,6*R*,6a*R*)-6-((((1*S*,3*S*,3a*S*)-3-(4-methoxyphenyl)tetrahydro-1H,3H-pyrrolo[1,2-c][1,3,2]oxazaphosphol-1-yl)oxy)methyl)-2,2-dimethyltetrahydrofuro[3,4-d][1,3]dioxol-4-yl)-9H-purin-6-amine (*S*p)-4a**:

Compound **S7**[4] (1.37 g, 2.25 mmol) was dried by repeated coevaporation with dry pyridine, dry toluene, and dry THF, and then dissolved in THF (10 mL). Triethylamine (1.95 mL, 14 mmol) was added to the mixture. After the mixture was cooled to −78 °C, a 1.25 M THF solution of **(4*R*,5*S*)-S6** (4.0 mL, 5.0 mmol) was added dropwise over 4 min. Then, the mixture was warmed to rt and stirred for 1 h. The mixture was cooled to 0 °C and diluted with $CHCl_3$ (200 mL). The mixture was washed with saturated $NaHCO_3$ aqueous solutions (3 × 100 mL) and combined aqueous solution was back-extracted with $CHCl_3$ (100 mL). The organic layers were combined, dried over Na_2SO_4, filtered, and concentrated under reduced pressure. The residue was purified by manual silica gel column chromatography (diol silica gel, 100 g, hexane–EtOAc (6:4, v/v) containing a 4% triethylamine) as an eluent to give **(*S*p)-4a** as a colorless foam (0.89 g, 46%).

^{1}H NMR (400 MHz, $CDCl_3$) δ 8.11 (s, 1H), 8.08 (s, 1H), 7.33 (dt, J = 8.4, 1.9 Hz, 2H), 7.25–7.17 (m, 8H), 6.88–6.82 (m, 3H), 6.81–6.77 (m, 4H), 6.15 (d, J = 3.2 Hz, 1H), 5.67 (d, J = 6.4 Hz, 1H), 5.30 (dd, J = 6.4, 3.2 Hz, 1H), 4.98 (dd, J = 6.4 2.7 Hz, 1H), 4.47 (q, J = 3.5 Hz, 1H), 4.07–4.01 (m, 1H), 3.98–3.92 (m, 1H), 3.8–3.76 (m, 10H), 3.61–3.52 (m, 1H), 3.22–3.10 (m, 1H), 1.71–1.58 (m, 5H), 1.35 (s, 3H), 1.24–1.16 (m, 1H), 1.04–0.94 (m, 1H); **^{13}C{^{1}H} NMR** (101 MHz, $CDCl_3$) δ 159.0, 158.3, 154.2, 152.7, 148.7, 145.6, 139.0, 137.6, 130.2, 128.9, 128.0, 126.8, 121.3, 113.8, 113.3, 90.9, 86.0 (d, $^{3}J_{PC}$ = 4.8 Hz), 84.5, 82.8 (d, $^{2}J_{PC}$ = 10.6 Hz), 81.9, 70.8, 67.7 (d, $^{2}J_{PC}$ = 2.9 Hz), 63.5 (d, $^{2}J_{PC}$ = 8.7 Hz), 55.4, 47.3 (d, $^{2}J_{PC}$ = 34.7 Hz), 28.1, 27.3, 26.1 (d, $^{3}J_{PC}$ = 2.9 Hz), 25.4; **^{31}P{^{1}H} NMR** (162 MHz, $CDCl_3$) δ 154.60; **IR** (neat, cm^{-1}) 1602, 1509, 1467, 1452, 1375, 1294, 1246, 1173, 1030, 823, 794, 750, 700, 645, 588, 424; **HRMS (ESI/QTOF)** $[M + H]^+$ Calcd for $C_{46}H_{50}N_6O_8P$ 845.3422; Found 845.3433.

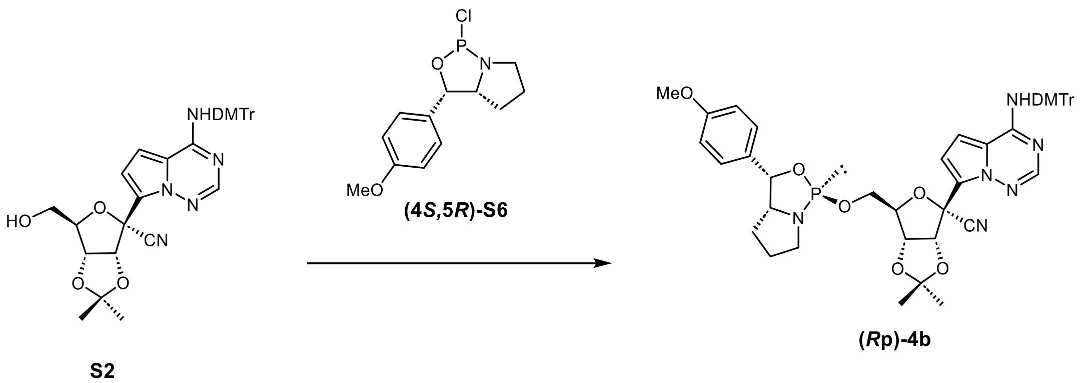

(3a*R*,4*R*,6*R*,6a*R*)-4-(4-((Bis(4-methoxyphenyl)(phenyl)methyl)amino)pyrrolo[2,1-f][1,2,4]triazin-7-yl)-6-((((1*R*,3*S*,3a*R*)-3-(4-methoxyphenyl)tetrahydro-1H,3H-pyrrolo[1,2-c][1,3,2]oxazaphosphol-1-yl)oxy)methyl)-2,2-dimethyltetrahydrofuro[3,4-d][1,3]dioxole-4-carbonitrile (*R*p)-4b:

Compound **S2** (1.26 g, 2.0 mmol) was dried by repeated coevaporation with dry pyridine, dry toluene, and dry THF, and then dissolved in THF (10 mL). Triethylamine (1.95 mL, 14 mmol) was added to the mixture. After the mixture was cooled to −78 °C, a 1.25 M THF solution of **(4*S*,5*R*)-S6** (4.0 mL, 5.0 mmol) was added dropwise over 10 min. Then, the mixture was warmed to rt and stirred for 2.5 h. The mixture was cooled to 0 °C and diluted with $CHCl_3$ (50 mL). The mixture was washed with saturated $NaHCO_3$ aqueous solutions (3 × 50 mL) and combined aqueous solution was back-extracted with $CHCl_3$ (50 mL). The organic layers were combined, dried over Na_2SO_4, filtered, and concentrated under reduced pressure. The residue was purified by manual silica gel column chromatography (amino silica gel, 90 g, toluene–EtOAc (17:3, v/v) containing a 1% triethylamine) as an eluent to give **(*R*p)-4b** as a colorless foam (0.49 g, 28%).

^{1}H NMR (500 MHz, CD_3CN) δ 7.54 (s, 1H), 7.36 (dt, J = 7.7, 1.6 Hz, 3H), 7.27 (dt, J = 8.9, 1.9 Hz, 6H), 7.21–7.08 (m, 4H), 6.94 (d, J = 4.6 Hz, 1H), 6.90–6.85 (m, 2H), 6.81 (dt, J = 8.9, 2.1 Hz, 4H), 5.60 (d, J = 6.4 Hz, 1H), 5.29 (d, J = 6.4 Hz, 1H), 4.87 (dd, J = 6.4, 3.1 Hz, 1H), 4.48–4.45 (m, 1H), 3.96–3.92 (m, 1H), 3.86–3.73 (m, 11H), 3.48–3.41 (m, 1H), 2.98–2.86 (m, 1H), 1.63 (s, 3H), 1.59–1.45 (m, 2H), 1.34 (s, 3H), 1.15–1.09 (m, 1H), 0.92–0.85 (m, 1H); **^{13}C{^{1}H} NMR** (126 MHz, CD_3CN) δ 160.0, 159.3, 154.4 147.0, 145.8, 137.9, 137.7, 131.7 (d, $^3J_{PC}$ = 3.8 Hz), 131.1, 129.6, 128.6, 128.0, 127.8, 127.6, 124.2, 116.6, 114.4, 113.8, 111.8, 101.1, 85.6 (d, $^3J_{PC}$ = 4.5 Hz), 85.4, 84.1 (d, $^2J_{PC}$ = 10.0 Hz), 83.2, 81.8, 71.6, 68.2 (d, $^3J_{PC}$ = 3.0 Hz), 63.0 (d, $^2J_{PC}$ = 4.7 Hz), 55.8, 55.8, 47.5 (d, $^2J_{PC}$ = 35.0 Hz), 28.7, 27.2, 26.6 (d, $^3J_{PC}$ = 3.2 Hz), 26.5, 25.4; **^{31}P{^{1}H} NMR** (202 MHz, CD_3CN) δ 151.45; **IR** (neat, cm^{-1}) 2936, 1756, 1601, 1508, 1458, 1370, 1296, 1247, 1215, 1081, 1029, 950, 823, 790, 579, 467, 415; **HRMS (ESI/QTOF) *m/z*:** [M + Na] Calcd for $C_{48}H_{49}N_6NaO_8P$ 891.3247; Found 891.3243.

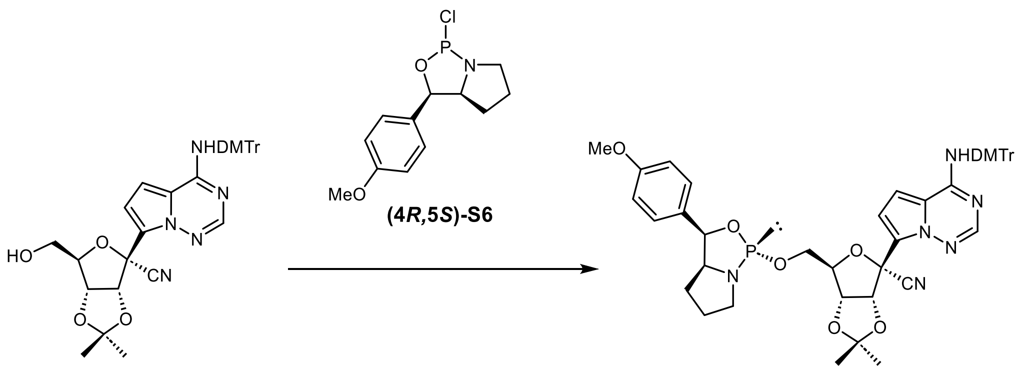

S2 **(*S*p)-4b**

(3a*R*,4*R*,6*R*,6a*R*)-4-(4-((Bis(4-methoxyphenyl)(phenyl)methyl)amino)pyrrolo[2,1-f][1,2,4]triazin-7-yl)-6-((((1*S*,3*R*,3a*S*)-3-(4-methoxyphenyl)tetrahydro-1H,3H-pyrrolo[1,2-c][1,3,2]oxazaphosphol-1-yl)oxy)methyl)-2,2-dimethyltetrahydrofuro[3,4-d][1,3]dioxole-4-carbonitrile (*S*p)-4b:

Compound **S2** (0.63 g, 1,0 mmol) was dried by repeated coevaporation with dry pyridine, dry toluene, and dry THF, and then dissolved in THF (5.0 mL). Triethylamine (0.98 mL, 7.0 mmol) was added to the mixture. After the mixture was cooled to −78 °C, a 1.25 M THF solution of **(4*R*,5*S*)-S6** (2.0 mL, 2.5 mmol) was added dropwise over 2 min. Then, the mixture was warmed to rt and stirred for 1 h. The mixture was cooled to 0 °C and diluted with $CHCl_3$ (100 mL). The mixture was washed with saturated $NaHCO_3$ aqueous solutions (3 × 50 mL) and combined aqueous solution was back-extracted with $CHCl_3$ (50 mL). The organic layers were combined, dried over Na_2SO_4, filtered, and concentrated under reduced pressure. The residue was purified by manual silica gel column chromatography (diol silica gel, 100 g, hexane–EtOAc (11:9, v/v) containing a 4% triethylamine) as an eluent to give **(*S*p)-4b** as a colorless foam (0.41 g, 46%).

^{1}H NMR (500 MHz, $CDCl_3$) δ 7.74 (s, 1H), 7.29–7.26 (m, 3H), 7.20–7.13 (m, 7H), 6.94 (d, J = 4.6 Hz, 1H), 6.85 (dt, J = 8.7, 2.1 Hz, 2H), 6.82 (dt, J = 8.9, 2.9 Hz, 5H), 6.51 (d, J = 4.4 Hz, 1H), 6.38 (s, 1H), 5.71 (d, J = 6.3 Hz, 1H), 5.41 (d, J = 6.8 Hz, 1H), 4.94 (dd, J = 6.8, 3.7 Hz, 1H), 4.56 (q, I = 4.5 Hz, 1H), 4.09–3.97 (m, 2H), 3.86–3.81 (m, 1H), 3.79 (s, 6H), 3.79 (s, 3H), 3.58–3.50 (m, 1H), 3.17–3.11 (m, 1H), 1.73 (s, 3H), 1.63–1.57 (m, 2H), 1.31 (s, 3H), 1.22–1.16 (m, 1H), 1.02–0.94 (m, 1H); **^{13}C{^{1}H} NMR** (126 MHz, $CDCl_3$) δ 159.1, 158.6, 153.5, 147.0, 144.9, 136.9,130.6 (d, $^3J_{PC}$ = 3.8 Hz), 130.0, 128.7, 128.2, 127.3, 127.2, 126.9, 123.1, 117.9, 117.0, 116.1, 113.9, 113.7, 113.5, 112.1, 84.4 (d, $^2J_{PC}$ = 3.9 Hz), 84.1, 82.7 (d, $^3J_{PC}$ = 9.7 Hz), 82.3, 81.2, 71.2, 67.7 (d, $^2J_{PC}$ = 2.9 Hz), 62.6 (d, $^2J_{PC}$ = 8.9 Hz), 55.4, 55.4, 47.3 (d, $^2J_{PC}$ = 34.7 Hz), 28.2, 26.4, 25.1 (d, $^3J_{PC}$ = 3.4 Hz), 25.5; **^{31}P{^{1}H} NMR** (202 MHz, $CDCl_3$) δ 153.76; **IR** (neat, cm^{-1}) 2936, 1755, 1603, 1510, 1460, 1298, 1247, 1171, 1024, 831, 792, 726, 577, 445, 422; **HRMS (ESI/QTOF)** $[M + H]^+$ Calcd for $C_{48}H_{50}N_6O_8P$ 869.3422; Found 869.3429.

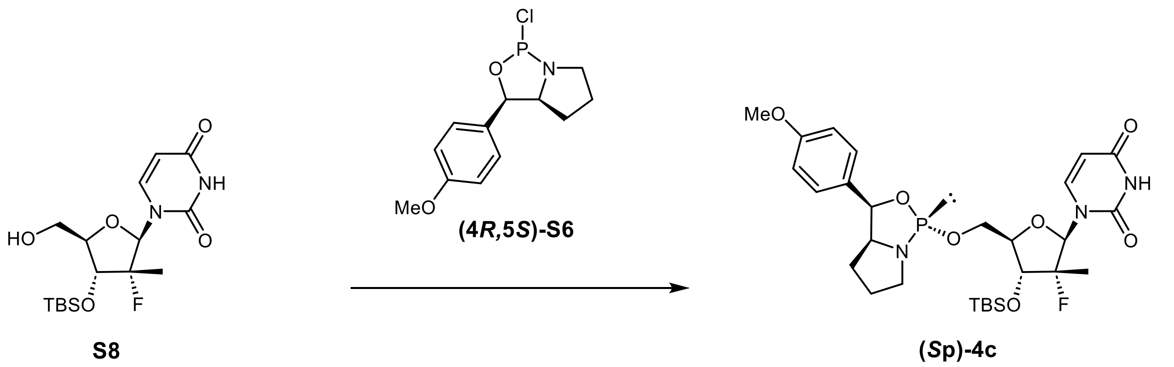

1-((2*R*,3*R*,4*R*,5*R*)-4-((*tert*-Butyldimethylsilyl)oxy)-3-fluoro-5-((((1*S*,3*R*,3a*S*)-3-(4-methoxyphenyl)tetrahydro-1H,3H-pyrrolo[1,2-c][1,3,2]oxazaphosphol-1-yl)oxy)methyl)-3-methyltetrahydrofuran-2-yl)pyrimidine-2,4(1H,3H)-dione (*S*p)-4c:

Compound **S8**[5] (0.64 g, 1.7 mmol) was dried by repeated coevaporation with dry pyridine, dry toluene, and dry THF, and then dissolved in THF (8.5 mL). Triethylamine (1.65 mL, 11.9 mmol) was added to the mixture. After the mixture was cooled to −78 °C, a 1.25 M THF solution of **(4*R*,5*S*)-S6** (3.4 mL, 4.3 mmol) was added dropwise over 3 min. Then, the mixture was warmed to rt and stirred for 1 h. The mixture was cooled to 0 °C and diluted with $CHCl_3$ (200 mL). The mixture was washed with saturated $NaHCO_3$ aqueous solutions (3 × 50 mL) and combined aqueous solution was back-extracted with $CHCl_3$ (2 × 50 mL). The organic layers were combined, dried over Na_2SO_4, filtered, and concentrated under reduced pressure. The residue was purified by manual silica gel column chromatography (amino silica gel, 70 g, toluene–EtOAc (1:9, v/v) containing a 1% triethylamine) as an eluent to give **(*S*p)-4c** as a colorless foam (0.42 g, 46%).

^{1}H NMR (500 MHz, $CDCl_3$) δ 8.31 (brs, 1H), 8.11 (d, J = 8.1 Hz, 1H), 7.20 (dt, J = 8.4, 2.8 Hz, 2H), 6.90 (dt, J = 8.7, 2.9 Hz, 2H), 6.20 (d, J = 17.5 Hz, 1H), 5.71–5.68 (m, 2H), 4.26 (ddd, J = 12.1, 5.5, 1.9 Hz, 1H), 4.09 (dd, J = 9.0, 1.3 Hz, 1H), 4.00–3.85 (m, 3H), 3.82 (s, 3H), 3.64–3.56 (m, 1H), 3.21–3.14 (m, 1H), 1.67–1.60 (m, 2H), 1.34 (d, J = 21.8 Hz, 3H), 1.29–1.23 (m, 1H), 1.08–1.00 (m, 1H), 0.93 (s, 9H), 0.18 (s, 3H), 0.15 (s, 3H); **^{13}C{^{1}H} NMR** (126 MHz, $CDCl_3$) δ 162.7, 159.3 150.3, 140.2, 130.1 (d, $^3J_{PC}$ = 3.7 Hz), 126.8, 113.9, 102.6, 100.0 (d, J = 185.9 Hz), 89.1 (d, J = 40.0 Hz), 83.4 (d, $^2J_{PC}$ = 10.0 Hz), 81.0 (d, $^3J_{PC}$ = 6.6 Hz), 72.1 (d, J = 17.2), 67.9 (d, $^2J_{PC}$ = 3.4 Hz), 59.9 (d, $^2J_{PC}$ = 11.1 Hz), 55.4, 47.5 (d, $^2J_{PC}$ = 35.3 Hz), 28.2, 26.2 (d, $^3J_{PC}$ = 3.1 Hz), 25.8, 18.1, 17.0 (d, J = 25.7 Hz), -4.1, -4.3; **^{31}P{^{1}H} NMR** (202 MHz, $CDCl_3$) δ 153.84; **IR** (neat, cm^{-1}) 1684, 1612, 1587, 1511, 1267, 1449, 1370, 1248, 1096, 1024, 965, 926, 838, 761, 560, 447; **HRMS (ESI/QTOF)** [M + H]$^+$ Calcd for $C_{28}H_{42}FN_3O_7PSi$ 610.2508; Found 610.2508.

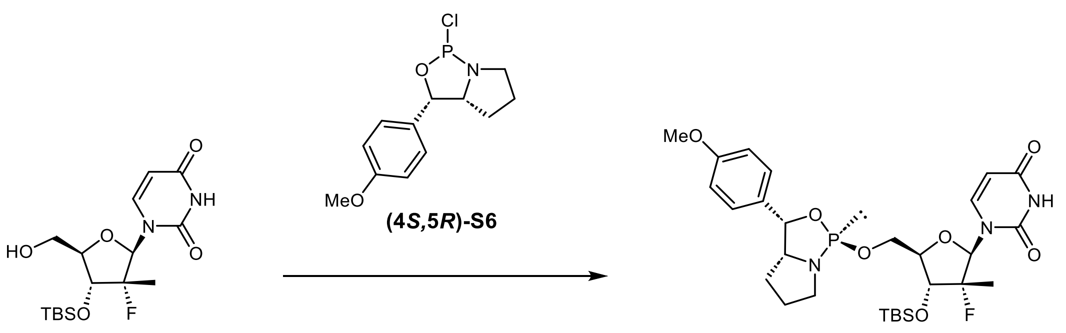

S8 **(Rp)-4c**

1-((2*R*,3*R*,4*R*,5*R*)-4-((*tert*-Butyldimethylsilyl)oxy)-3-fluoro-5-((((1*R*,3*S*,3a*R*)-3-(4-methoxyphenyl)tetrahydro-1H,3H-pyrrolo[1,2-c][1,3,2]oxazaphosphol-1-yl)oxy)methyl)-3-methyltetrahydrofuran-2-yl)pyrimidine-2,4(1H,3H)-dione (*R*p)-4c:

Compound **S8**[5] (0.72 g, 1.9 mmol) was dried by repeated coevaporation with dry pyridine, dry toluene, and dry THF, and then dissolved in THF (10 mL). Triethylamine (1.95 mL, 14 mmol) was added to the mixture. After the mixture was cooled to −78 °C, a 1.25 M THF solution of **(4*S*,5*R*)-S6** (4.0 mL, 5.0 mmol) was added dropwise over 7 min. Then, the mixture was warmed to rt and stirred for 1.5 h. The mixture was cooled to 0 °C and diluted with $CHCl_3$ (100 mL). The mixture was washed with saturated $NaHCO_3$ aqueous solutions (3 × 100 mL) and combined aqueous solution was back-extracted with $CHCl_3$ (100 mL). The organic layers were combined, dried over Na_2SO_4, filtered, and concentrated under reduced pressure. The residue was purified by manual silica gel column chromatography (amino silica gel, 100 g, toluene–EtOAc (1:9, v/v) containing a 1% triethylamine) as an eluent to give **(*R*p)-4c** as a colorless foam (0.65 g, 54%).

^{1}H NMR (500 MHz, $CDCl_3$) δ 8.46 (brs, 1H), 8.10 (d, J = 8.1 Hz, 1H), 7.20 (dt, *J* = 8.4, 2.8 Hz, 2H), 6.90 (dt, *J* = 8.4, 2.8 Hz, 2H), 6.20 (d, *J* = 17.5 Hz, 1H), 5.74 (d, *J* = 6.3 Hz, 1H), 5.68 (d, *J* = 8.1 Hz, 1H), 4.29 (ddd, *J* = 12.0, 5.5, 1.9 Hz, 1H), 4.10 (dd, *J* = 8.9, 1.3 Hz, 1H), 3.99–3.86 (m, 3H), 3.82 (s, 3H), 3.67–3.58 (m, 1H), 3.19–3.12 (m, 1H), 1.72–1.58 (m, 2H), 1.36 (s, 3H), 1.32 (s, 1H), 1.30–1.24 (m, 1H), 1.09–1.01 (m, 1H), 0.91 (s, 9H), 0.14 (s, 3H), 0.12 (s, 3H); **^{13}C{^{1}H} NMR** (126 MHz, $CDCl_3$) δ 162.8, 159.3, 150.3, 140.1, 130.2 (d, $^3J_{PC}$ = 3.7 Hz), 126.8, 113.9, 102.6, 99.8 (d, *J* =185 Hz), 88.9 (d, *J* = 40.2 Hz), 83.2 (d, $^2J_{PC}$ = 9.9 Hz), 80.8 (d, $^3J_{PC}$ = 6.5 Hz), 71.9 (d, *J* = 17.1 Hz), 67.7 (d, $^2J_{PC}$ = 3.4 Hz), 59.7 (d, $^2J_{PC}$ = 11.1 Hz), 55.2, 47.3 (d, $^2J_{PC}$ = 35.3 Hz), 28.0, 26.0 (d, $^3J_{PC}$ = 3.1 Hz), 25.6, 17.9, 16.8 (d, *J* = 25.7 Hz), -4.2, -4.4; **^{31}P{^{1}H} NMR** (202 MHz, $CDCl_3$) δ 154.83; **IR** (neat, cm^{-1}) 2929, 1694, 1514, 1454, 1388, 1248, 1160, 1097, 1035, 923, 835, 608, 567, 546, 426; **HRMS (ESI/QTOF)** [M + H]$^+$ Calcd for $C_{28}H_{42}FN_3O_7PSi$ 610.2508; Found 610.2510.

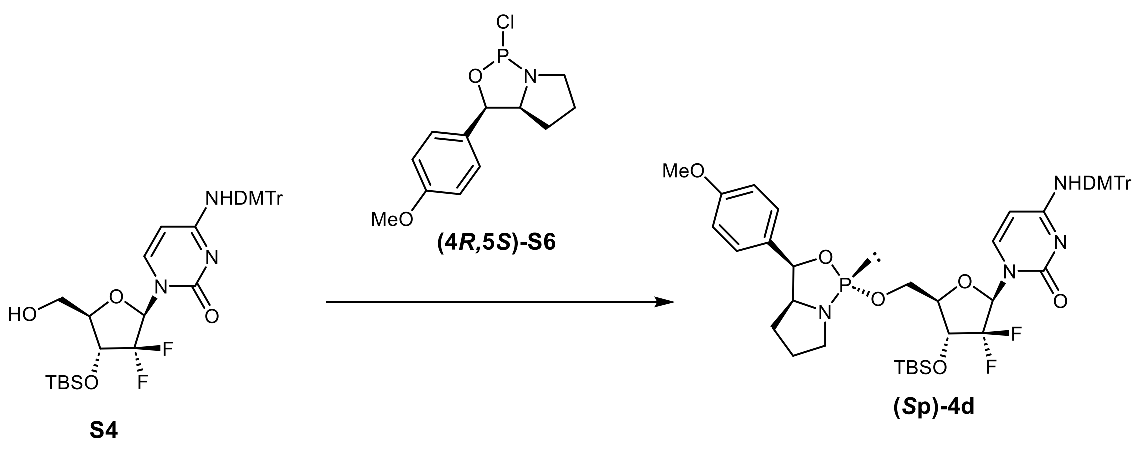

4-((Bis(4-methoxyphenyl)(phenyl)methyl)amino)-1-((2*R*,4*R*,5*R*)-4-((tert-butyldimethylsilyl)oxy)-3,3-difluoro-5-((((1*R*,3*S*,3a*R*)-3-(4-methoxyphenyl)tetrahydro-1H,3H-pyrrolo[1,2-c][1,3,2]oxazaphosphol-1-yl)oxy)methyl)tetrahydrofuran-2-yl)pyrimidin-2(1H)-one (*S*p)-4d:

Compound **S4**[2] (1.14 g, 1.7 mmol) was dried by repeated coevaporation with dry pyridine, dry toluene, and dry THF, and then dissolved in THF (10 mL). Triethylamine (1.95 mL, 14 mmol) was added to the mixture. After the mixture was cooled to −78 °C, a 1.25 M THF solution of **(4*R*,5*S*)-S6** (4.0 mL, 5.0 mmol) was added dropwise over 5 min. Then, the mixture was warmed to rt and stirred for 1 h. The mixture was cooled to 0 °C and diluted with $CHCl_3$ (200 mL). The mixture was washed with saturated $NaHCO_3$ aqueous solutions (3 × 50 mL) and combined aqueous solution was back-extracted with $CHCl_3$ (2 × 50 mL). The organic layers were combined, dried over Na_2SO_4, filtered, and concentrated under reduced pressure. The residue was purified by manual silica gel column chromatography (amino silica gel, 70 g, toluene–EtOAc (8:2, v/v) containing a 1% triethylamine) as an eluent to give **(*S*p)-4d** as a colorless foam (1.20 g, 79%).

^{1}H NMR (500 MHz, $CDCl_3$) δ 7.48 (d, *J* = 7.7 Hz, 1H), 7.31–7.27 (m, 2H), 7.21–7.17 (m, 4H), 7.10 (dt, *J* = 8.7, 3.0 Hz, 4H), 6.87 (dt, *J* = 8.7, 2.9 Hz, 2H), 6.82 (dt, *J* = 8.7, 3.0 Hz, 5H), 6.33 (d, *J* = 13.2 Hz, 1H), 5.81 (d, *J* = 6.4 Hz, 1H), 5.01 (d, *J* = 7.7 Hz, 1H), 4.58–4.50 (m, 1H), 4.01 (dt, *J* = 8.4, 1.9 Hz, 1H), 3.95–3.87 (m, 2H), 3.83–3.77 (m, 10H), 3.62–3.54 (m, 1H), 3.28–3.21 (m, 1H), 1.71–1.65 (m, 2H), 1.26–1.20 (m, 1H), 1.04–0.96 (m, 1H), 0.77 (s, 9H), 0.02 (s, 3H), -0.04 (s, 3H); **^{13}C{^{1}H} NMR** (101 MHz, $CDCl_3$) δ 165.6, 159.2, 158.8, 155.0, 144.7, 140.5, 136.4, 129.9, 128.5 (d, $^3J_{PC}$ = 13.5 Hz), 127.6, 127.0, 113.8, 113.7, 95.1, 81.8 (d, $^2J_{PC}$ = 8.7 Hz), 79.9 (t, $^3J_{PC}$ = 5.8 Hz), 70.4, 67.4 (d, $^2J_{PC}$ = 2.9 Hz), 59.9, 55.4, 55.4, 47.2 (d, $^2J_{PC}$ = 33.7 Hz), 28.2, 26.2 (d, $^3J_{PC}$ = 2.9 Hz), 25.9, 18.3, -5.4, -5.4; **^{31}P{^{1}H} NMR** (202 MHz, $CDCl_3$) δ 157.58; **IR** (neat, cm^{-1}) 1605, 1583, 1509, 1460, 1298, 1248, 1178, 1072, 1030, 956, 828, 774, 704, 583, 448; **HRMS (ESI/QTOF)** $[M + H]^+$ Calcd for $C_{48}H_{58}F_2N_4O_8PSi$ 915.3724; Found 915.3726.

3.Additional Information

3.1 Reaction monitoring by ^{31}P NMR

Scheme S5. Acylation reaction conditions

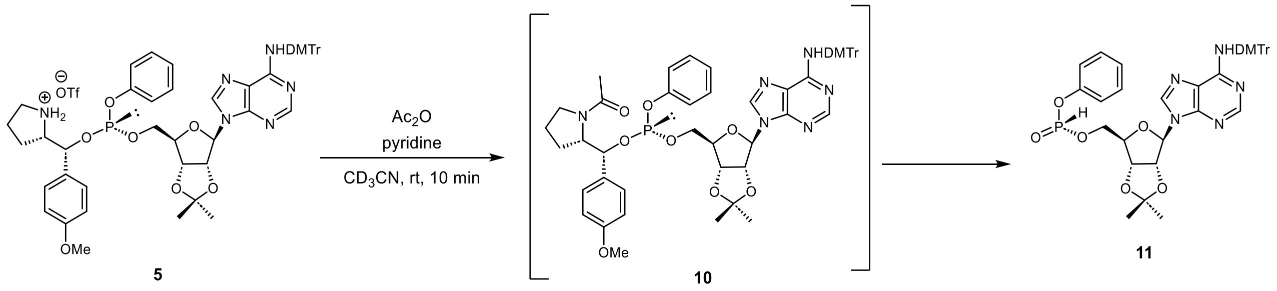

^{31}P NMR spectra of the acetylation step indicated the presence of the *H*-phosphonate **11** (δ_P = 8.9, $^1J_{PH}$ = 719.9 Hz) instead of the acetylated phosphite triester **10** (**Figure S1**).

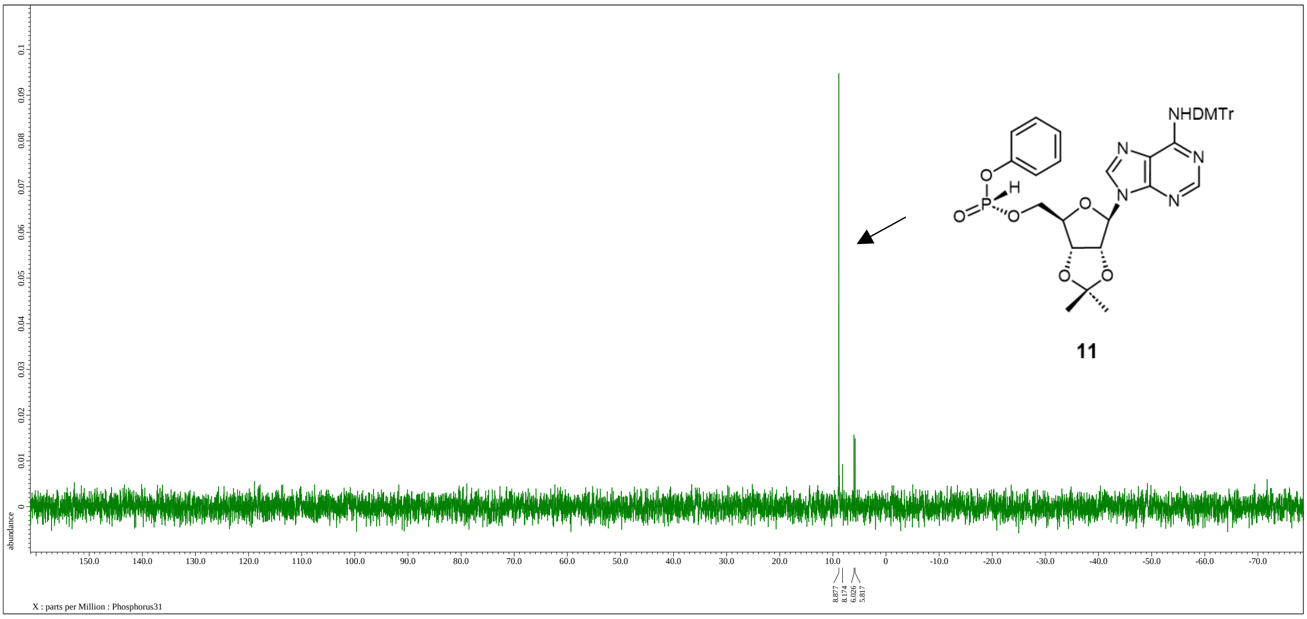

Figure S1. ^{31}P NMR spectra of the acylation step

Scheme S6. Control experiment for the elucidation of the formation mechanism of a byproduct (δ_P = 22.9)

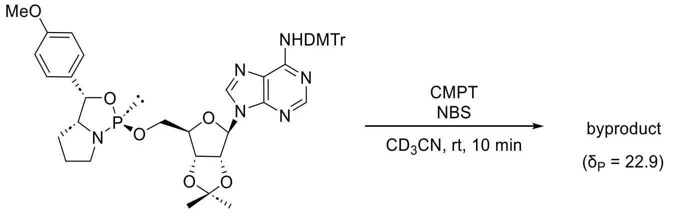

Treatment of **(*R*p)-4a** with CMPT and NBS in the absence of PhOH resulted in the formation of a byproduct (δ_P = 22.9). The byproduct was not characterized because it was decomposed during purification.

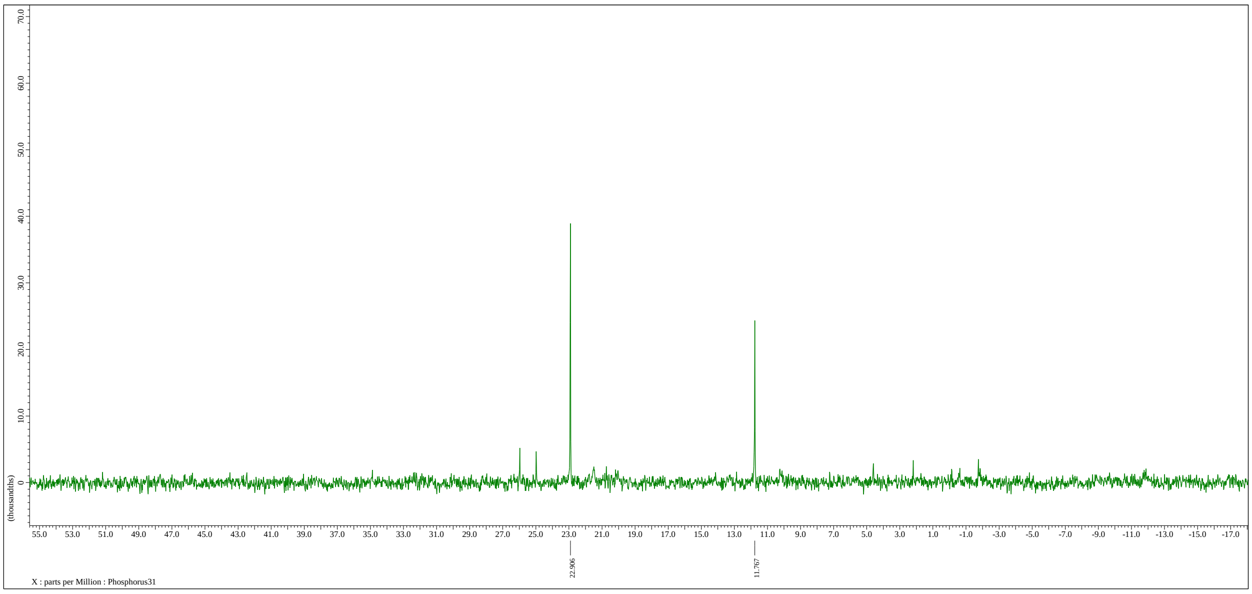

Figure S2. 31P NMR spectrum of Control experiment for the elucidation of the formation mechanism of a byproduct (δ_P = 22.9)

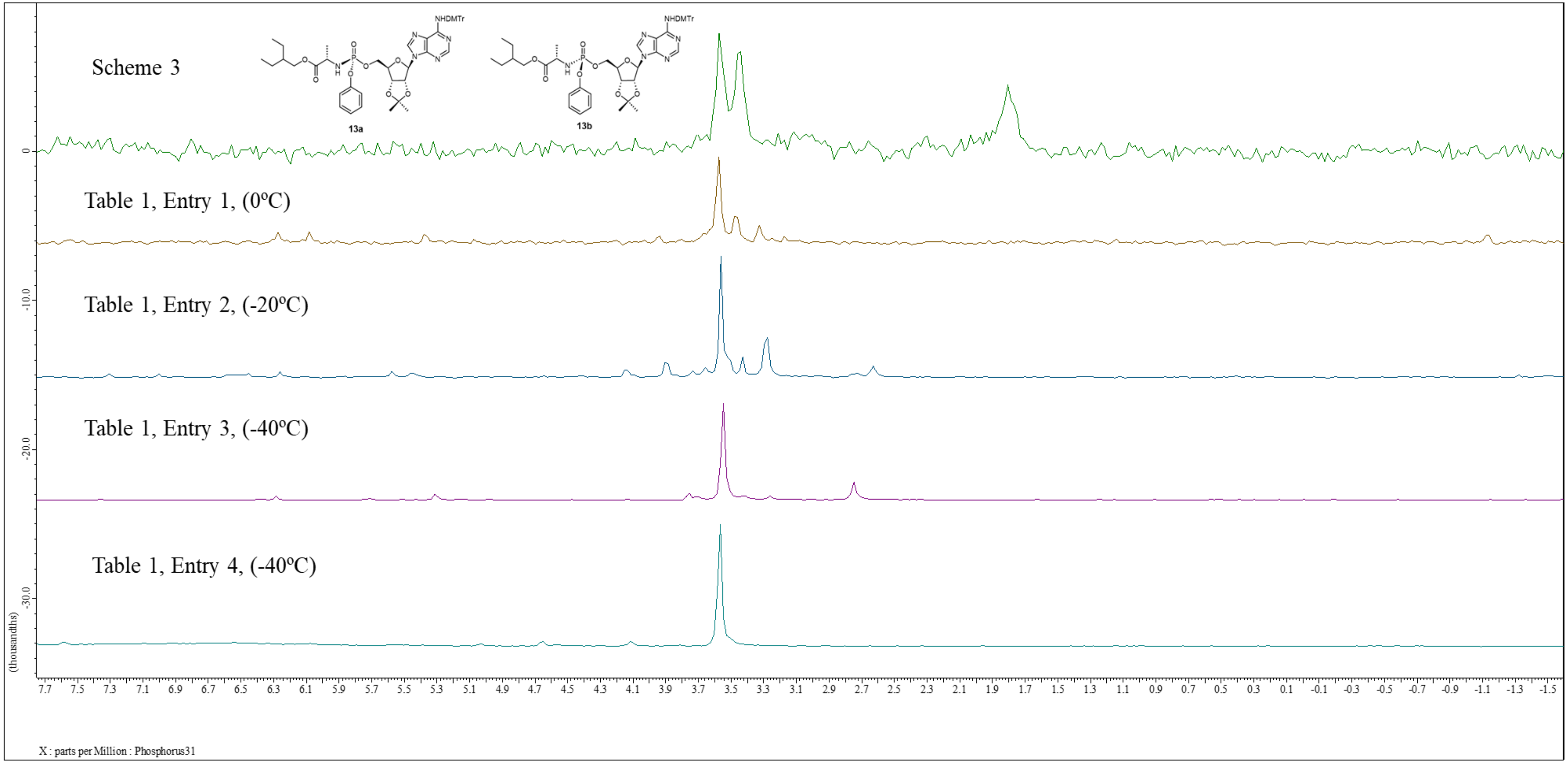

Figure S3. ^{31}P NMR spectra of the reaction mixture of Scheme 3 and Table 1

3.2 Plausible epimerization mechanism of ProTides

Scheme S7. Plausible epimerization mechanism of **Scheme 3**

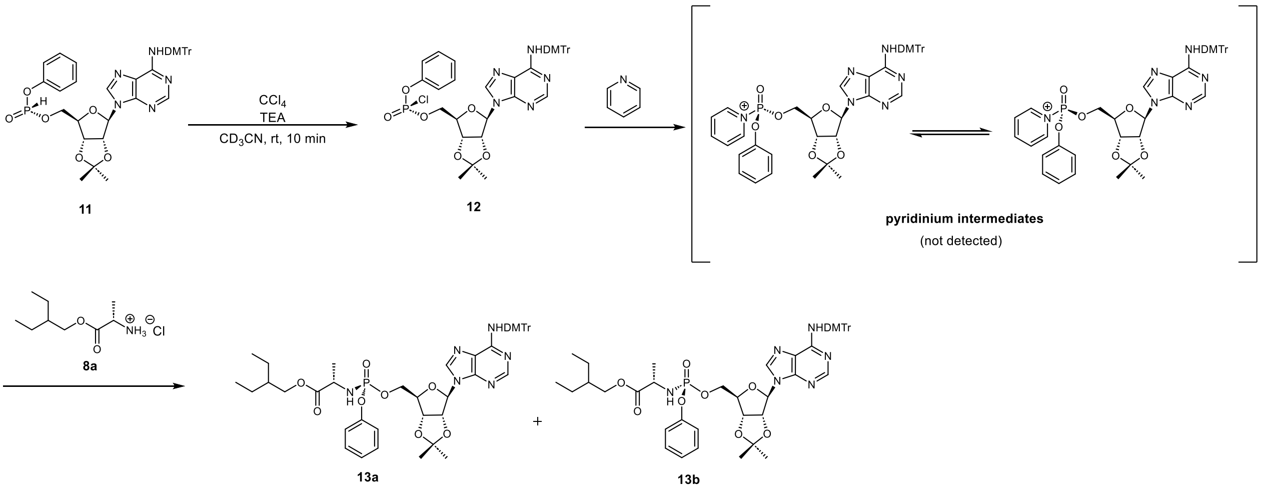

During the Atherton-Todd reaction of the *H*-phosphonate diester to phosphoramidite in the presence of pyridine, phosphorochloridate intermediate **12** would be attacked by pyridine repeatedly, resulting loss of stereopurity. Thus, the use of pyridine should be avoided.

Scheme S8. Plausible epimerization mechanism of **Scheme 4**

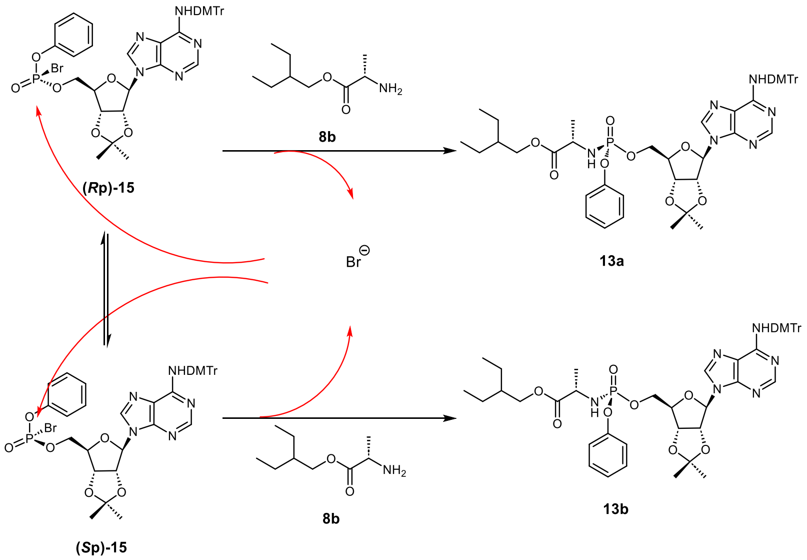

We attributed the low stereoselectivity of the synthesis of compound **13** at relatively high temperature to nucleophilic attack of a bromide ion, generated as a leaving group, to the phosphorobromidate intermediate **15**. This side reaction was suppressed at low temperature.

3.3 Investigation of 3'-*O*-protecting groups of a Sofosbuvir derivative

First, we used 3'-*O*-TBDPS protected 5'-oxazaphospholidine derivative to synthesize Sofosbuvir, but the stereoselectivity was lower than the synthesis of compound **13**, adenosine derivative (Sofosbuvir: dr = 90:10, **Figure S4**, adenosine derivative: dr >99:1). We attributed this result to the steric hinderance of TBDPS group. On the other hand, a synthesis of 5'-oxazaphospholidine derivative whose 3'-hydroxy group was protected by a methoxymethyl (MOM) group, by the phosphitylation reaction gave no stereoselectivity (**Figure S5**). Meanwhile, since the use of a TBDMS group offered a successful synthesis of 5'-oxazapahospholidine and phosphoramidate derivatives, a TBDMS group was chosen for the synthesis of Sofosbuvir.

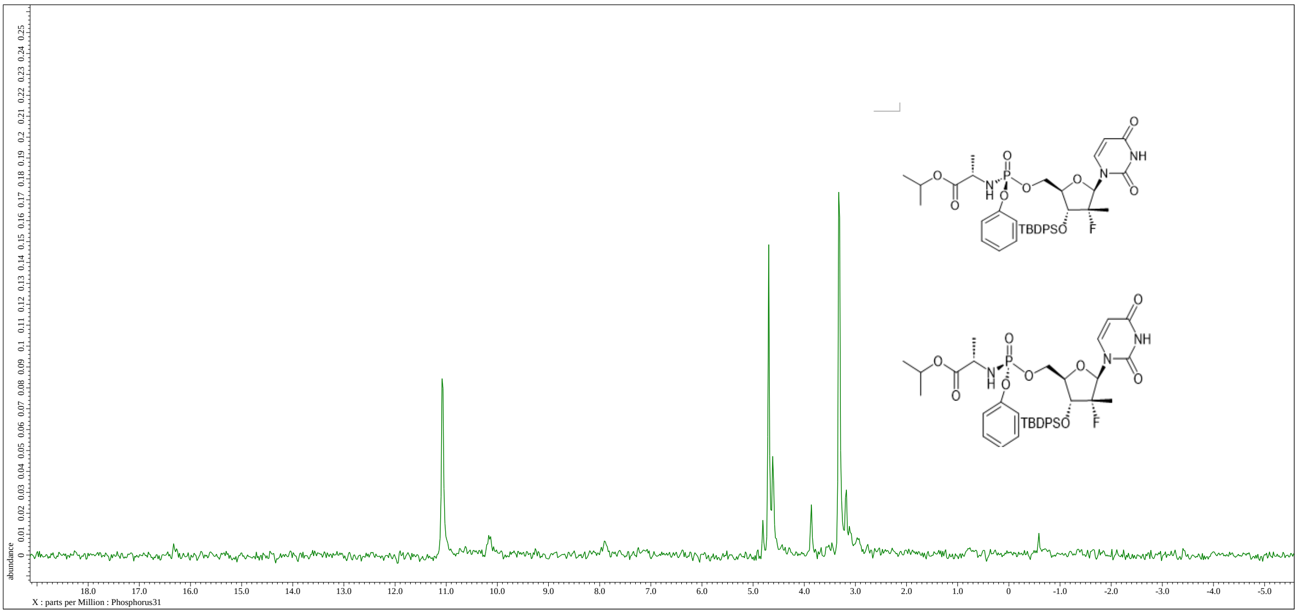

Figure S4. ^{31}P NMR spectrum of protected Sofosbuvir (3'-*O*-TBDPS)

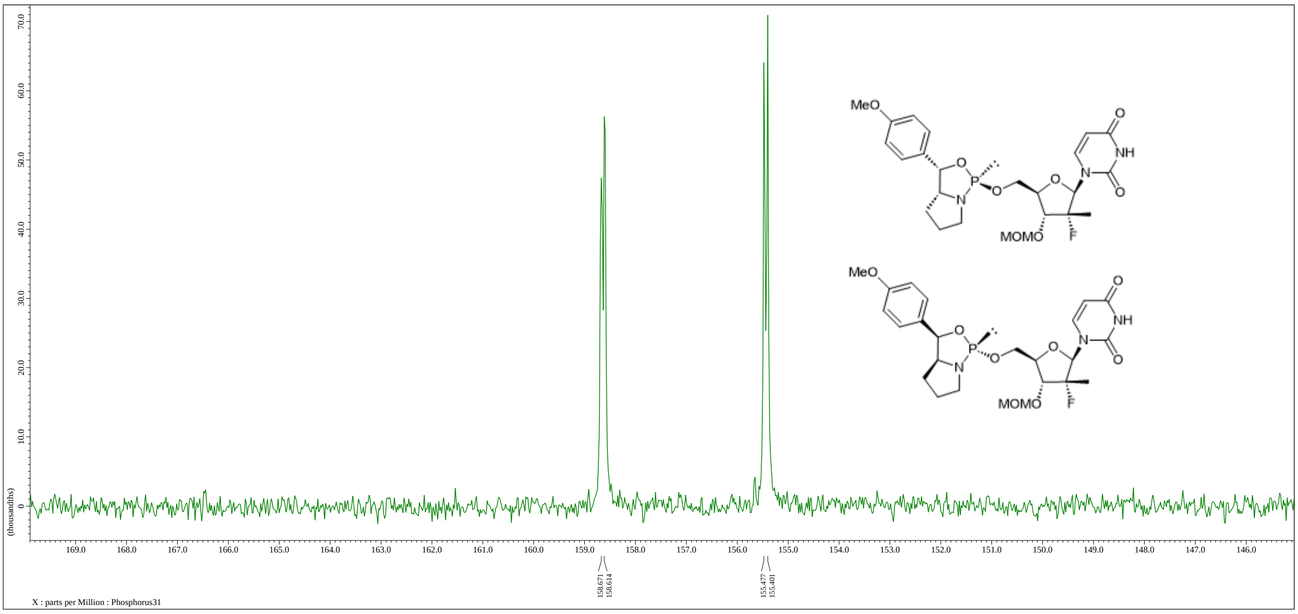

Figure S5. ^{31}P NMR spectrum after purification of 5'-*O*-oxazaphospholidine derivative (3'-*O*-MOM)

4 Copies of RP-HPLC profiles and ^{1}H, ^{13}C, ^{31}P NMR spectra

4.1 Copies of RP-HPLC profiles

Scheme S9. Deprotection of DMTr group of model compound

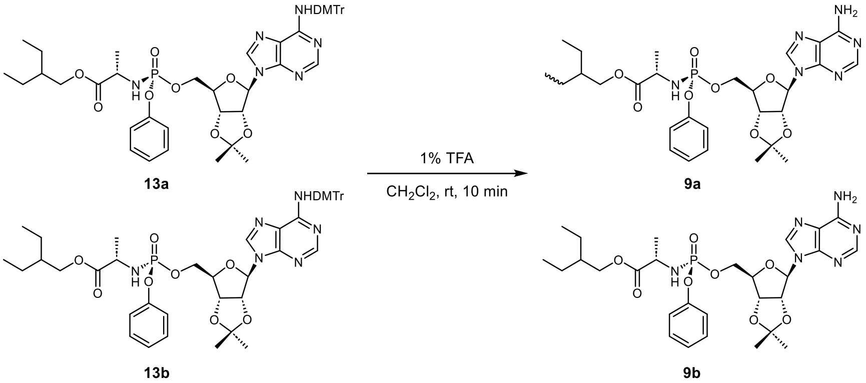

Crude mixture of **13a** and **13b** (0.06 g) (**Scheme 3**) were dissolved in CH_2Cl_2 (8 mL) and 1%TFA (0.08 mL) was added to the solution. After stirred for 10 min, the organic layer was washed with a saturated $NaHCO_3$ aqueous solution (10 mL) and back-extracted with CH_2Cl_2 (10 mL). The organic layers were combined, dried over Na_2SO_4, filtered, and concentrated under reduce pressure. Without further purification, the residue was analyzed by RP-HPLC.

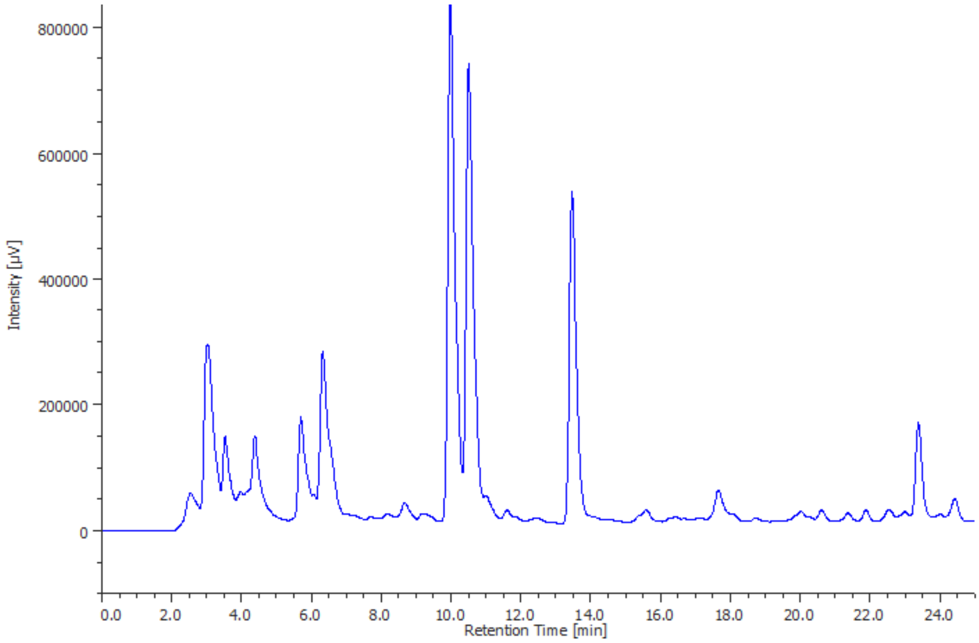

Figure S6. **RP-HPLC profile of crude 9a and 9b (Scheme 3). The compound S8a and S8b were eluted at 10.0 and 10.5 min, respectively.**

RP-HPLC was performed with a linear gradient of 50-100% acetonitrile for 25 min in 0.1 M triethylammonium acetate buffer (pH 7.0) at 30 °C at flow rate of 0.5 mL/min.

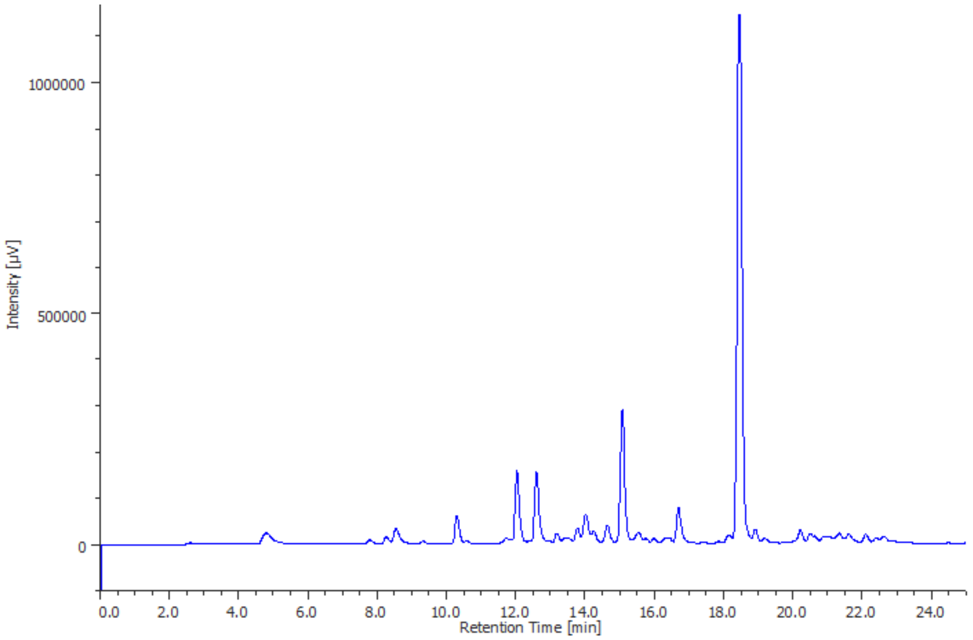

Figure S7. RP-HPLC profile of crude 9a and 9b (Table 1, Entry 4). The compound 9a was eluted at 18.5 min.

RP-HPLC was performed with a linear gradient of 25-100% acetonitrile for 25 min in 0.1 M triethylammonium acetate buffer (pH 7.0) at 30 ºC at flow rate of 0.5 mL/min.

4.2 Copies of ^{1}H, ^{13}C, and ^{31}P NMR spectra

^{1}H NMR (500 MHz, DMSO-d6) of S1

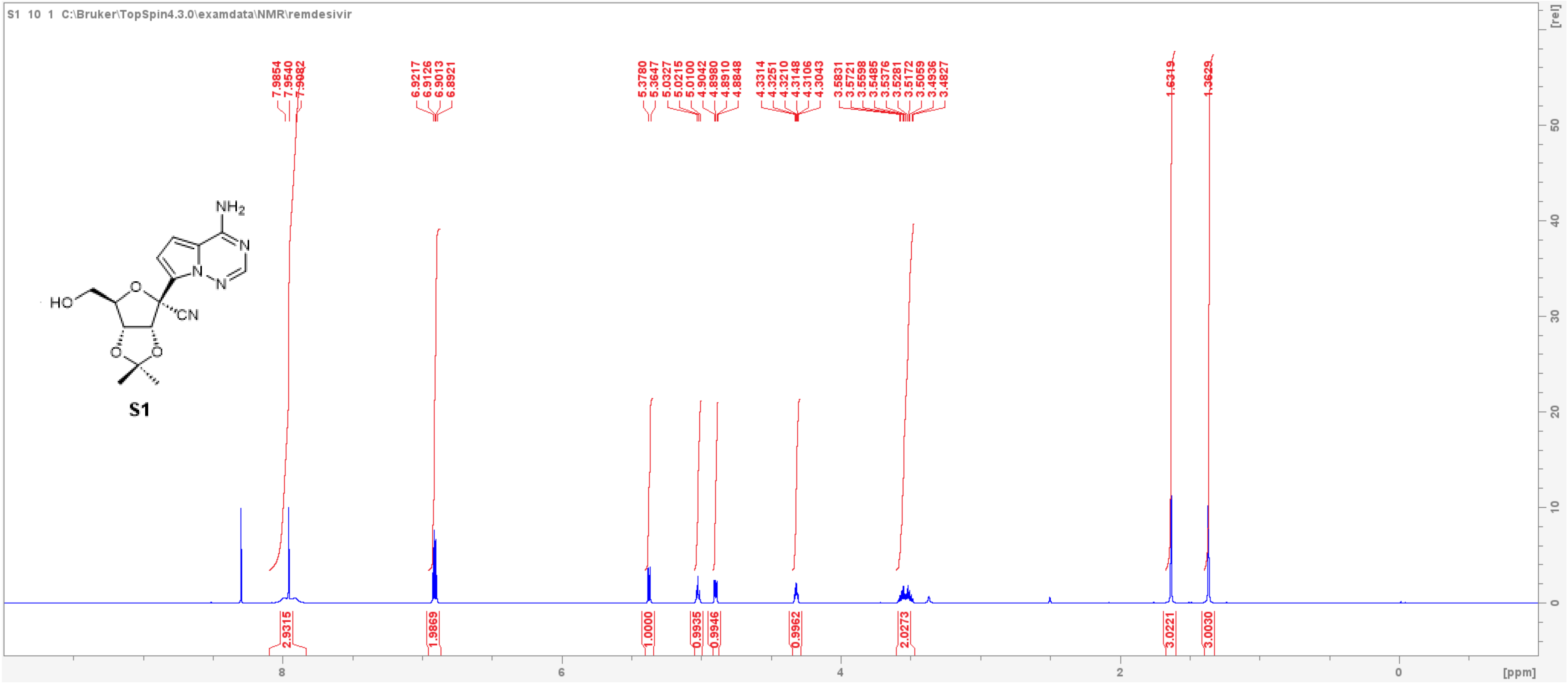

^{1}H NMR (500 MHz, DMSO-d6) of S2

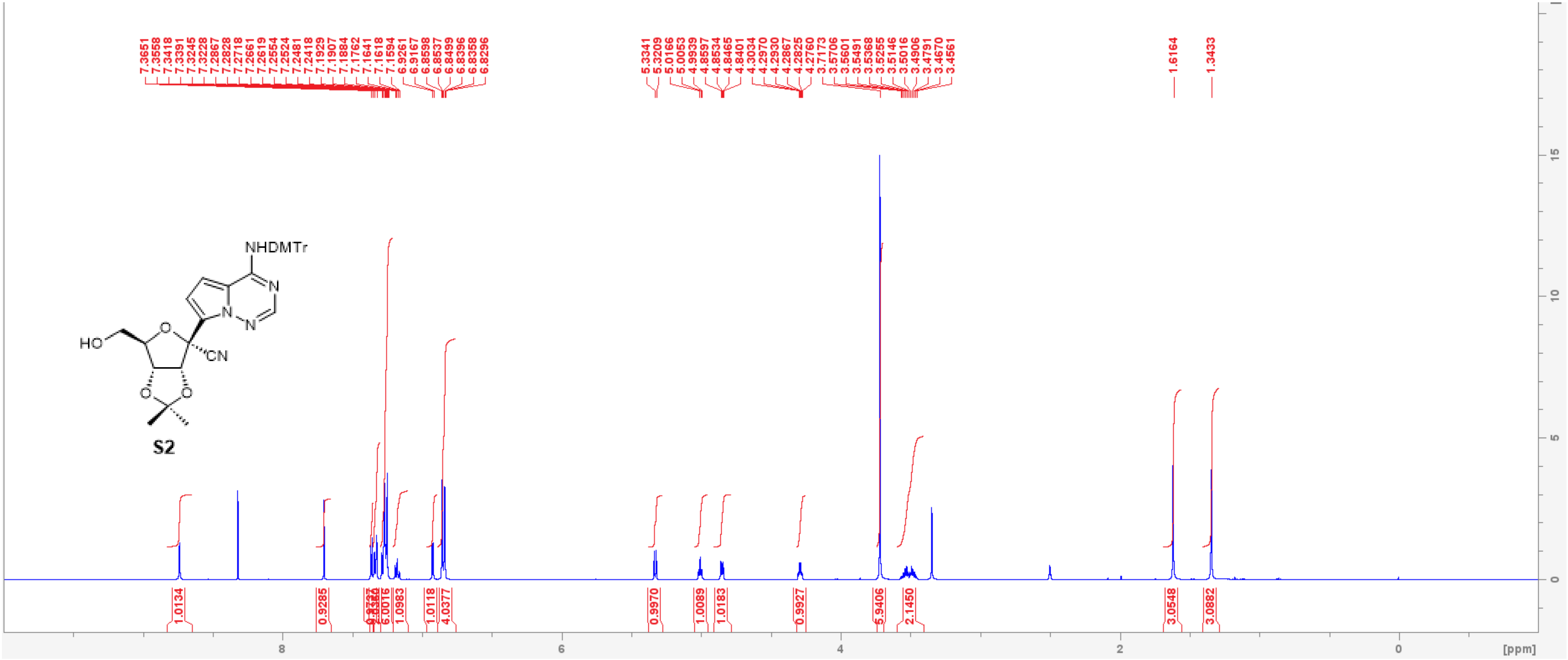

^{13}C NMR (126 MHz, DMSO-d6) of S2

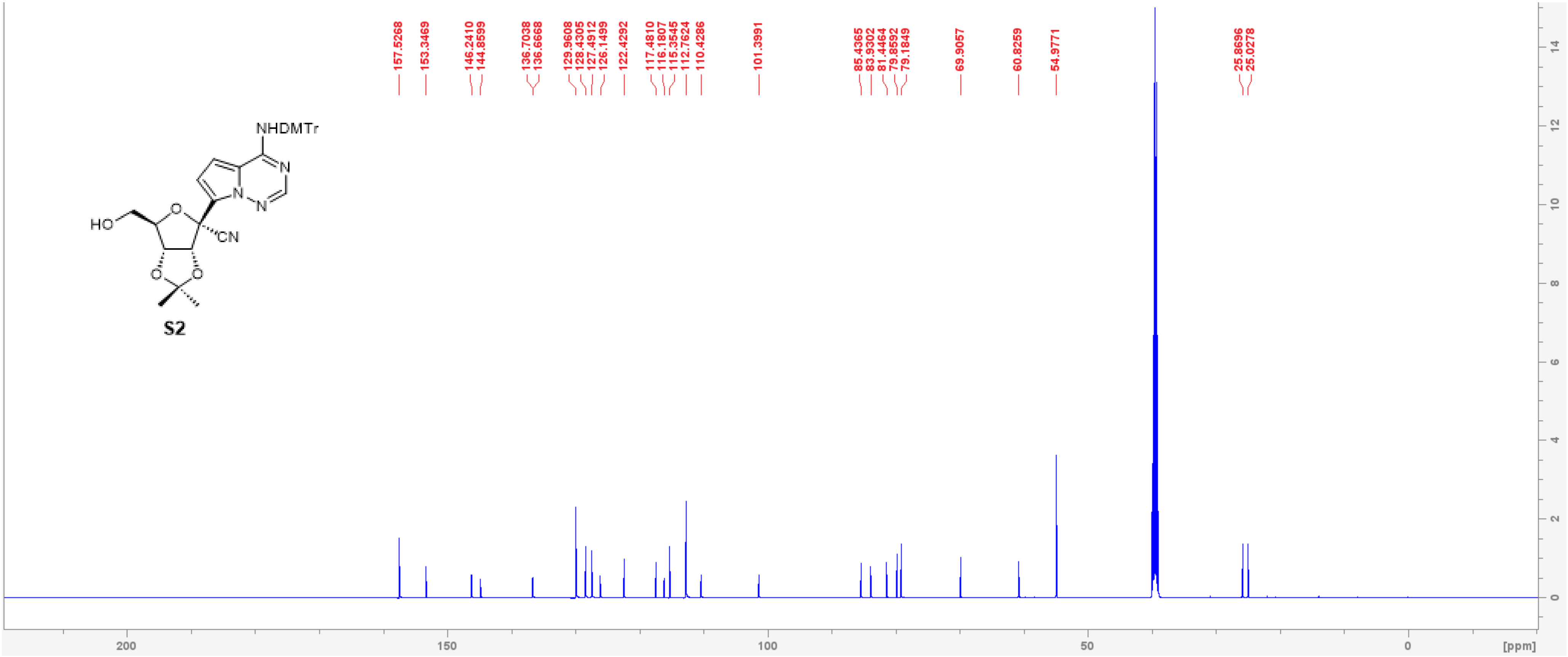

^{1}H NMR (400 MHz, DMSO-d6) of S3

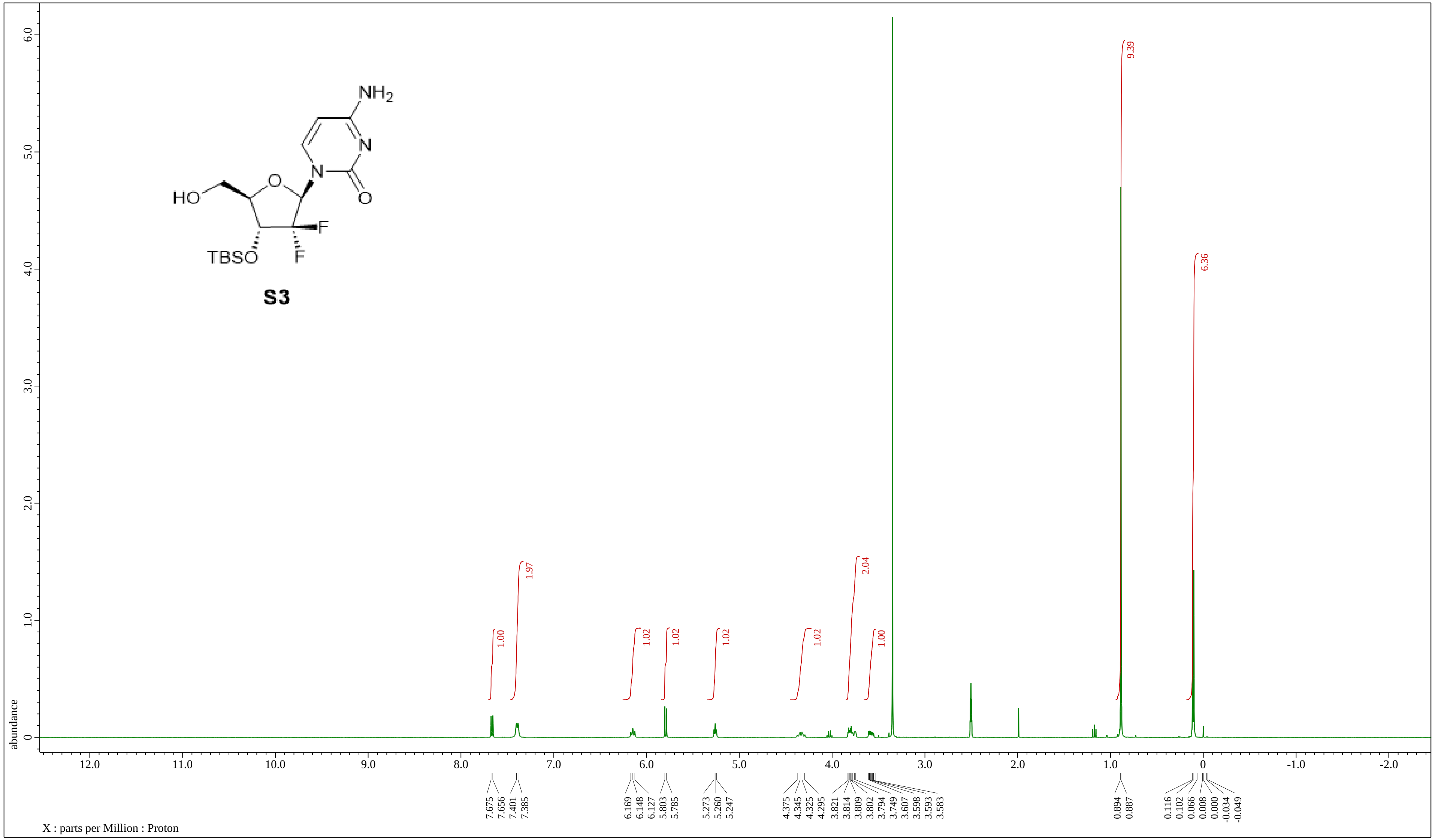

^{1}H NMR (500 MHz, DMSO-d6) of S4

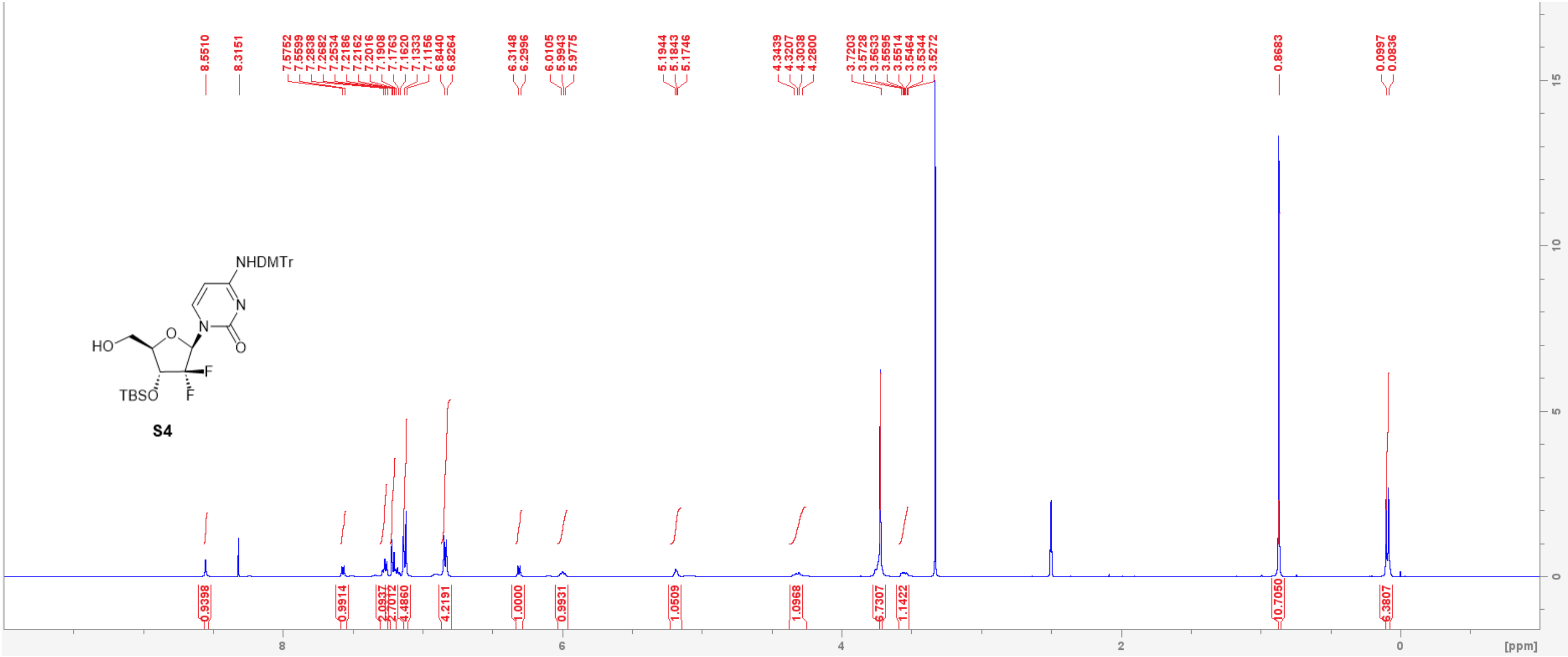

^{13}C {^{1}H} NMR (126 MHz, DMSO-d6) of S4

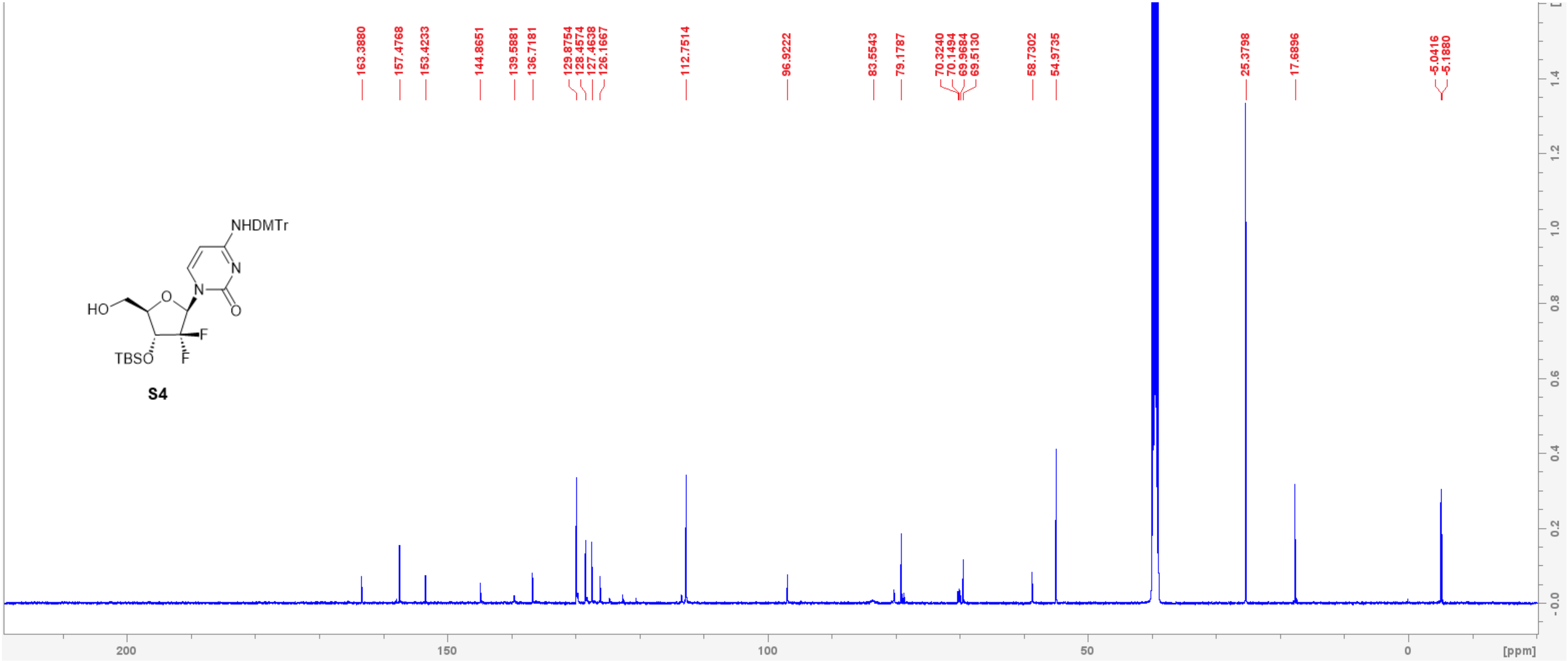

^{1}H NMR (500 MHz, CD_3OD) of S8

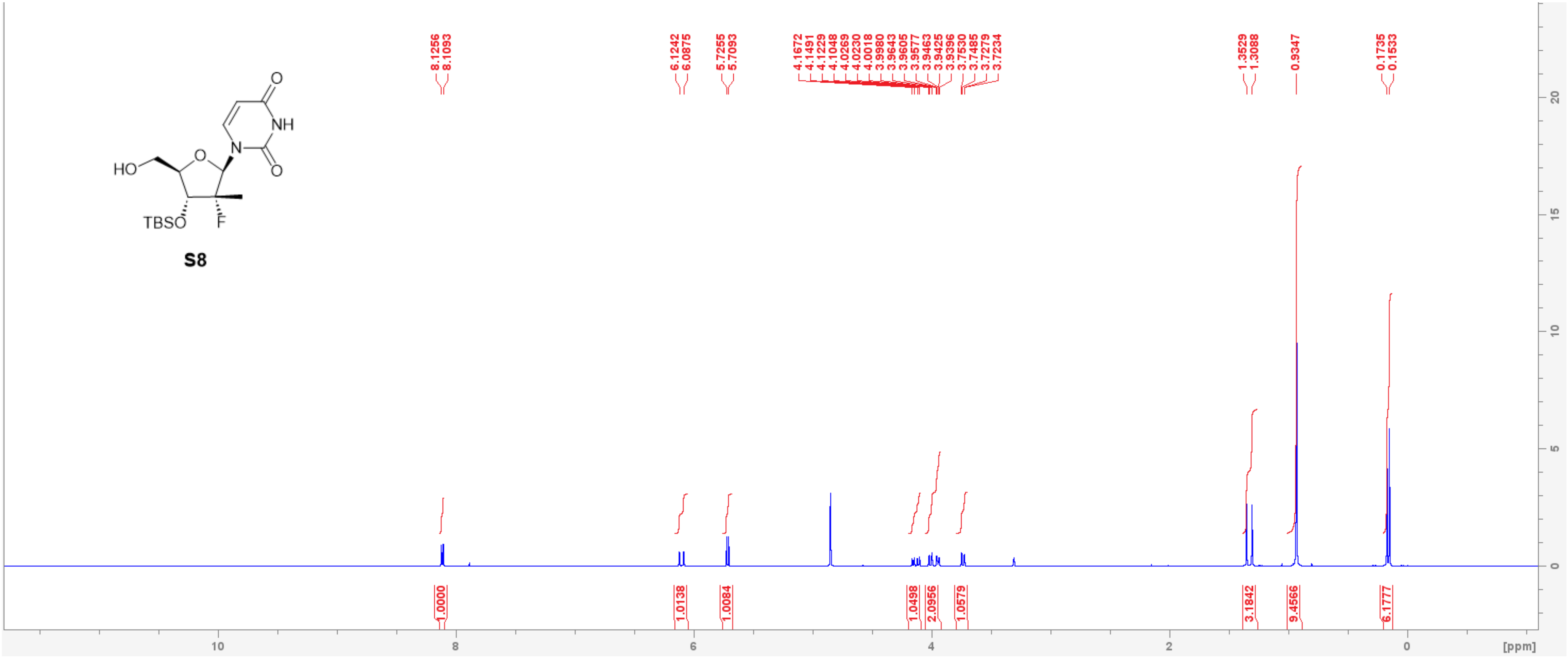

^{1}H NMR (500 MHz, CDCl$_3$) of 5'-*O*-oxazaphospholidine derivative: (*R*p)-4a

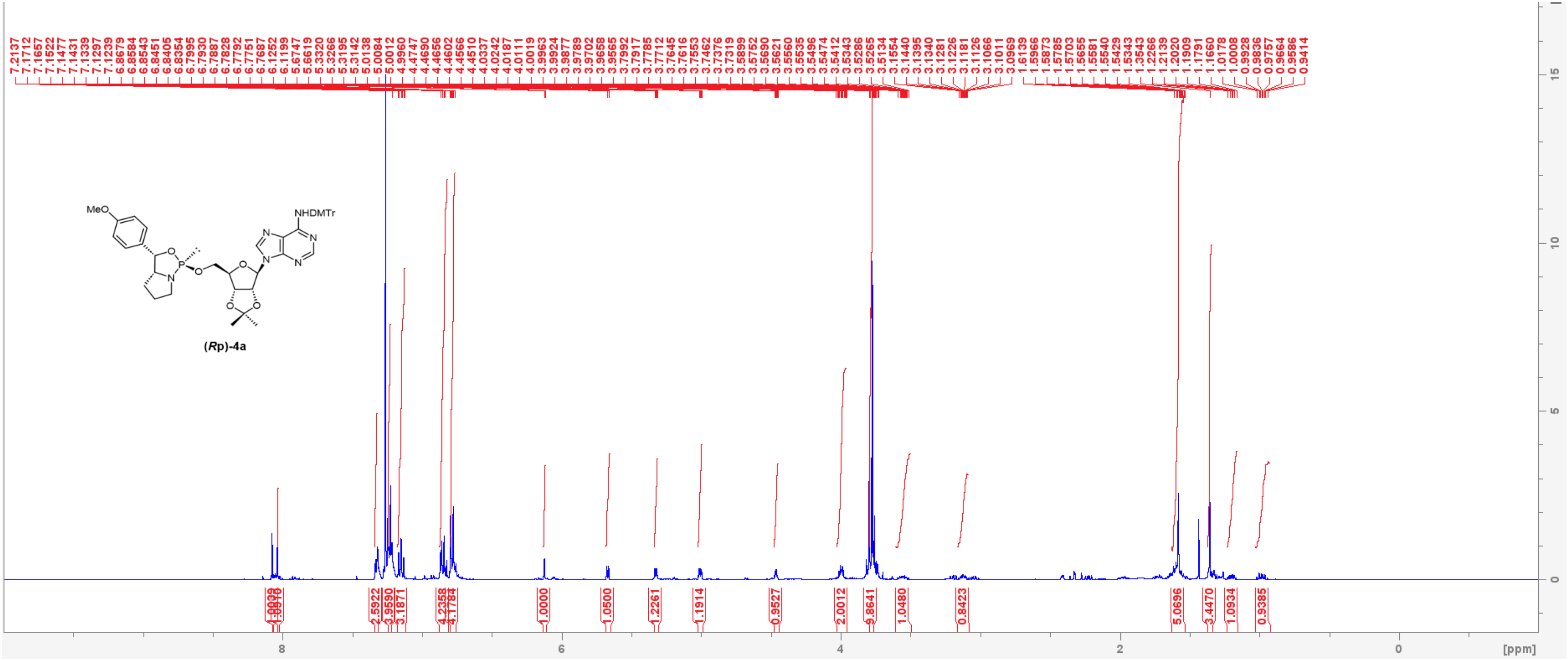

^{13}C {^{1}H} NMR (126 MHz, $CDCl_3$) of 5'-*O*-oxazaphospholidine derivative: (*R*p)-4a

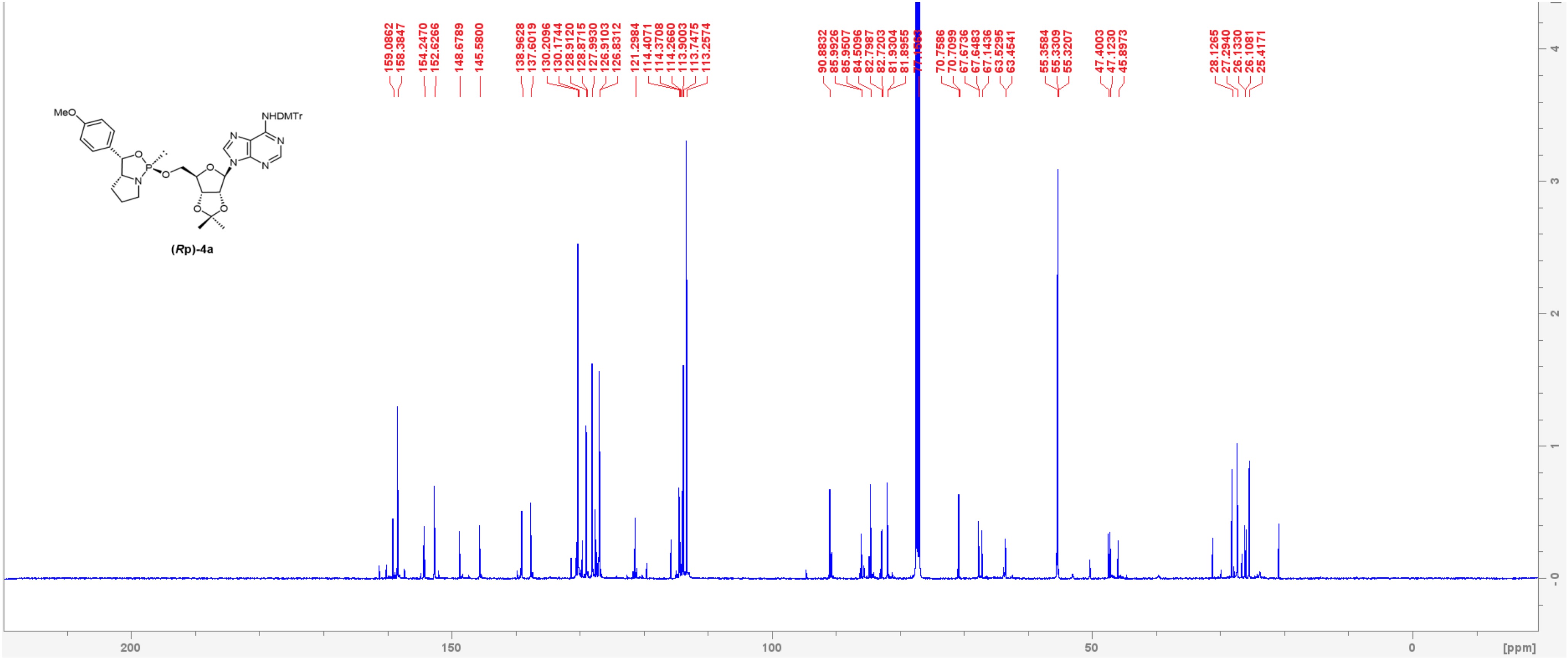

^{31}P {^{1}H} NMR (202 MHz, CDCl$_3$) of 5'-*O*-oxazaphospholidine derivative: (*R*p)-4a

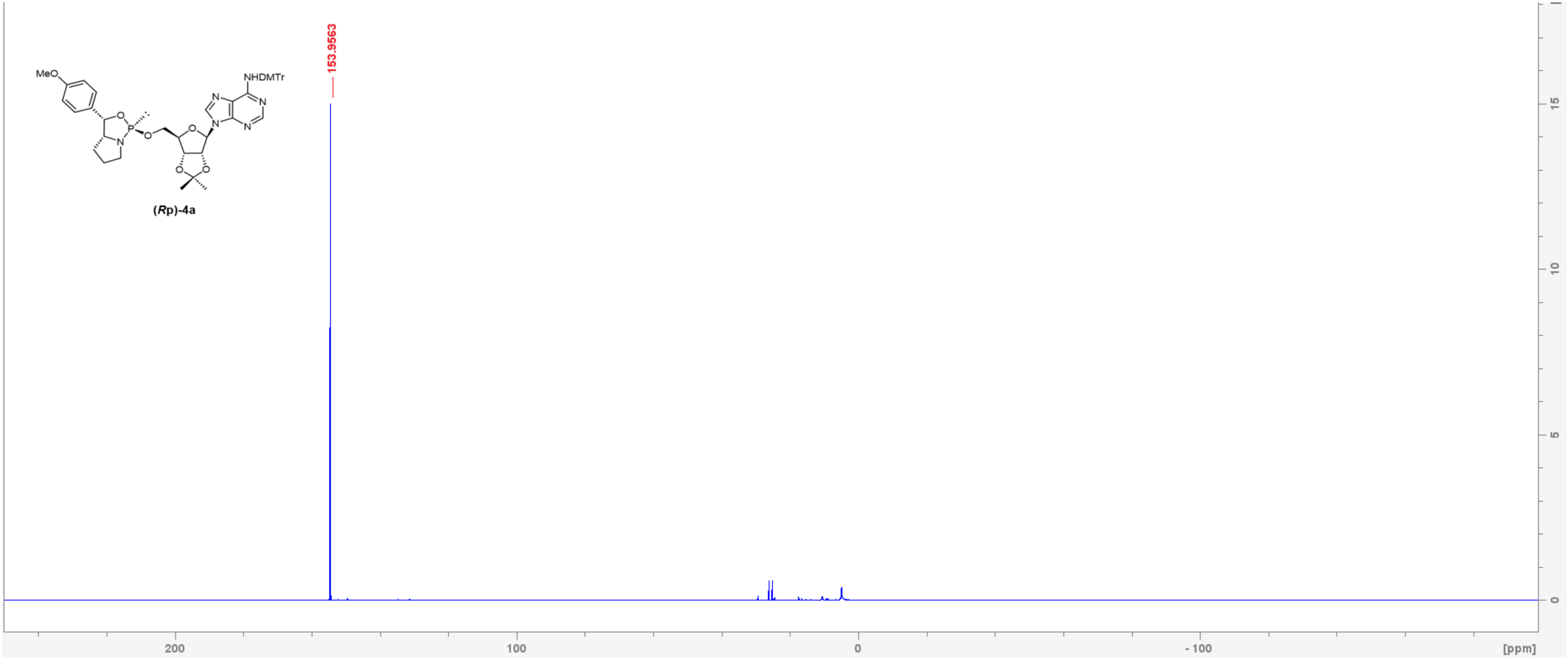

^{1}H NMR (400MHz, CDCl$_3$) of 5'-*O*-oxazaphospholidine derivative: (*S*p)-4a

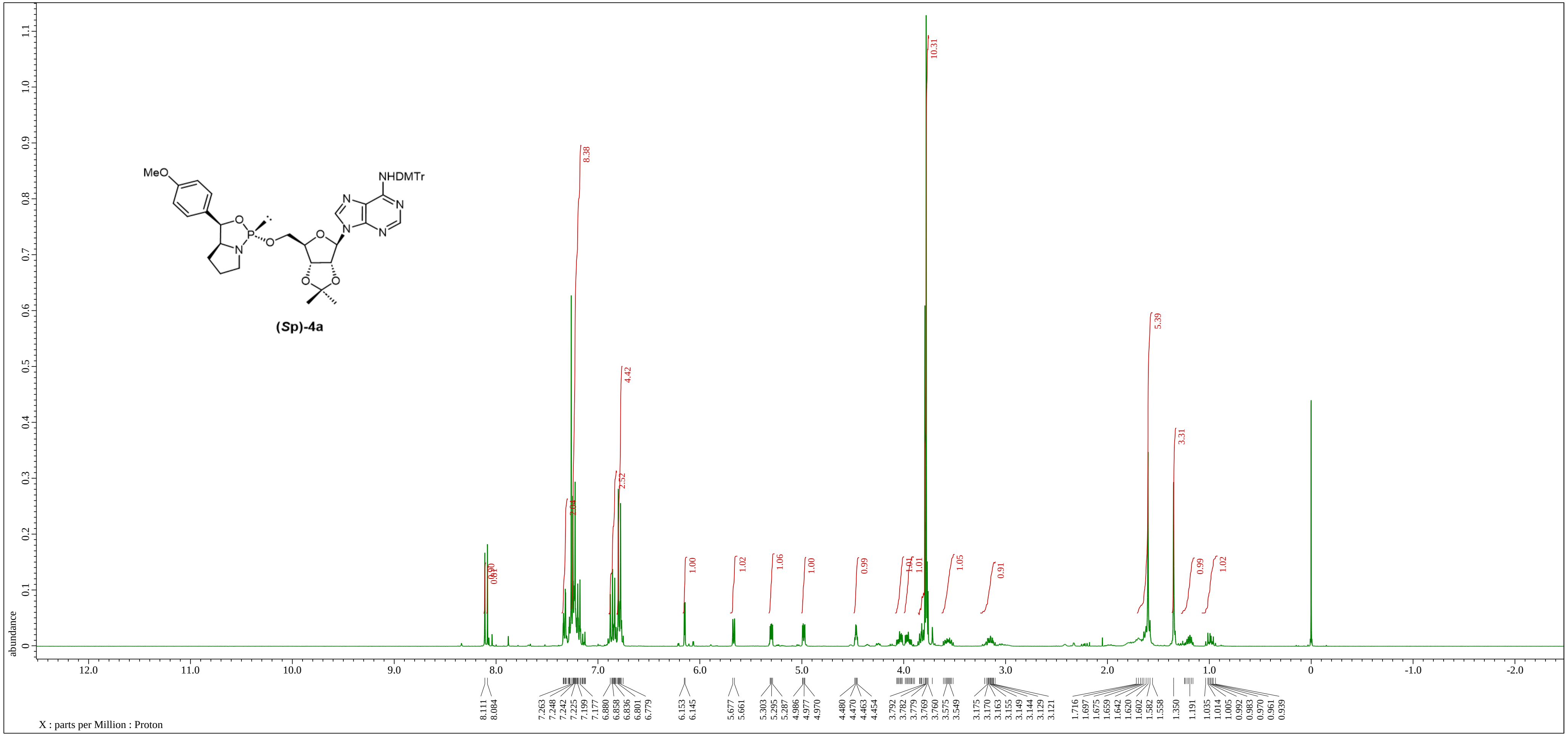

^{13}C {^{1}H} NMR (101 MHz, CDCl$_3$) of 5'-*O*-oxazaphospholidine derivative: (*S*p)-4a

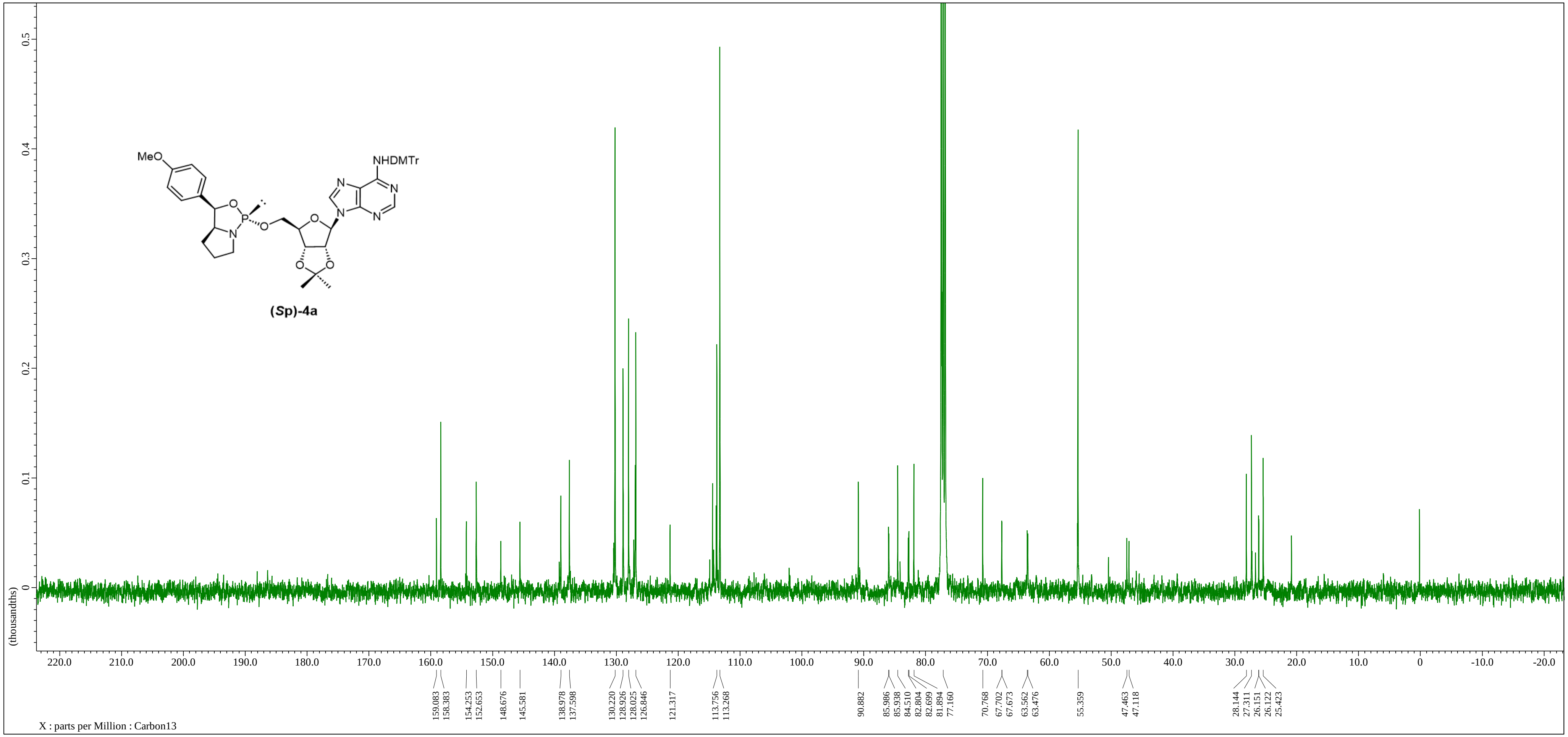

^{31}P {^{1}H} NMR (162 MHz, $CDCl_3$) of 5'-*O*-oxazaphospholidine derivative: (*S*p)-4a

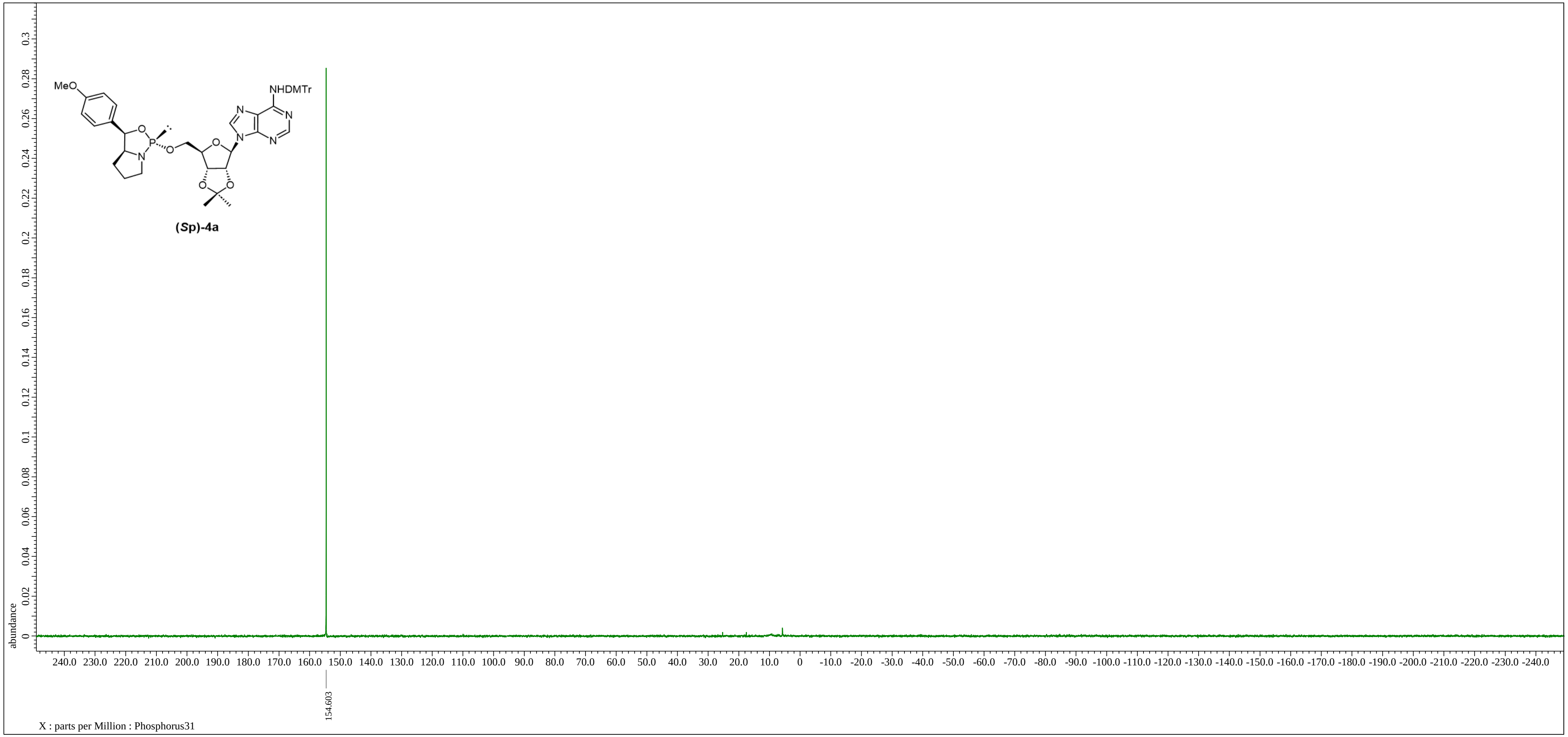

^{1}H NMR (500 MHz, CD_3CN) of 5'-*O*-oxazaphospholidine derivative: (*R*p)-4b

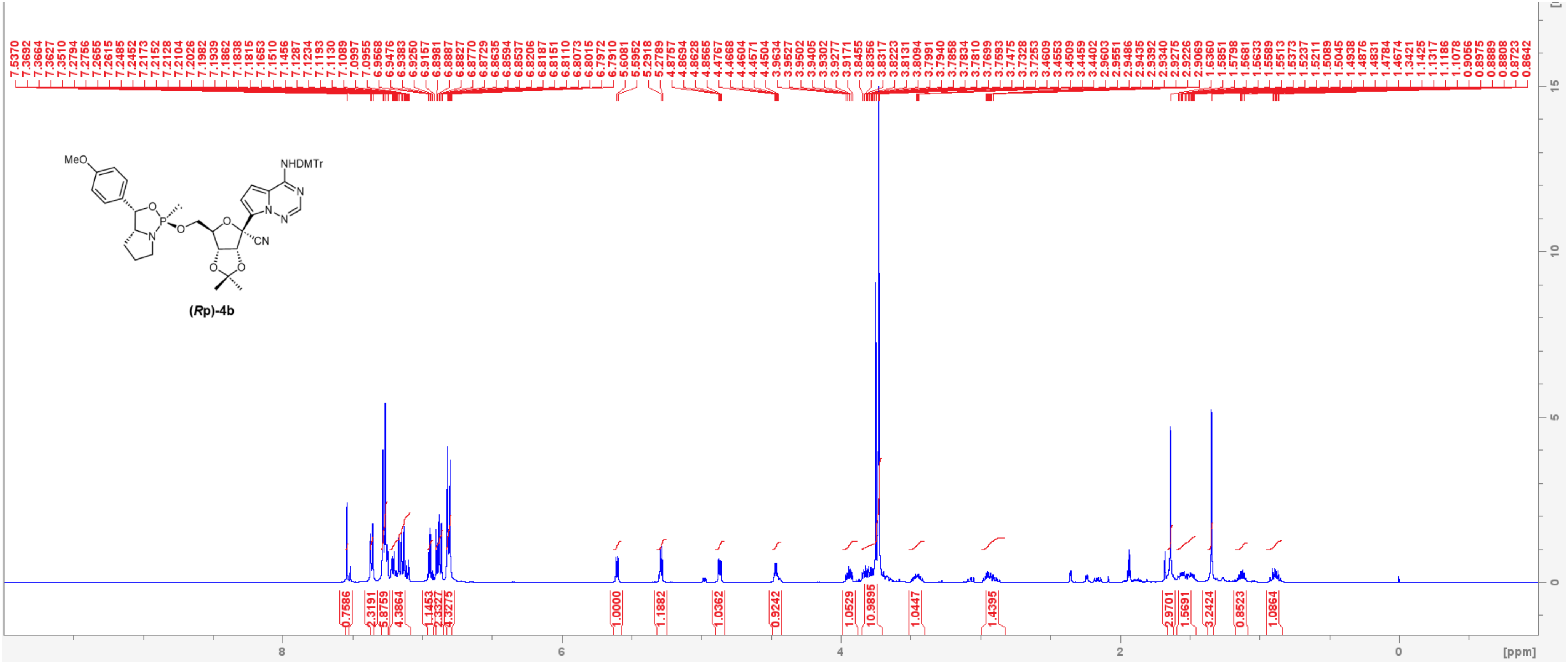

^{13}C {^{1}H} NMR (126 MHz, CD_3CN) of 5'-*O*-oxazaphospholidine derivative: (*R*p)-4b

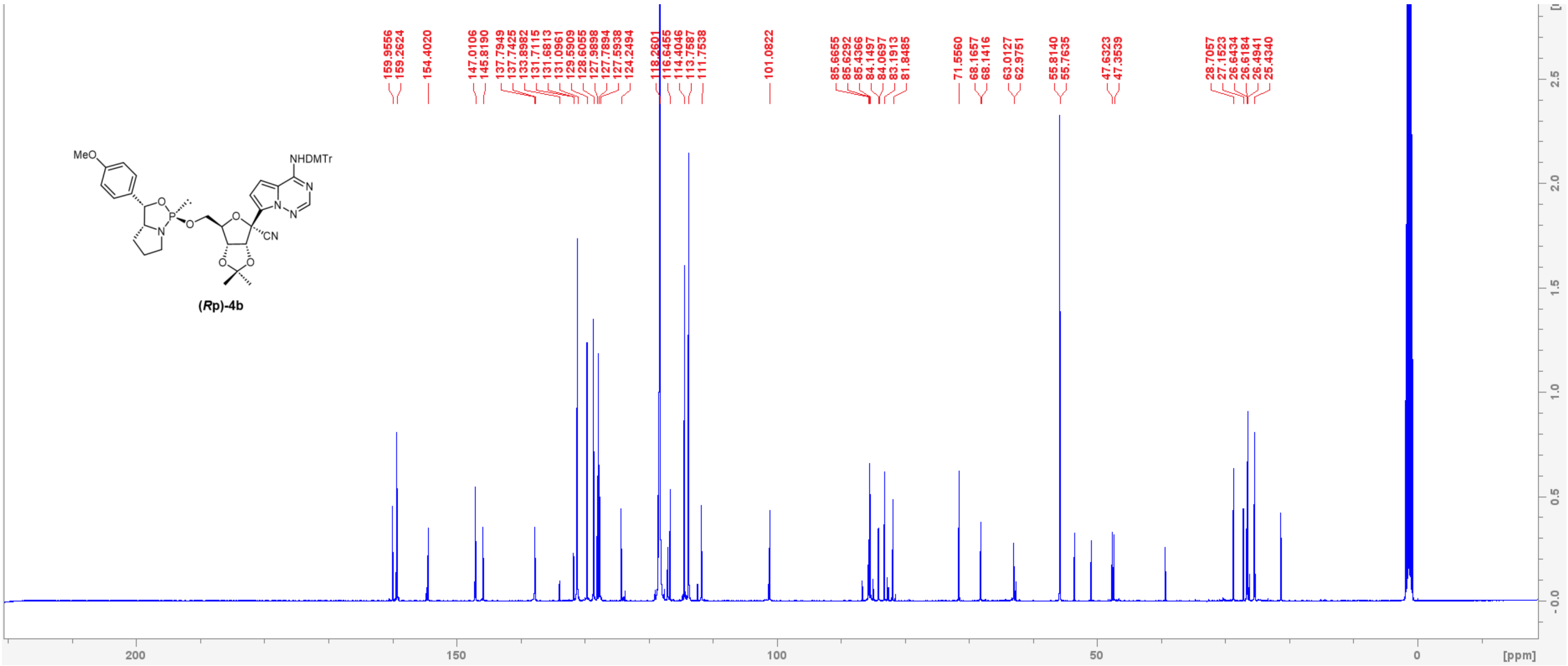

^{31}P {^{1}H} NMR (202 MHz, CD_3CN) of 5'-*O*-oxazaphospholidine derivative: (*R*p)-4b

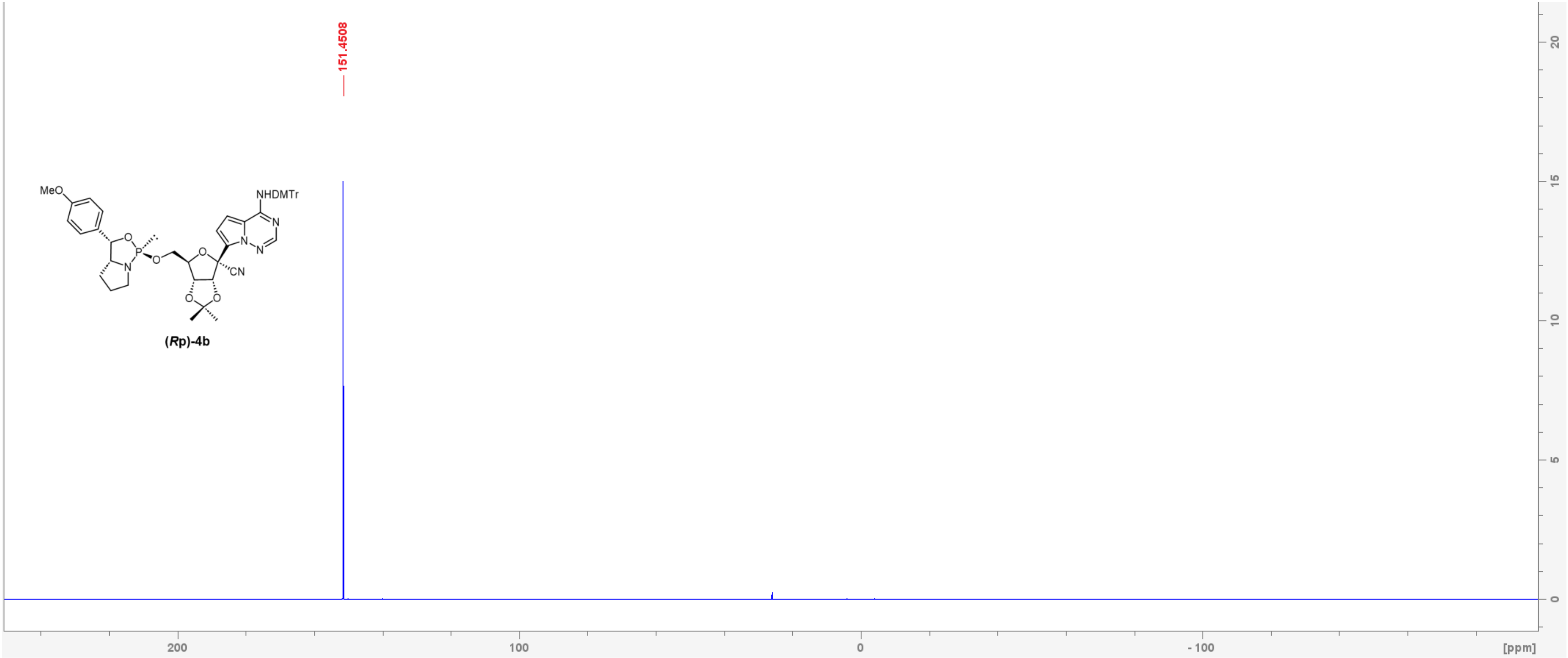

^{1}H NMR (500 MHz, $CDCl_3$) of 5'-*O*-oxazaphospholidine derivative: (*S*p)-4b

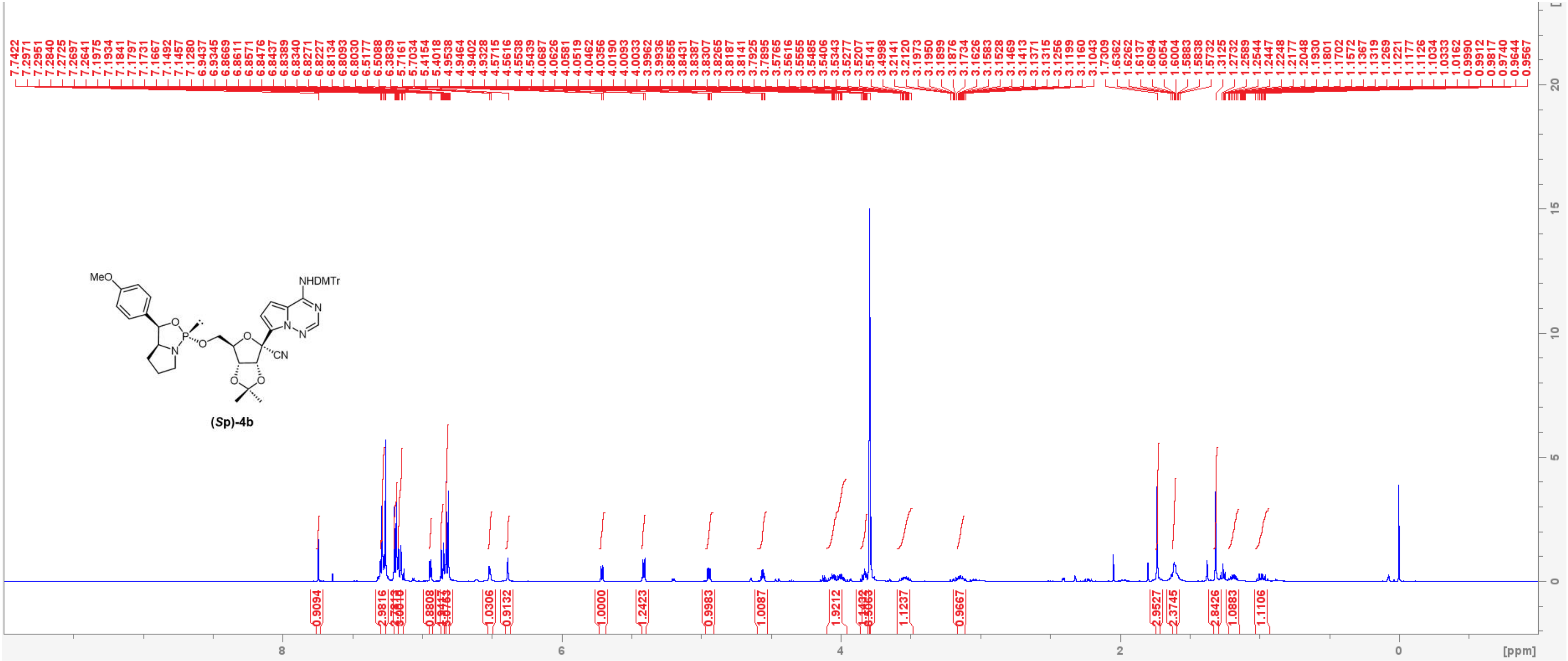

$^{13}C\ \{^{1}H\}$ NMR (126 MHz, $CDCl_3$) of 5'-*O*-oxazaphospholidine derivative: (*S*p)-4b

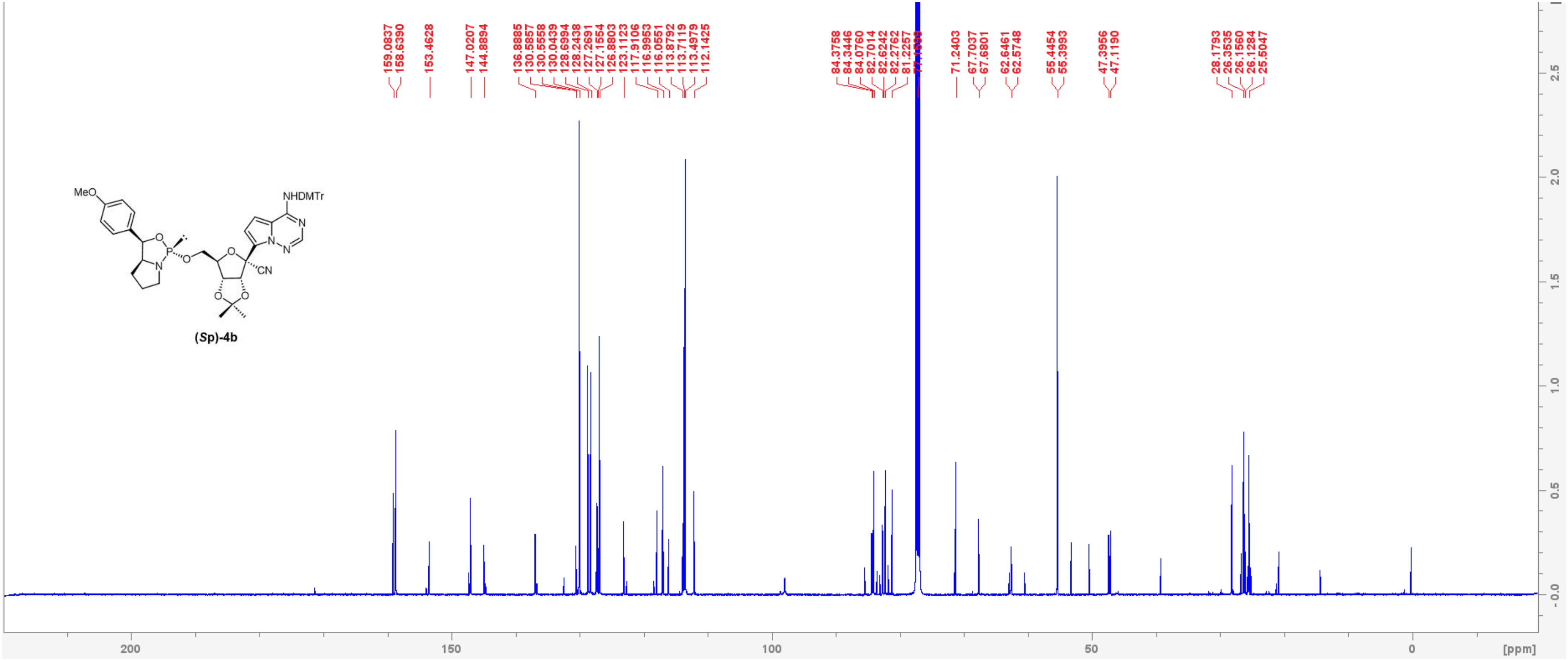

^{31}P {^{1}H} NMR (202 MHz, CDCl$_3$) of 5'-*O*-oxazaphospholidine derivative: (*S*p)-4b

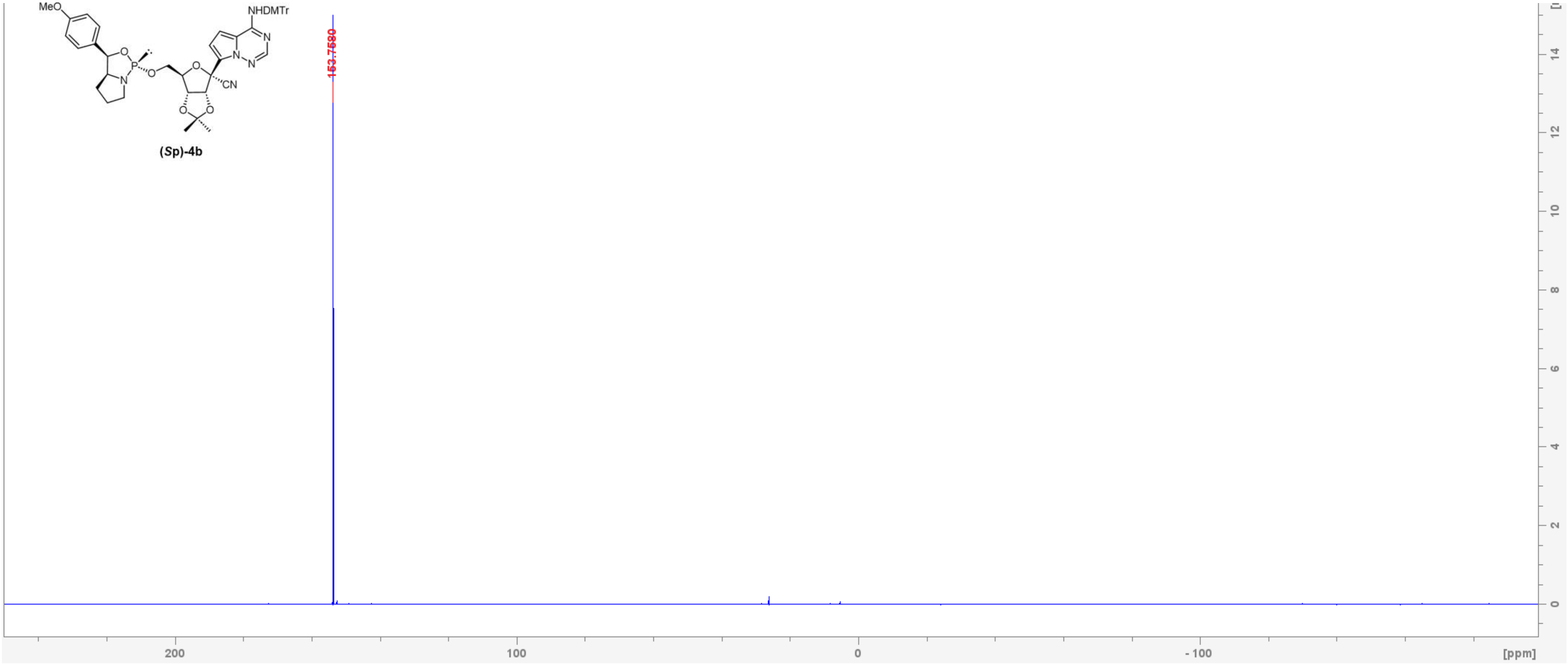

^{1}H NMR (500 MHz, CDCl$_3$) of 5'-*O*-oxazaphospholidine derivative: (*S*p)-4c

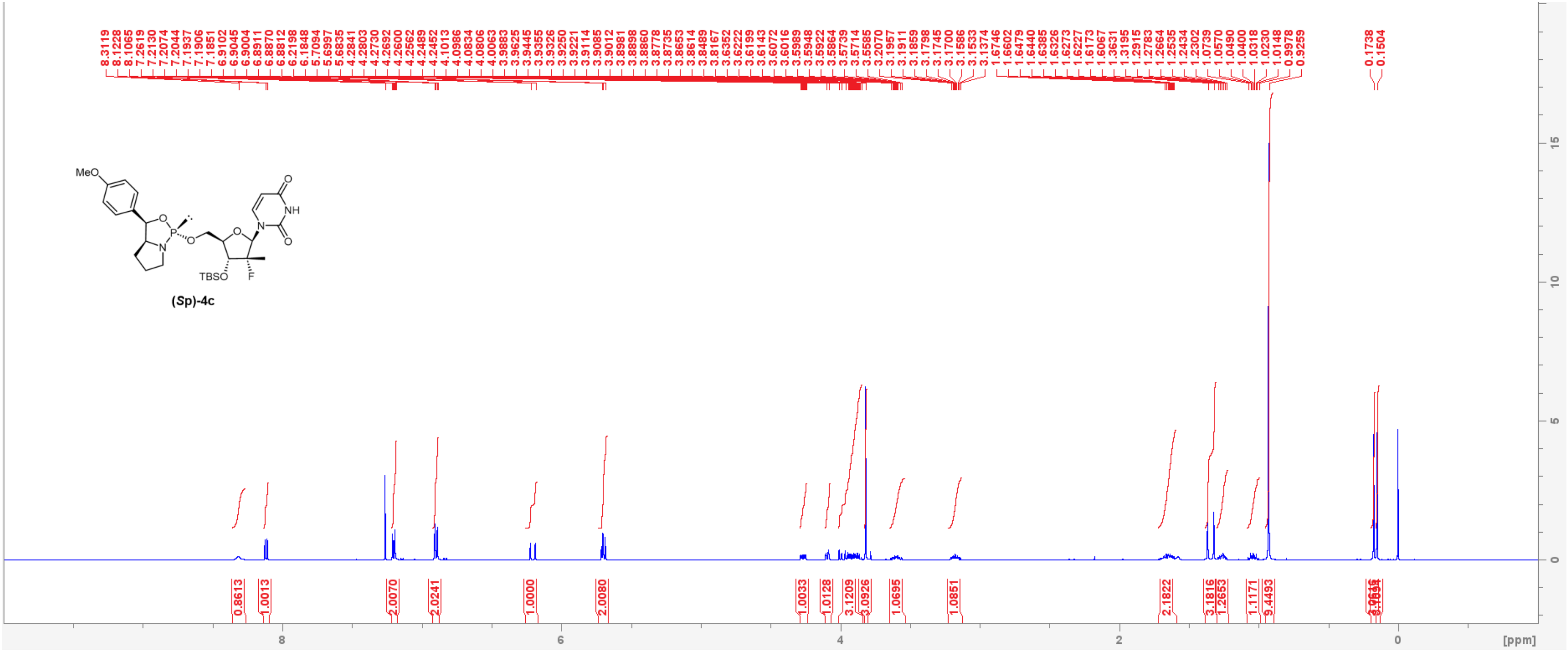

^{13}C {^{1}H} NMR (126 MHz, $CDCl_3$) of 5'-*O*-oxazaphospholidine derivative: (*S*p)-4c

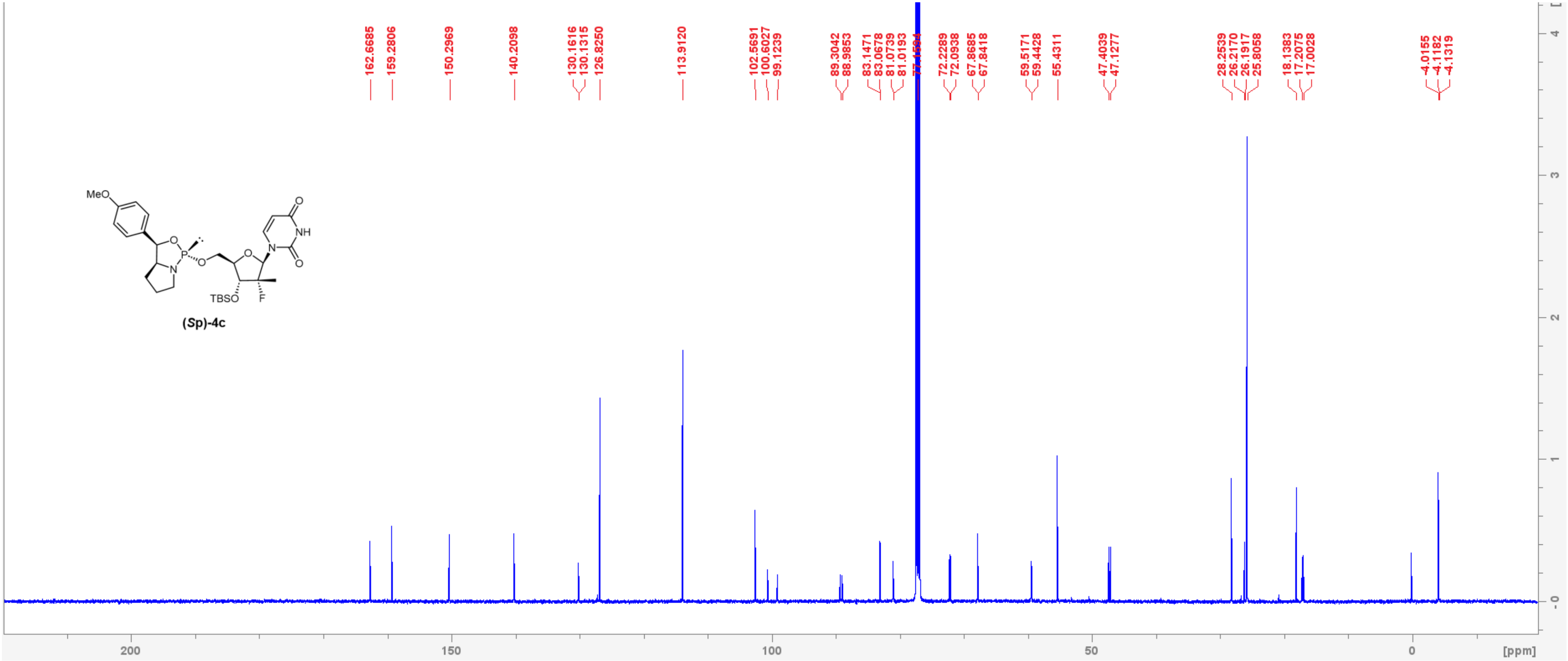

^{31}P {^{1}H} NMR (202 MHz, CDCl$_3$) of 5'-*O*-oxazaphospholidine derivative: (*S*p)-4c

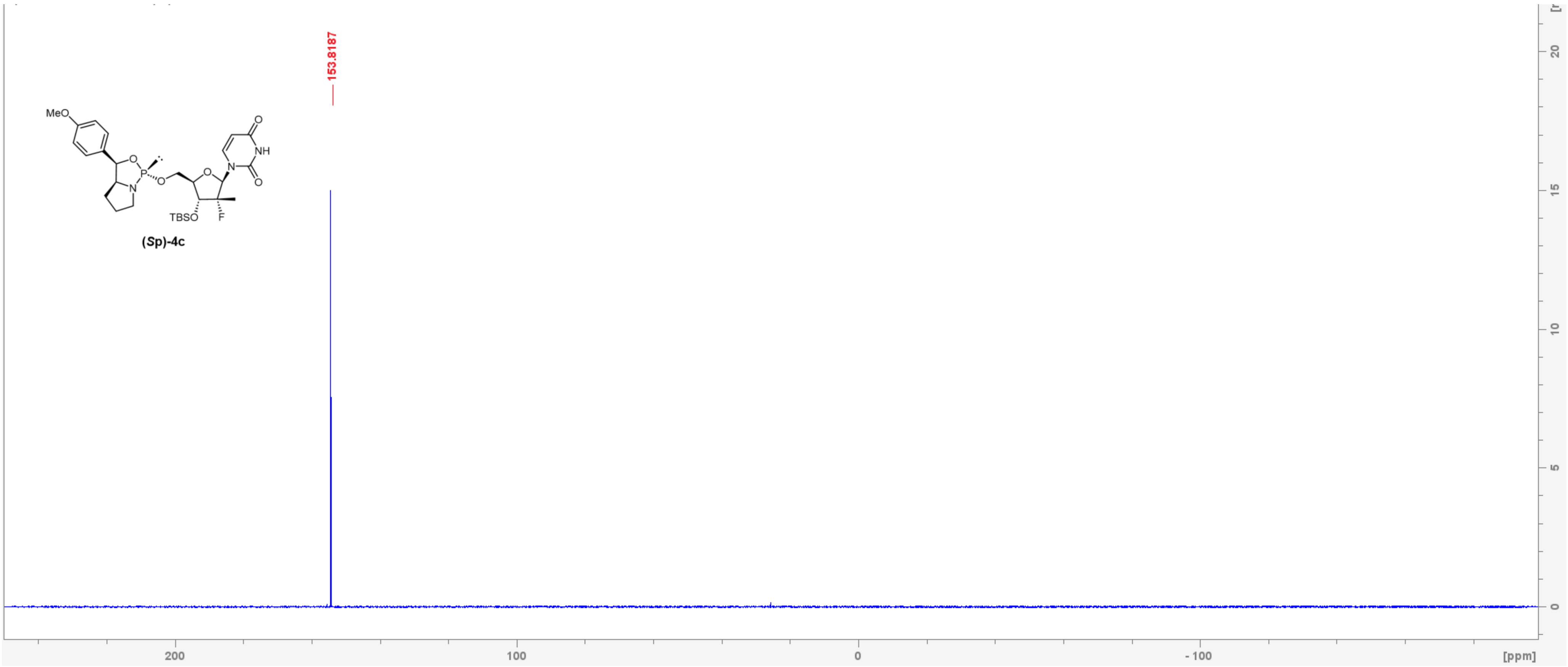

^{1}H NMR (500 MHz, CDCl$_3$) of 5'-*O*-oxazaphospholidine derivative: (*R*p)-4c

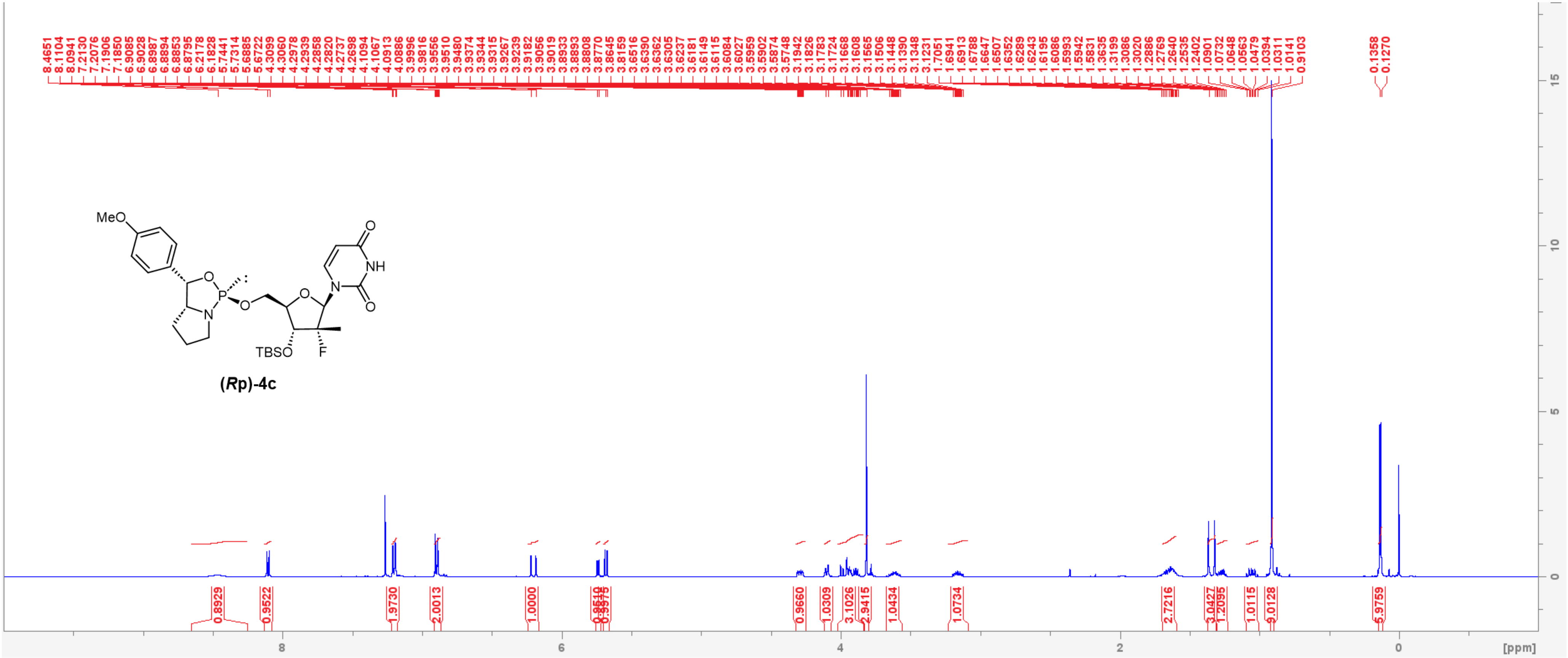

$^{13}C\{^1H\}$ NMR (126 MHz, $CDCl_3$) of 5'-*O*-oxazaphospholidine derivative: (*R*p)-4c

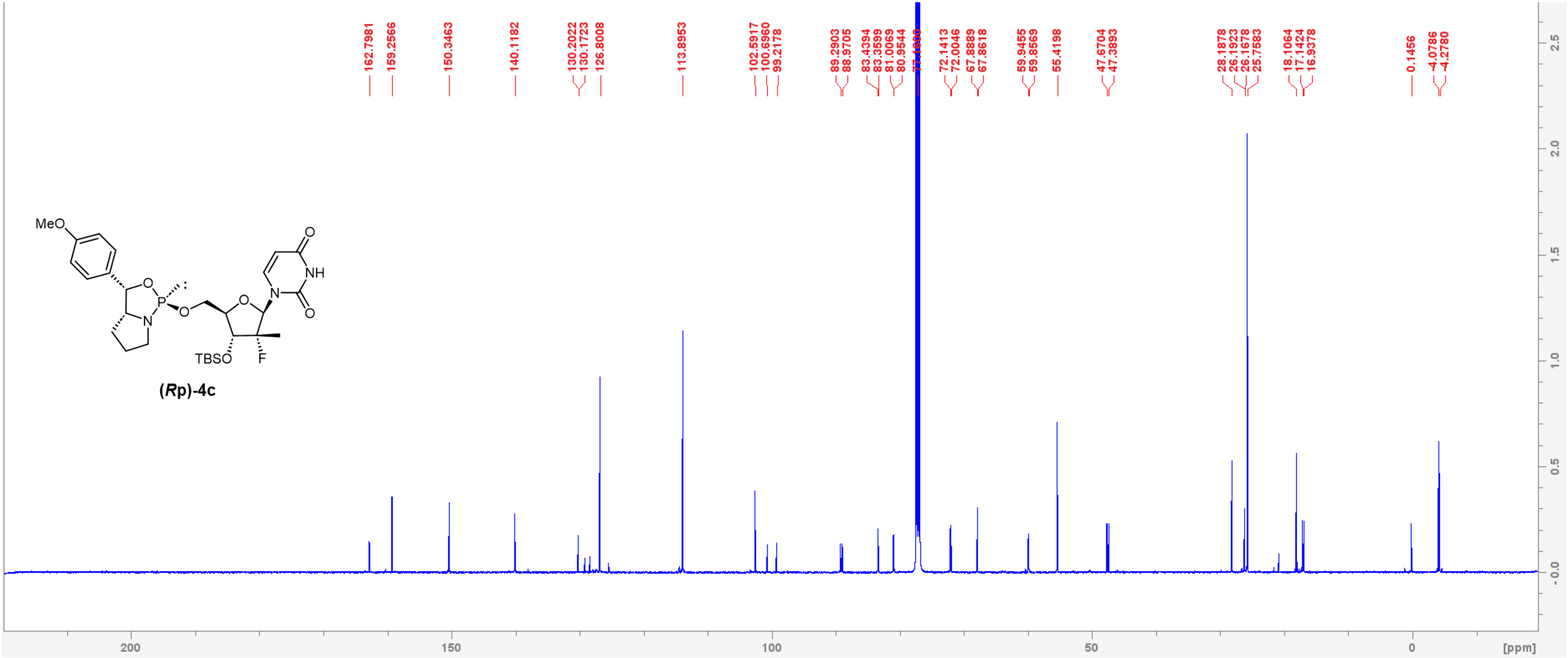

^{31}P {^{1}H} NMR (202 MHz, CDCl$_3$) of 5'-*O*-oxazaphospholidine derivative: (*R*p)-4c

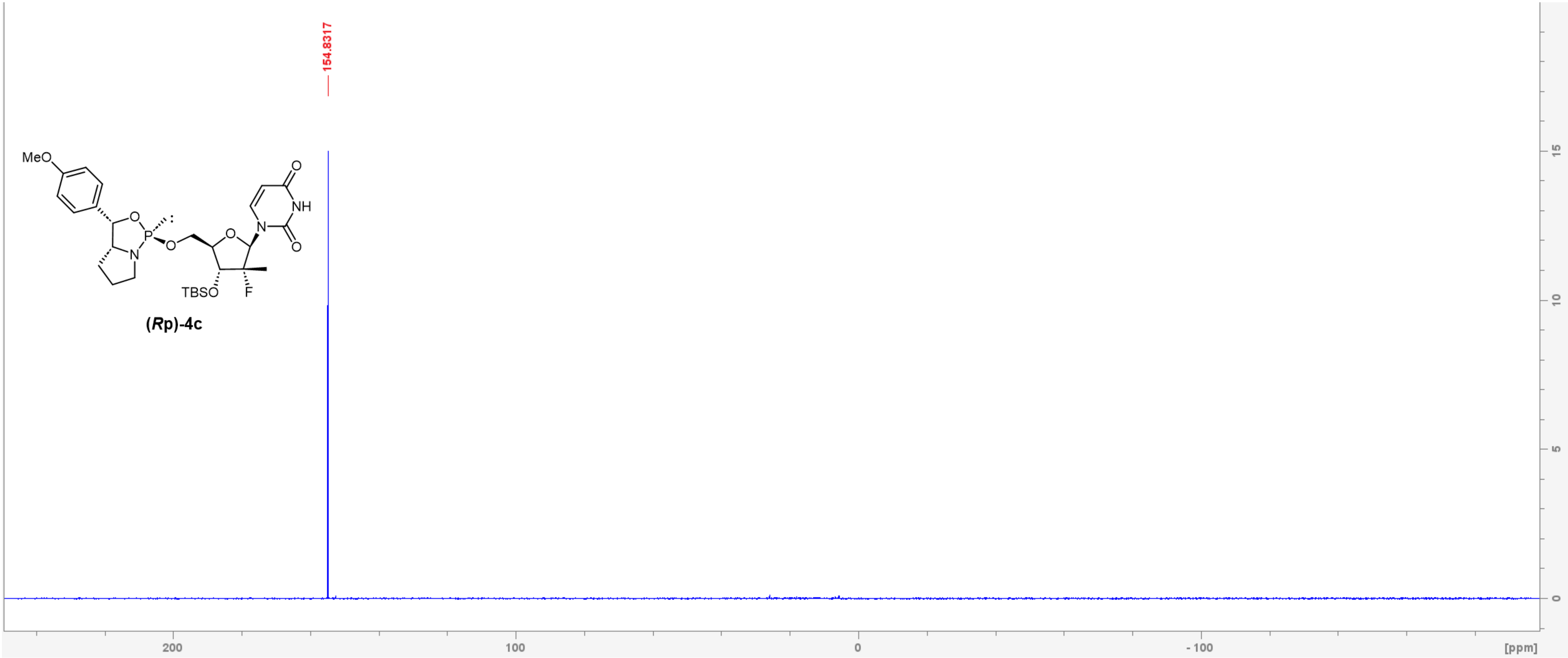

^{1}H NMR (500 MHz, $CDCl_3$) of 5'-*O*-oxazaphospholidine derivative: (*S*p)-4d

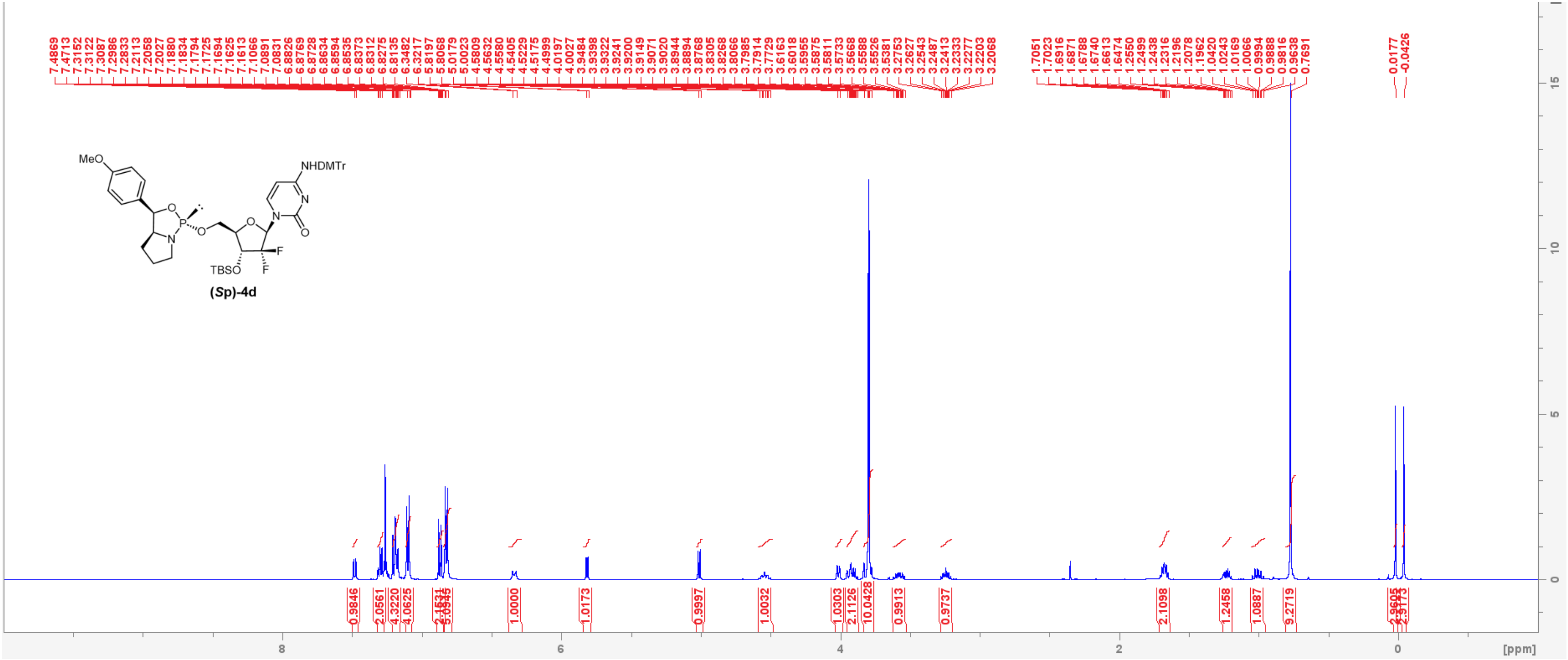

$^{13}C\{^{1}H\}$ NMR (101 MHz, $CDCl_3$) of 5'-*O*-oxazaphospholidine derivative: (*S*p)-4d

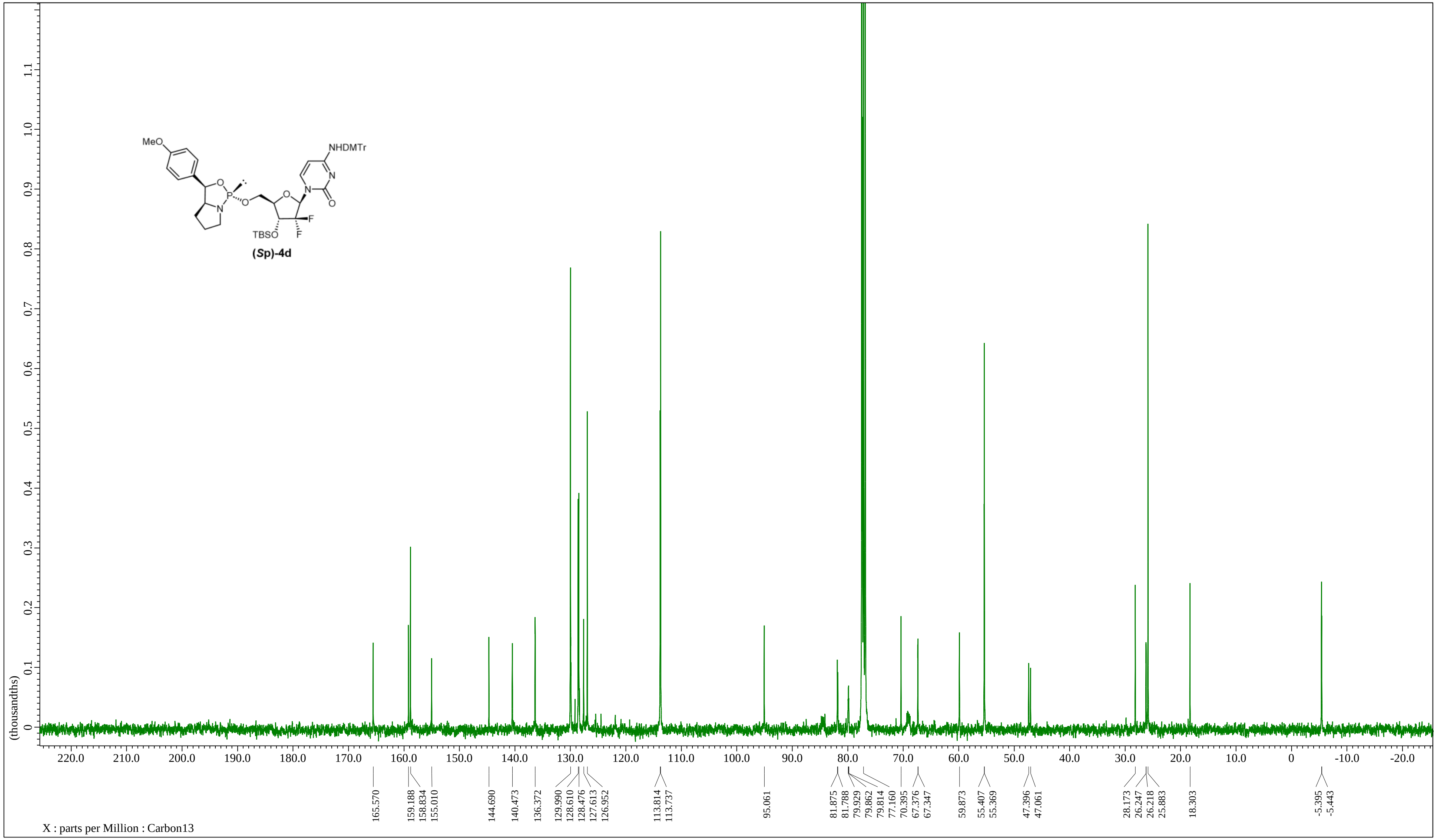

^{31}P {^{1}H} NMR (202 MHz, CDCl$_3$) of 5'-*O*-oxazaphospholidine derivative: (*S*p)-4d

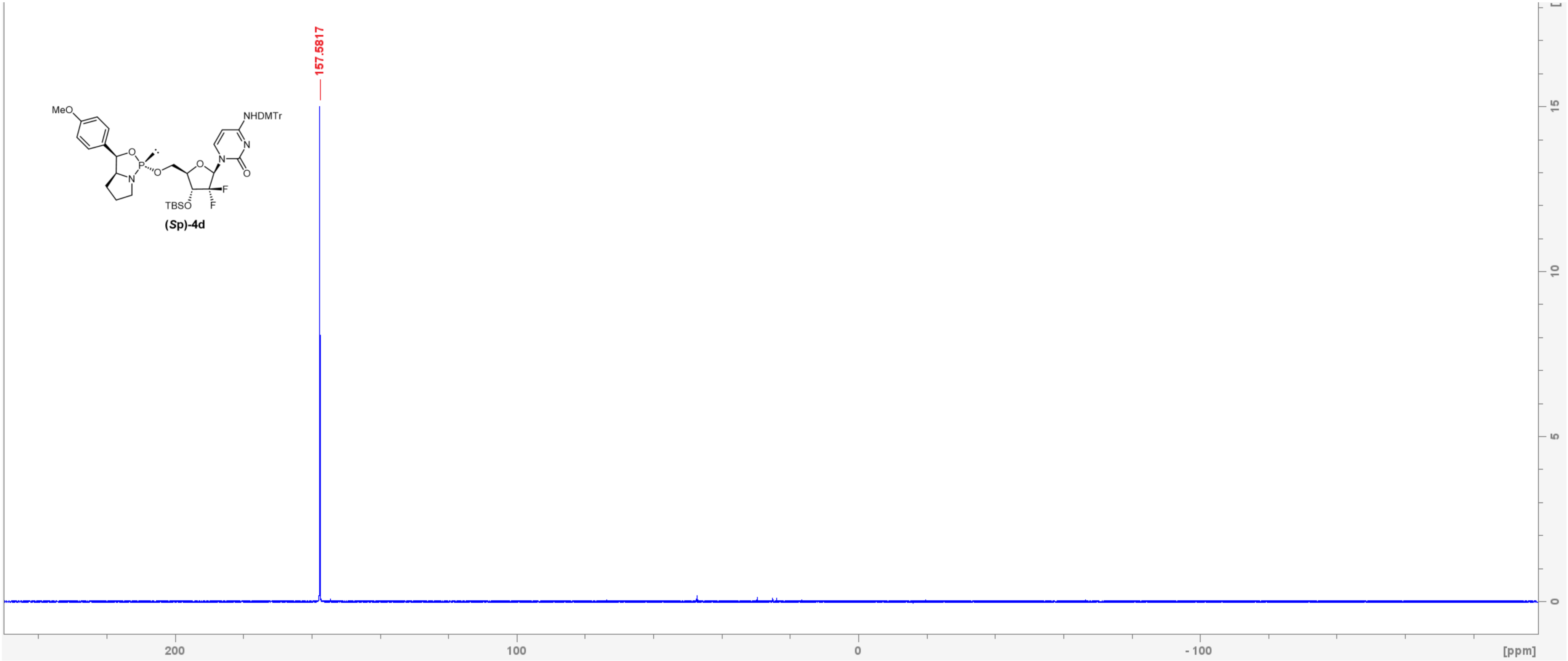

^{1}H NMR (500 MHz, CDCl$_3$) of (*R*p)-model phosphoramidate: 13a

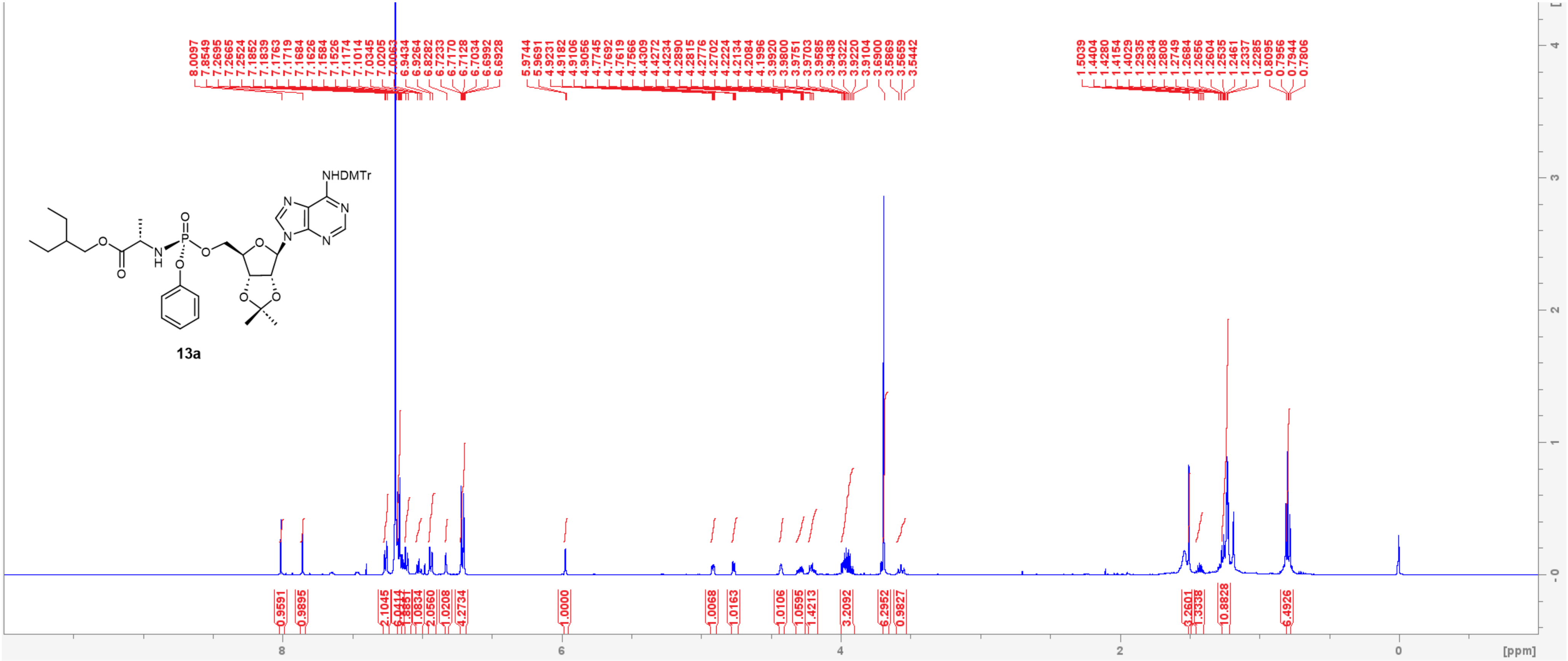

^{13}C {^{1}H} NMR (126 MHz, CDCl$_3$) of (*R*p)-model phosphoramidate: 13a

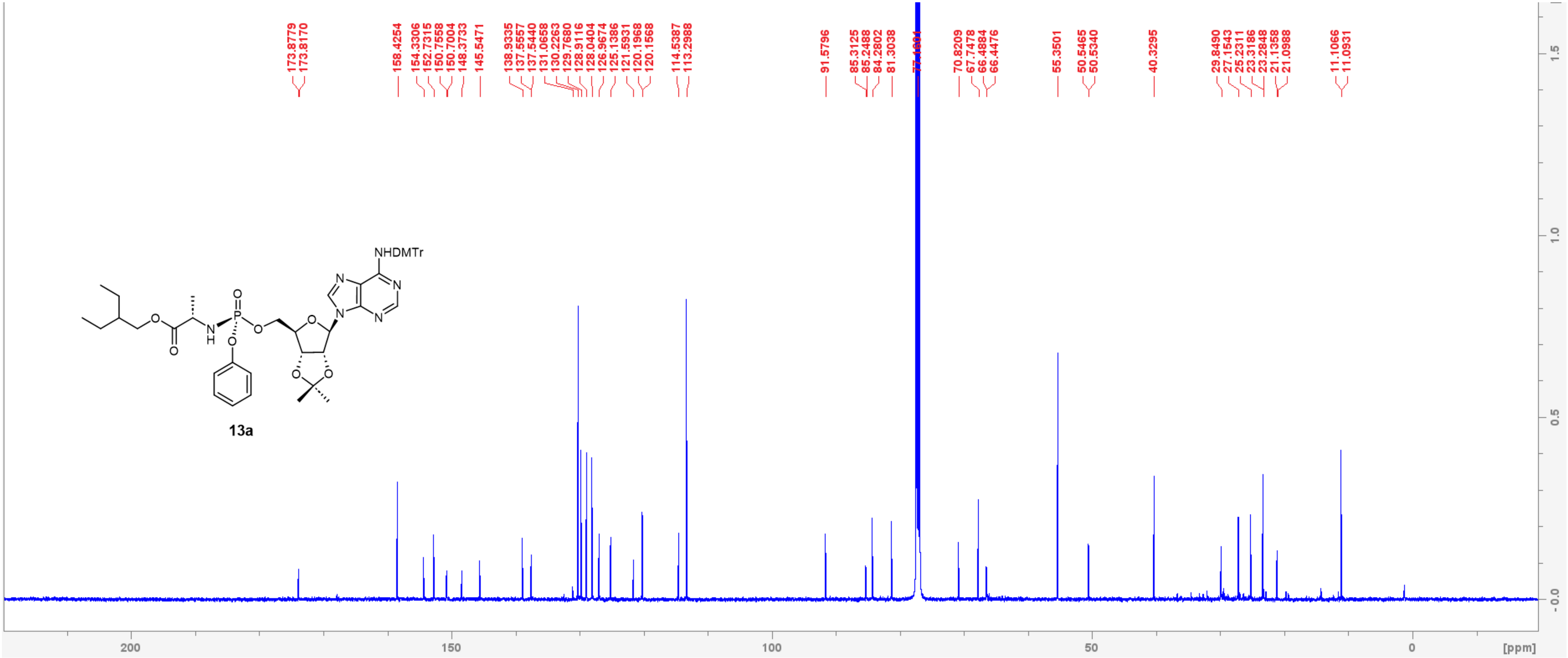

^{31}P {^{1}H} NMR (202 MHz, $CDCl_3$) of (*R*p)-model phosphoramidate: 13a

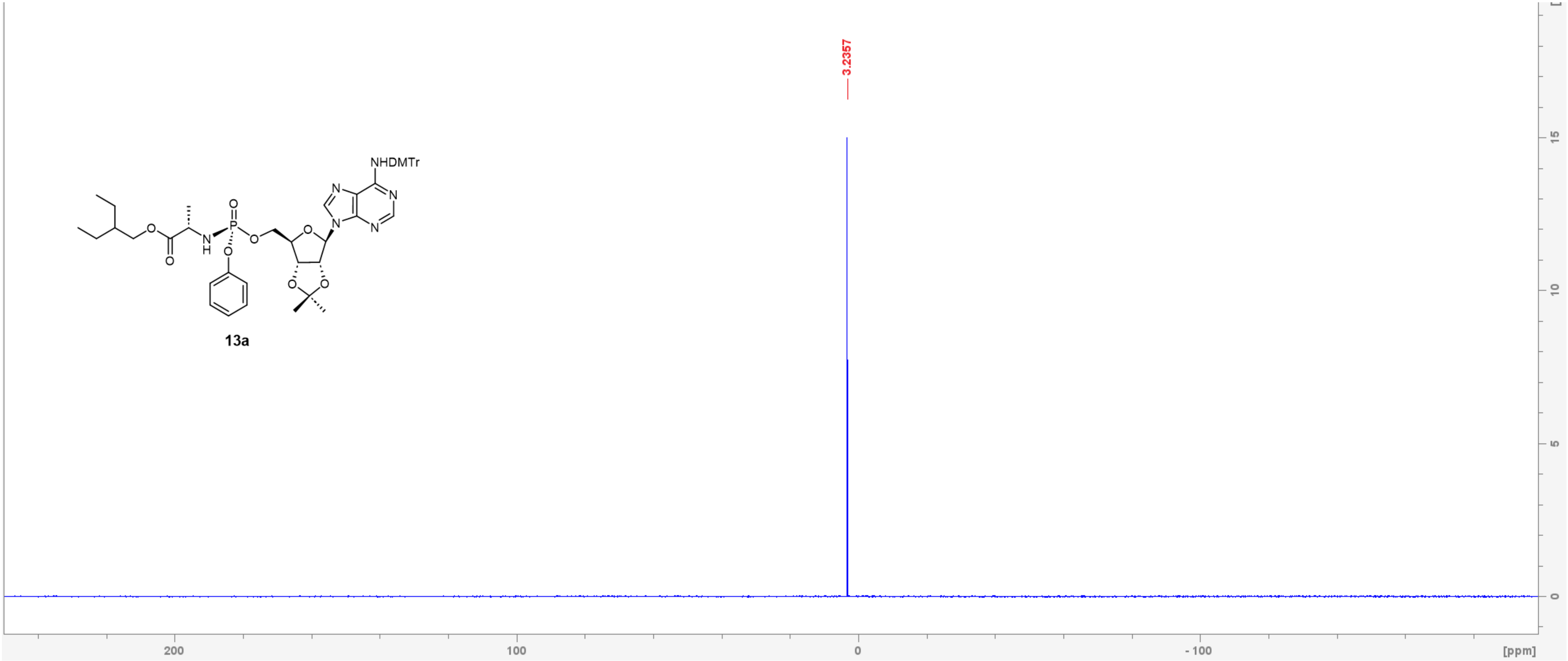

^{1}H NMR (500 MHz, CDCl3) of (*S*p)-model phosphoramidate: 13b

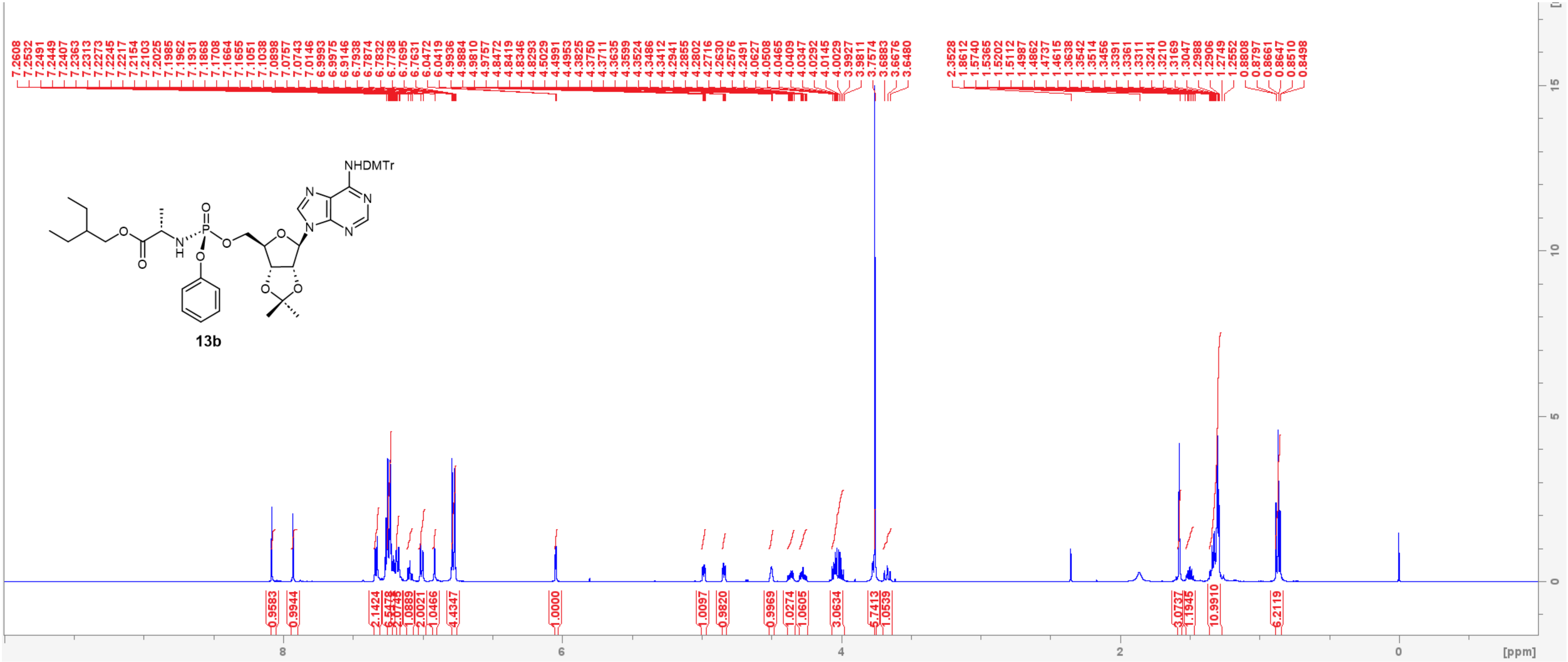

$^{13}C\{^1H\}$ NMR (126 MHz, $CDCl_3$) of (*S*p)-model phosphoramidate: 13b

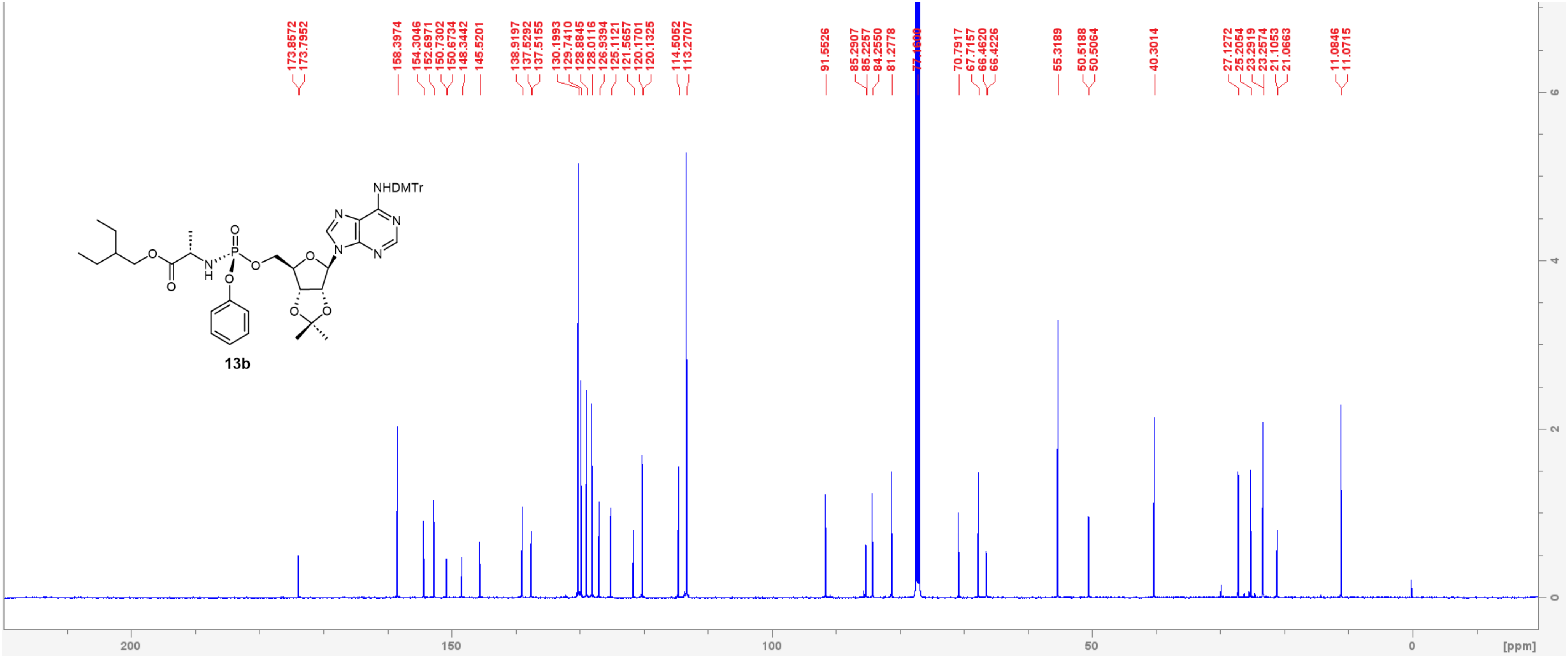

^{31}P {^{1}H} NMR (202 MHz, $CDCl_3$) of (*S*p)-model phosphoramidate 13b

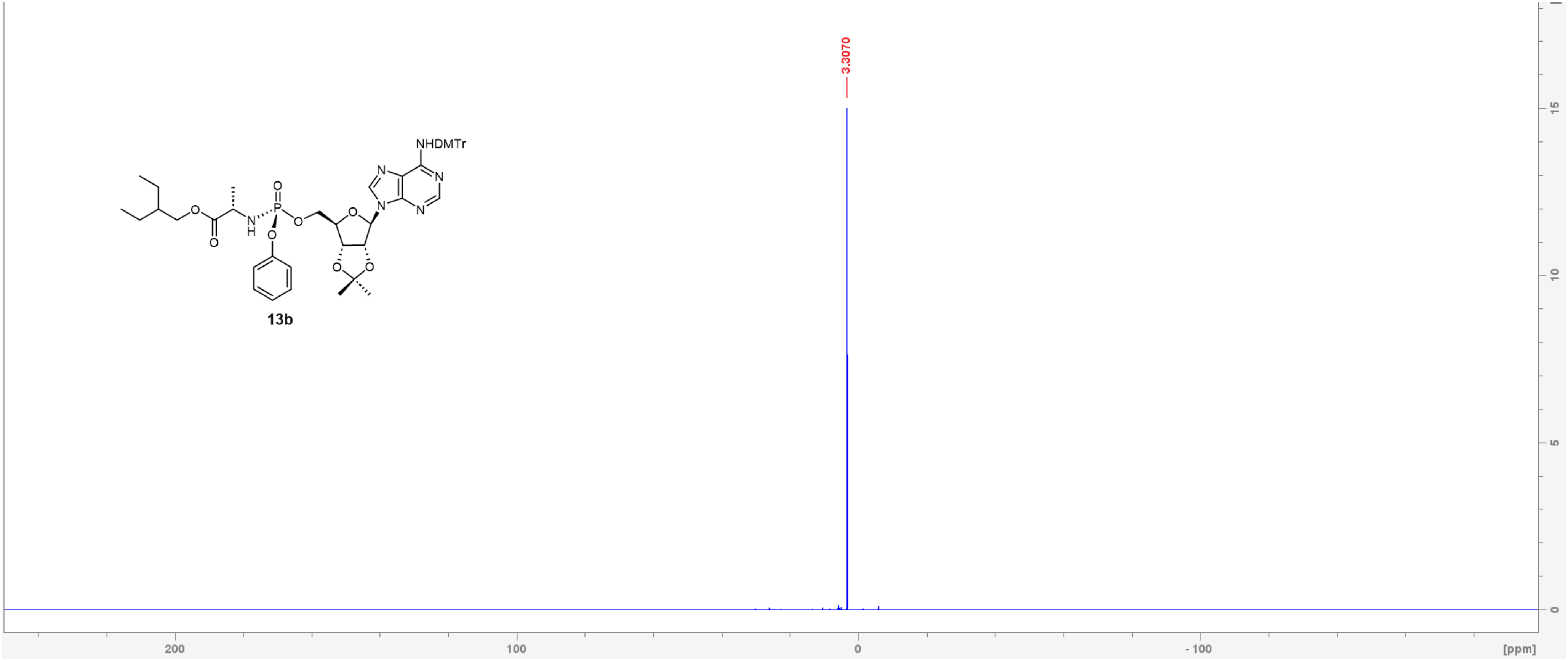

^{1}H NMR (500 MHz, DMSO-d6) of (*S*p)-Remdesivir: (*S*p)-1

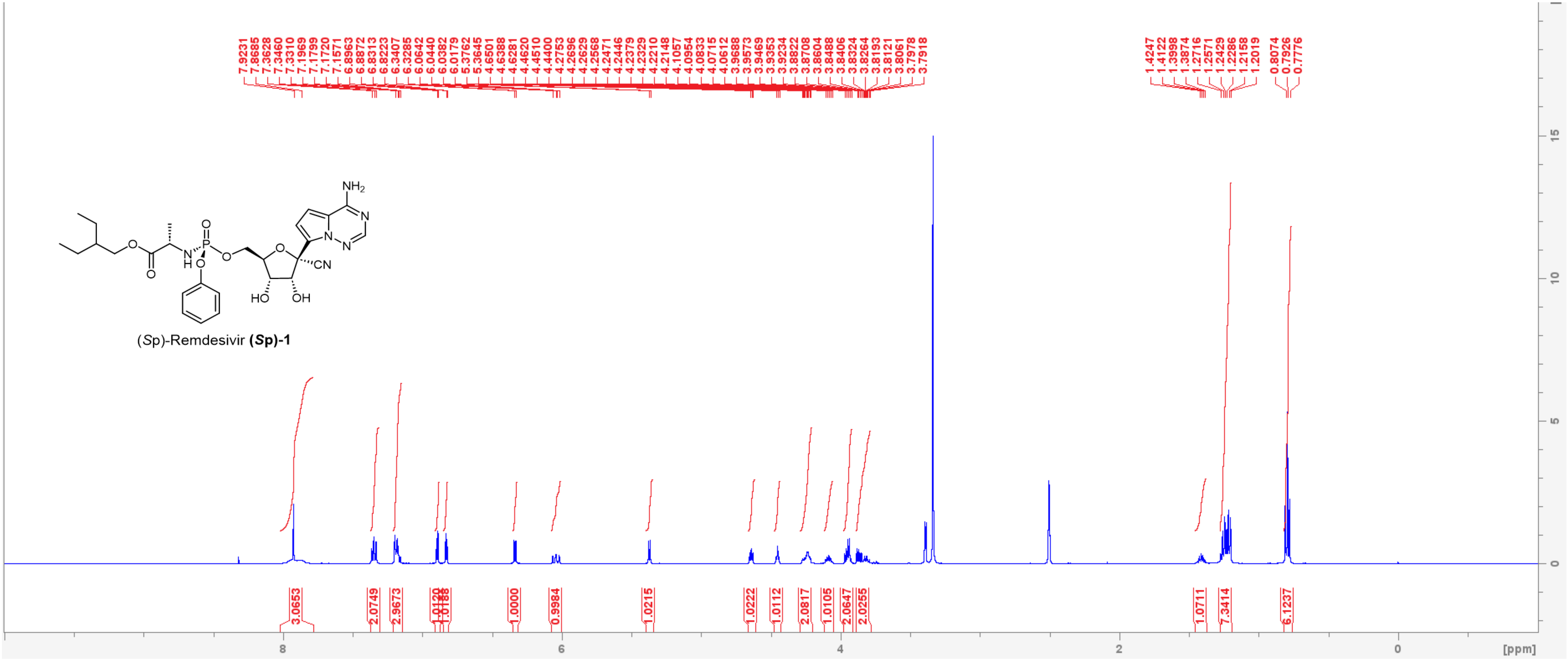

^{13}C {^{1}H} NMR (126 MHz, DMSO-d6) of (*S*p)-Remdesivir: (*S*p)-1

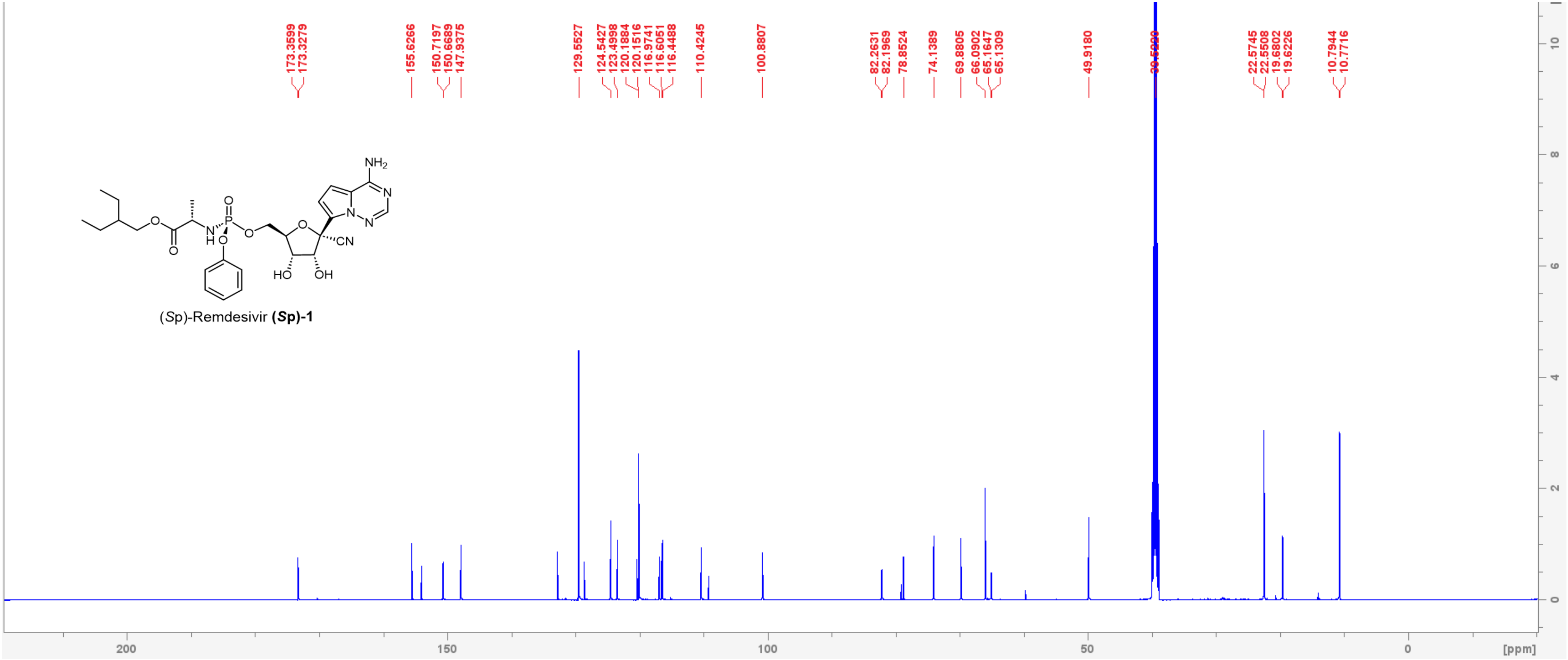

^{31}P {^{1}H} NMR (202 MHz, DMSO-d6) of (*S*p)-Remdesivir: (*S*p)-1

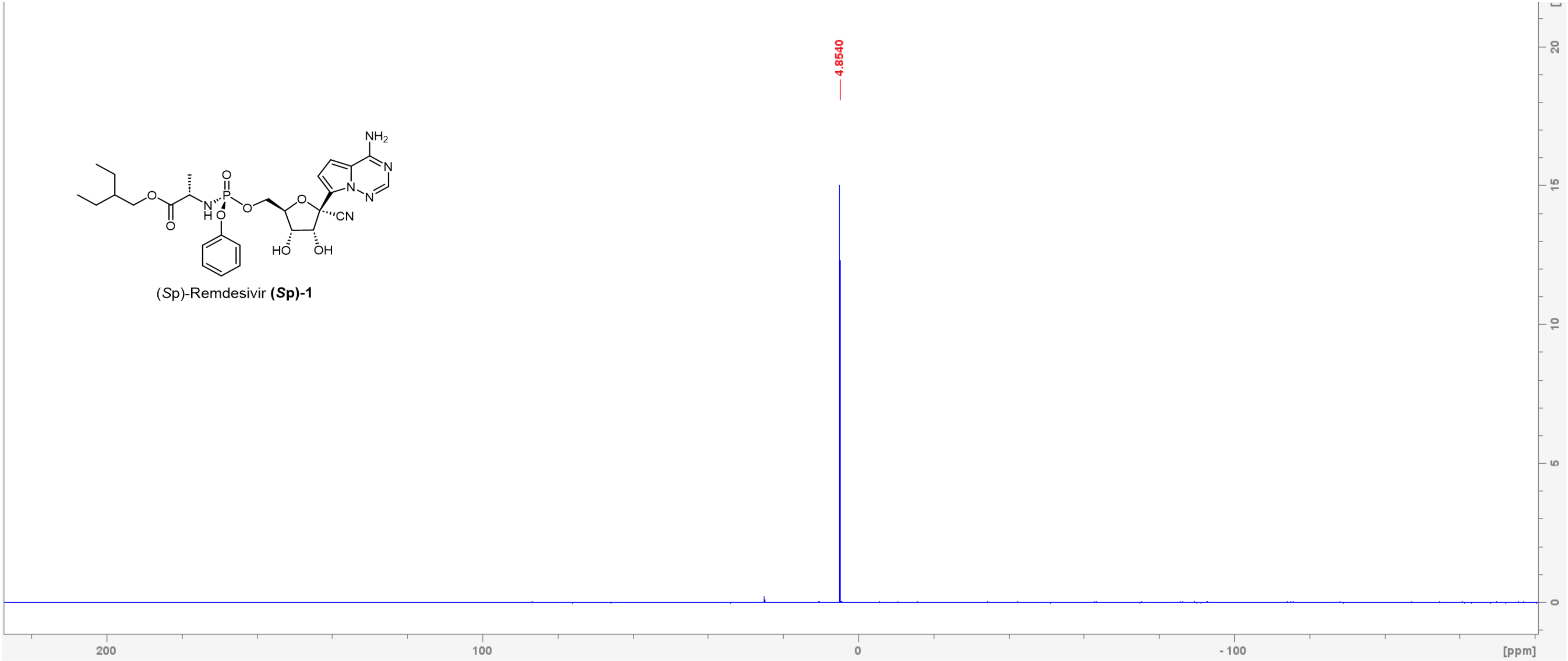

^{1}H NMR (500 MHz, DMSO-d6) of (*R*p)-Remdesivir: (*R*p)-1

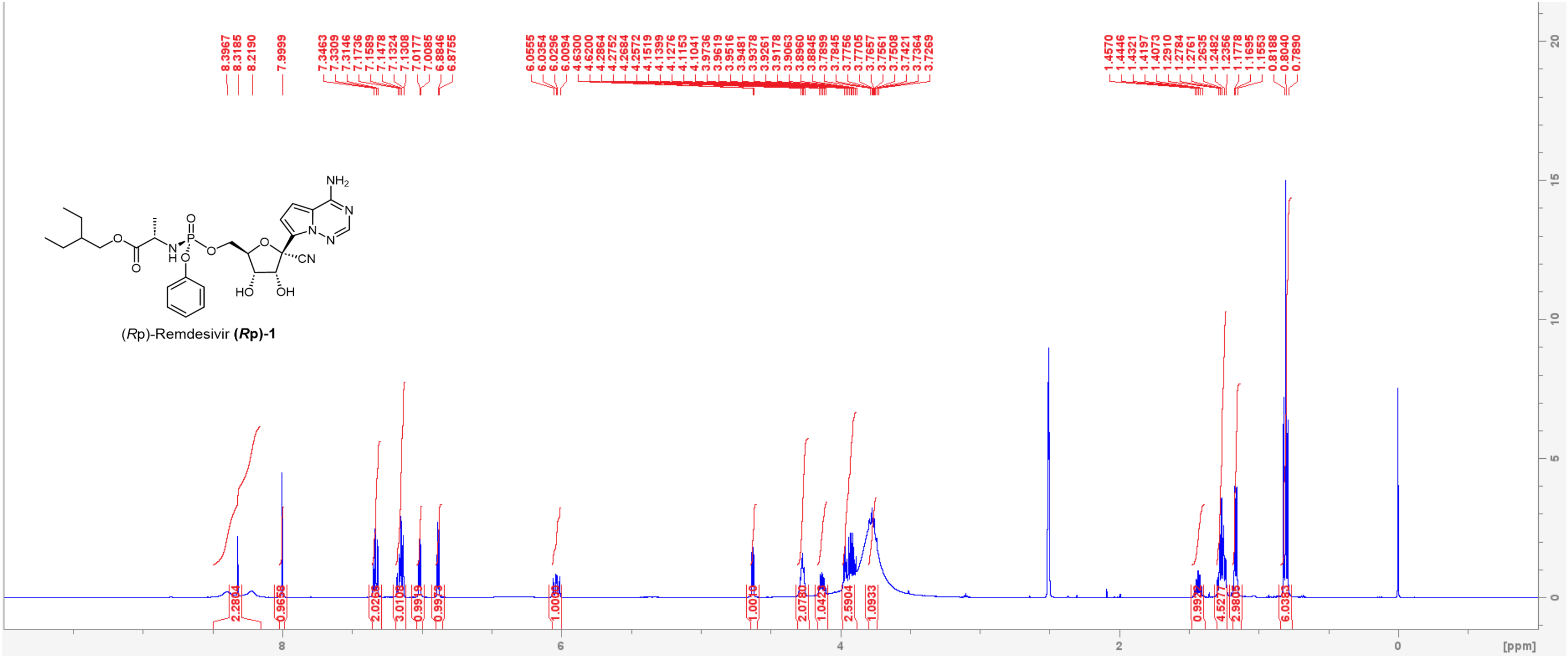

^{13}C {^{1}H} NMR (126 MHz, DMSO-d6) of (*R*p)-Remdesivir: (*R*p)-1

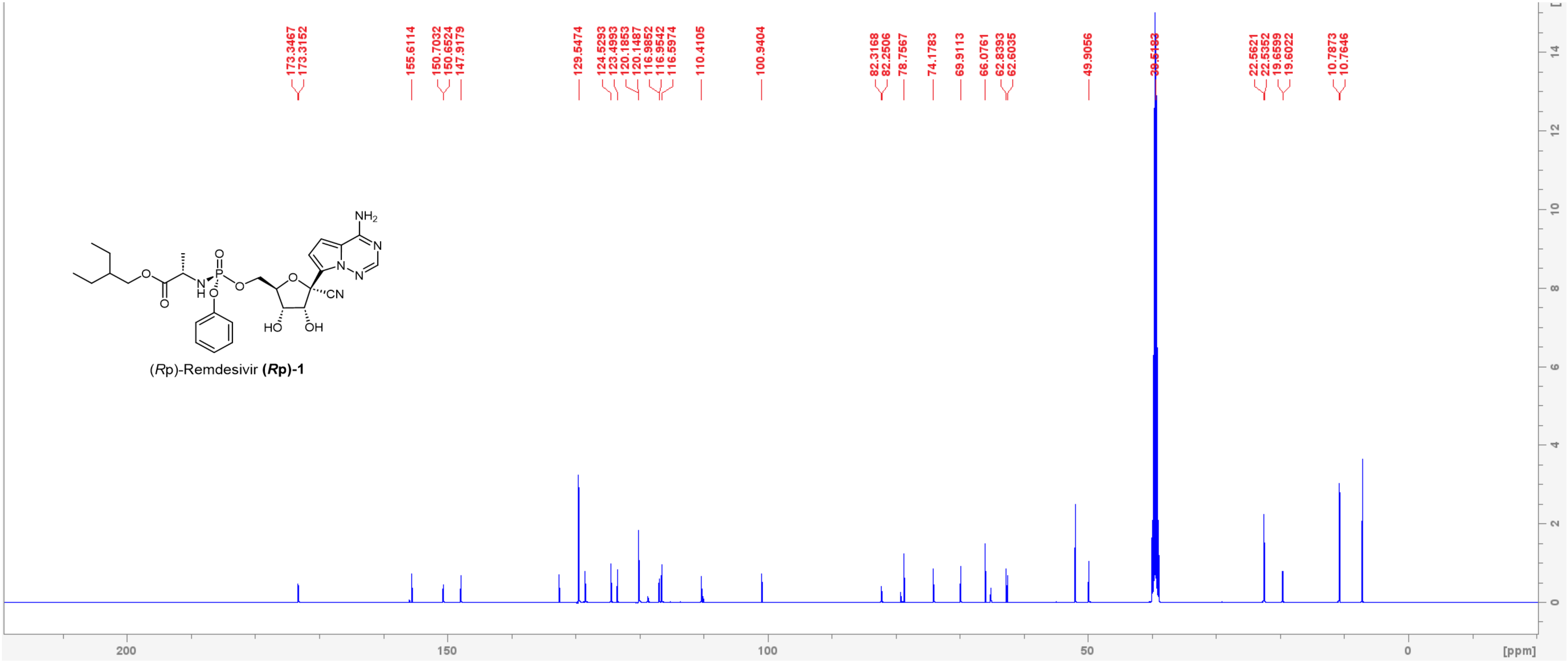

^{31}P {^{1}H} NMR (202 MHz, DMSO-d6) of (*R*p)-Remdesivir: (*R*p)-1

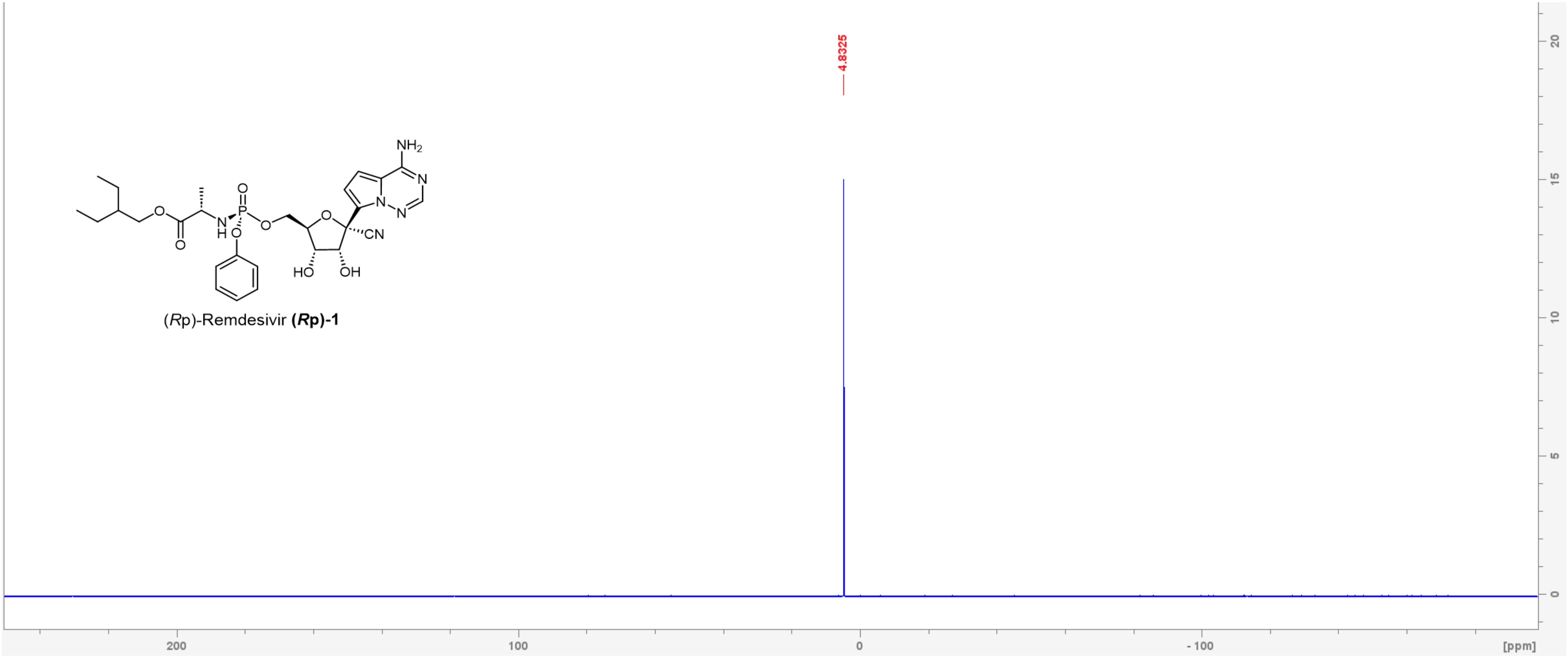

^{1}H NMR (500 MHz, DMSO-d6) of Sofosbuvir: (*S*p)-2

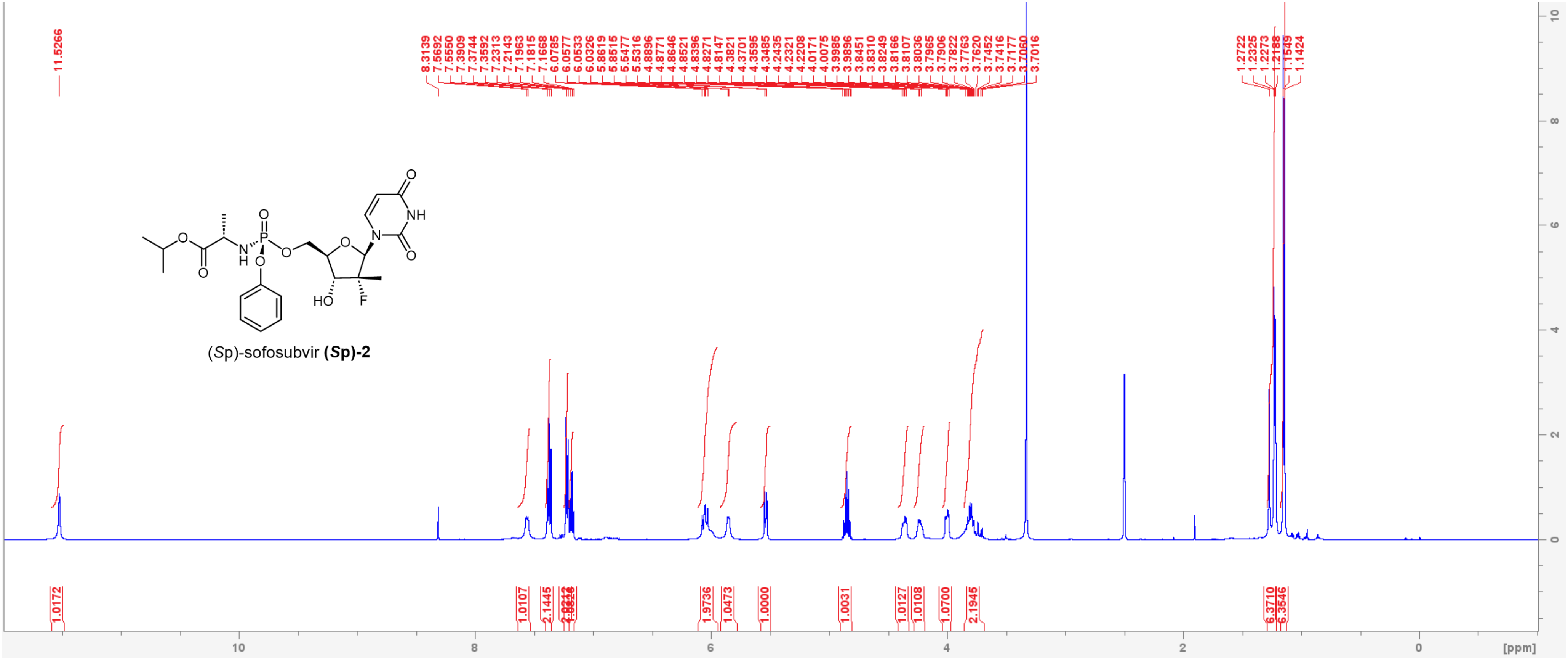

^{13}C {^{1}H} NMR (126 MHz, DMSO-d6) of Sofosbuvir: (*S*p)-2

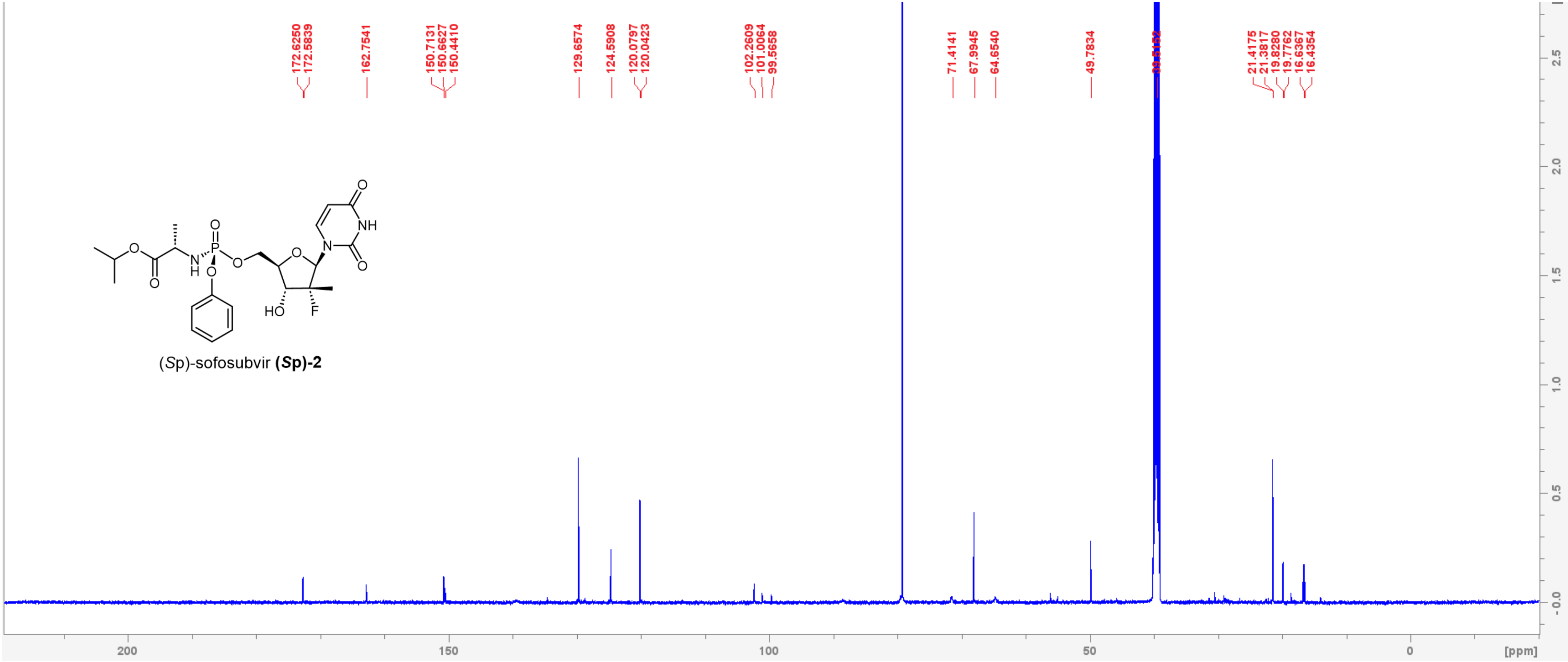

^{31}P {^{1}H} NMR (202 MHz, DMSO-d6) of Sofosbuvir: (*S*p)-2

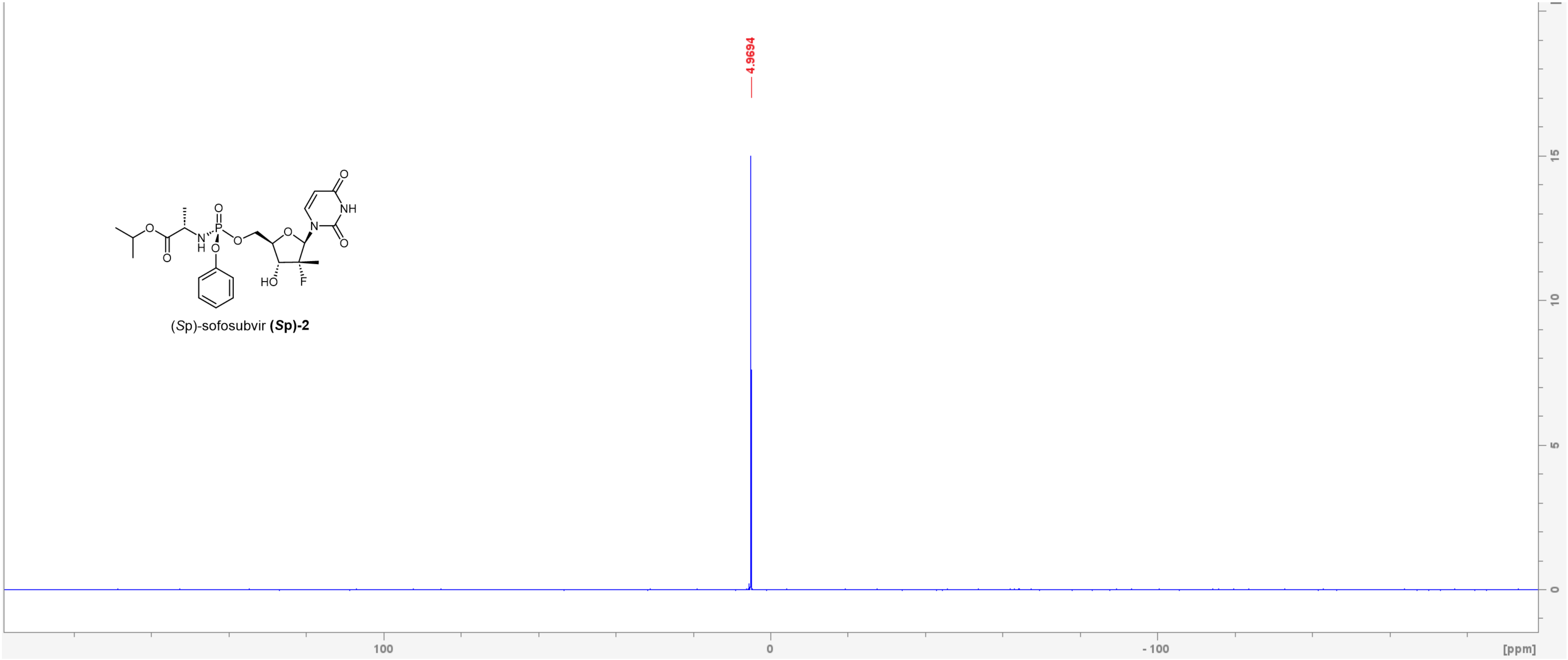

^{1}H NMR (500 MHz, DMSO-d6) of PSI-7976: (*R*p)-2

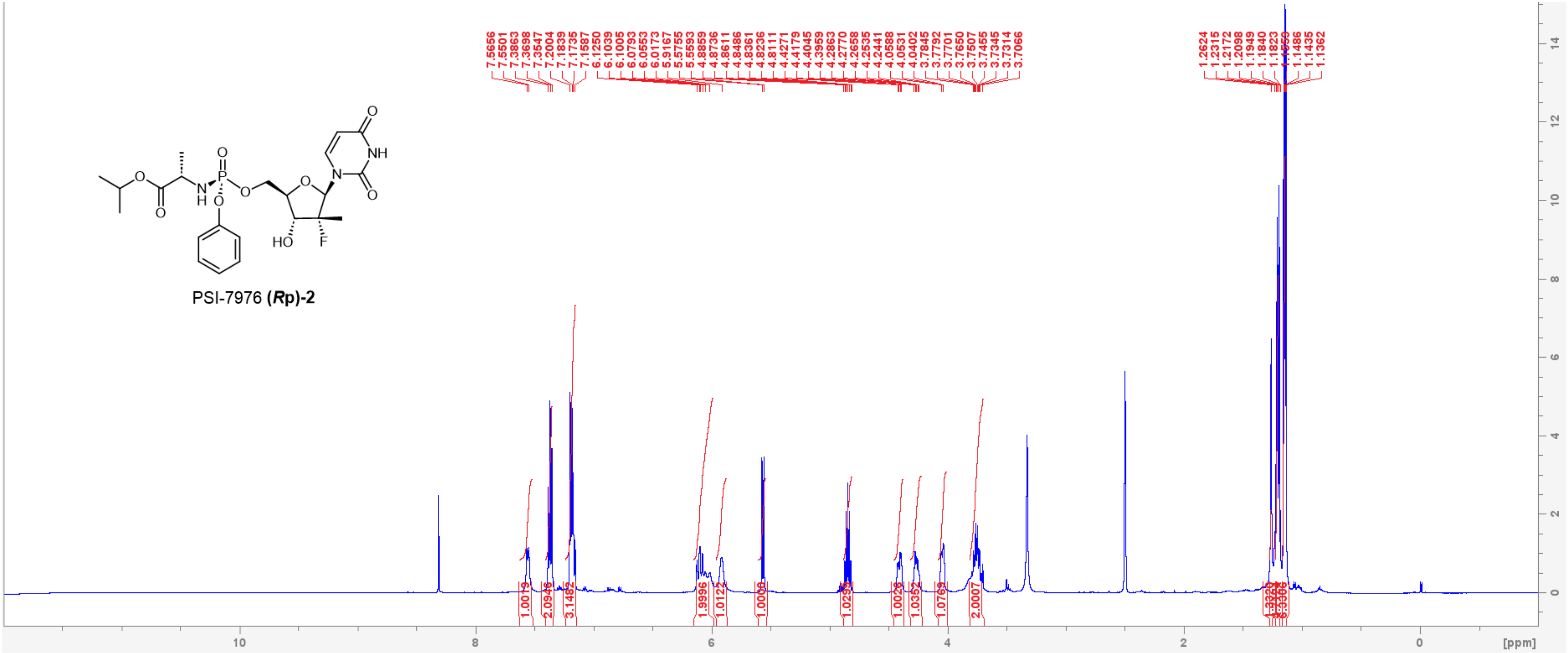

$^{13}C\{^{1}H\}$ **NMR (126 MHz, DMSO-d6) of PSI-7976: (*R*p)-2**

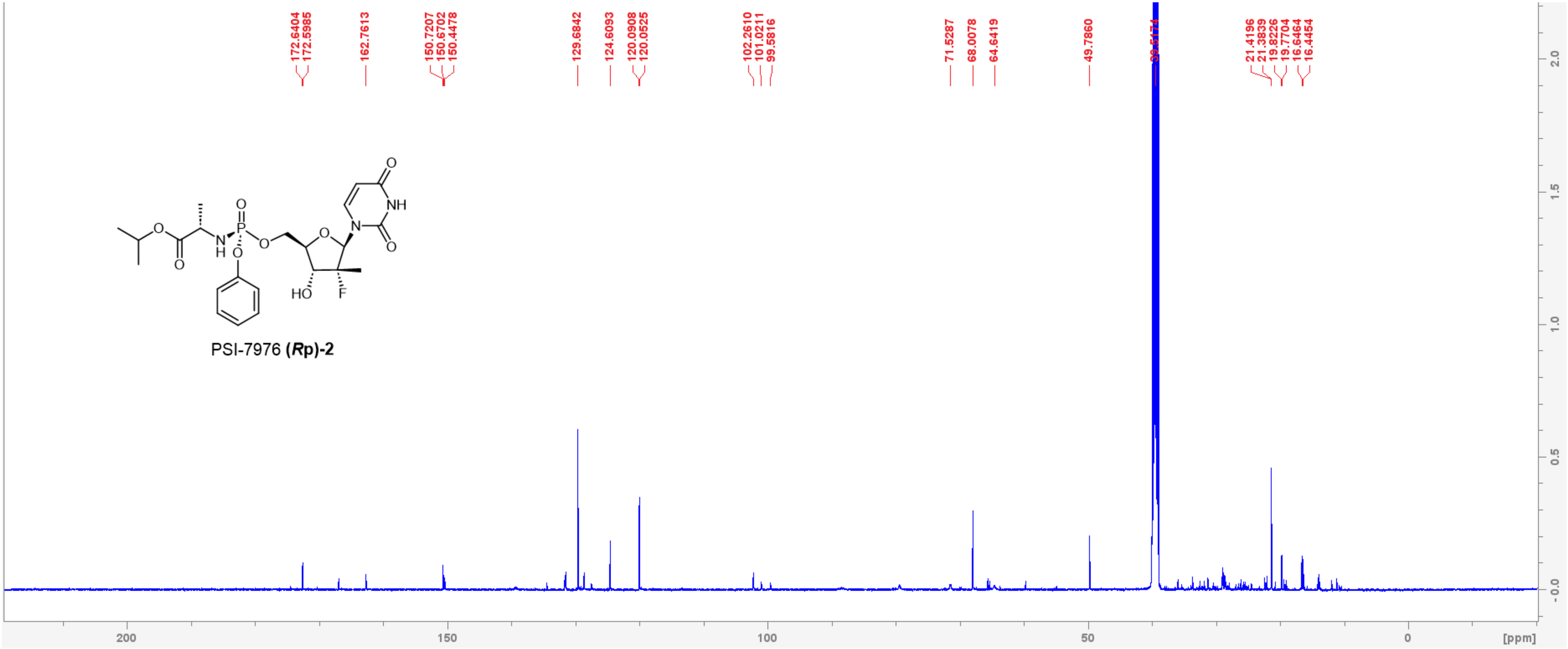

$^{31}P\{^1H\}$ NMR (202 MHz, DMSO-d6) of PSI-7976: (*R*p)-2

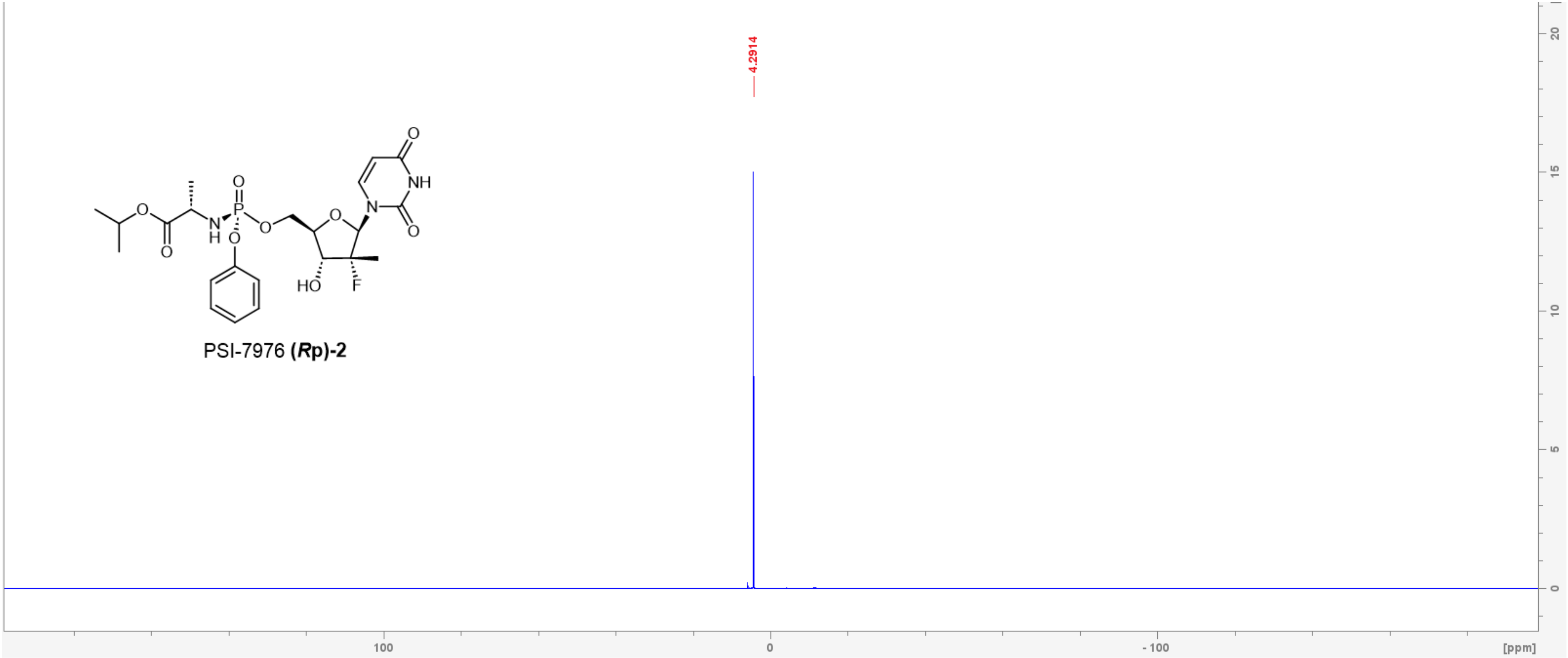

^{1}H NMR (500 MHz, CD_3OD) of NUC-1031: 3

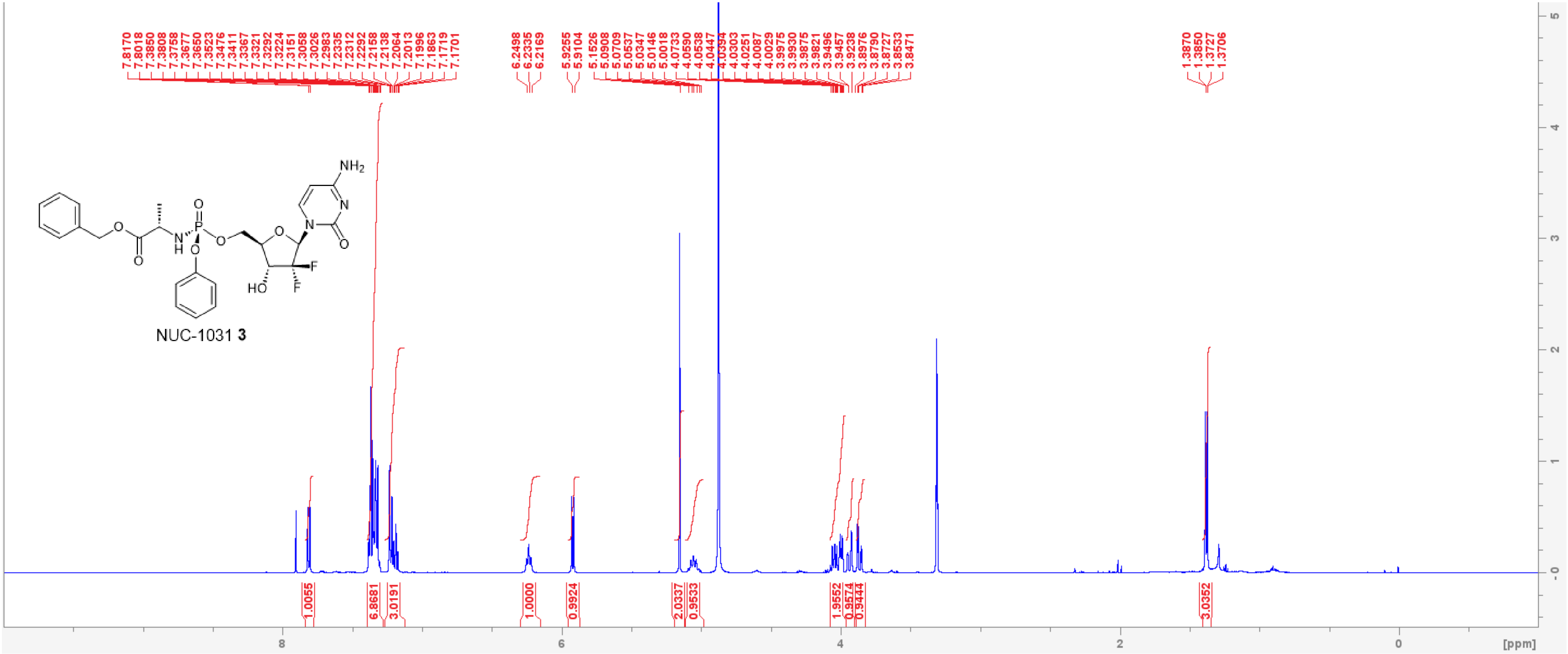

$^{13}C\ \{^{1}H\}$ NMR (126 MHz, CD_3OD) of 3

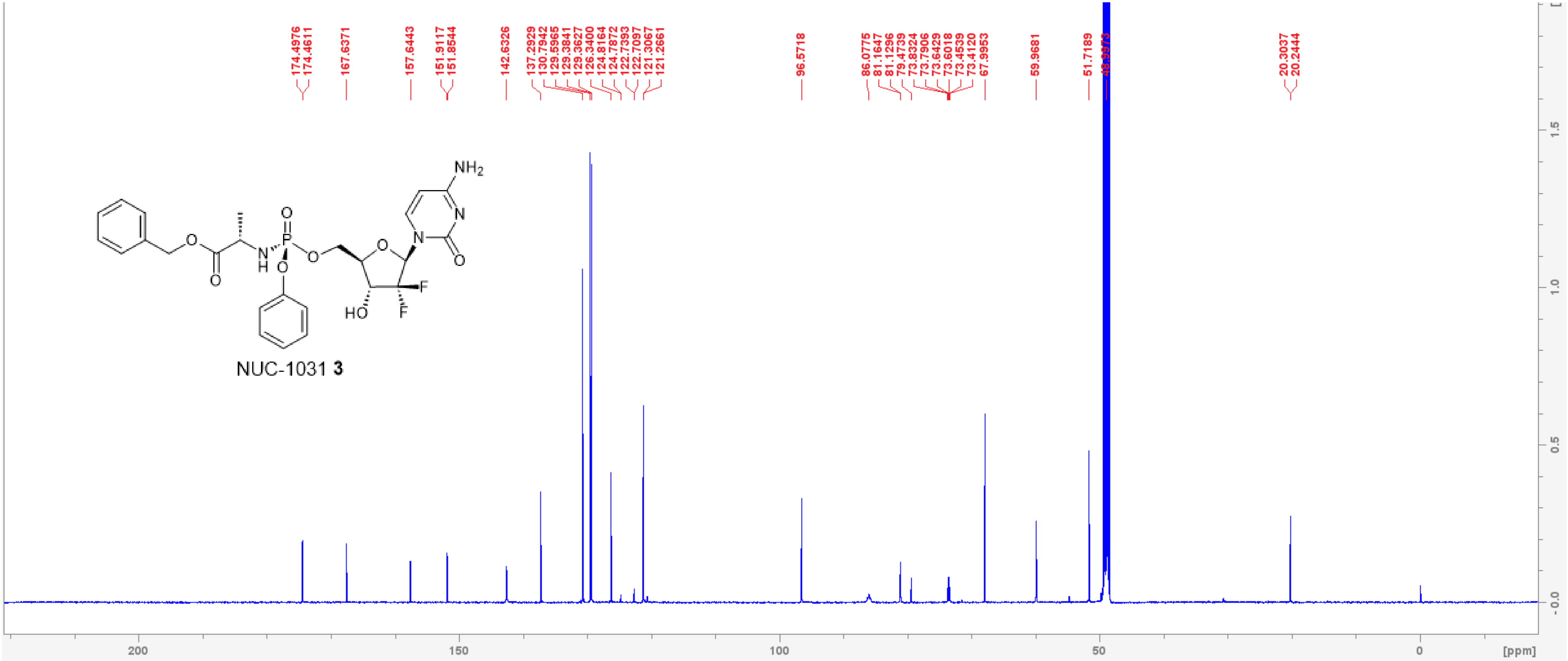

$^{31}P\{^{1}H\}$ NMR (202 MHz, CD_3OD) of NUC-1031: 3

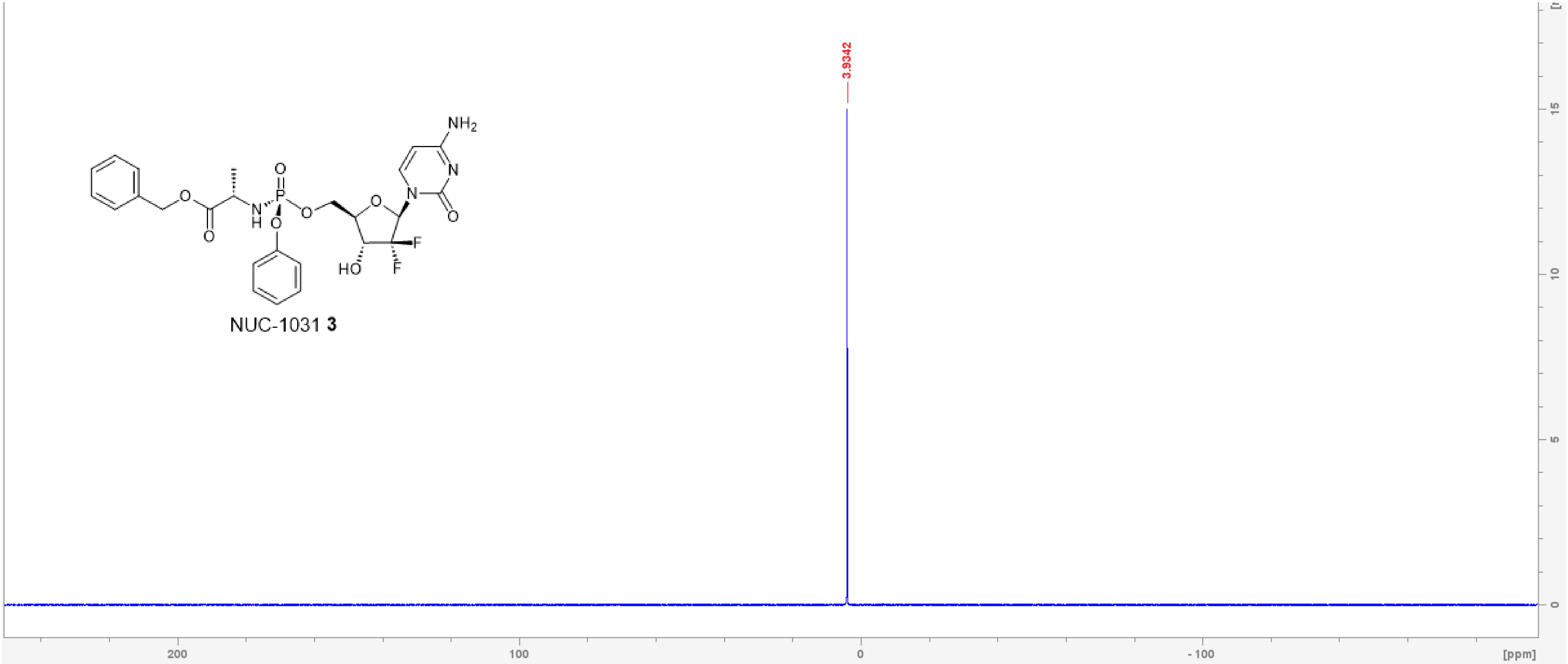

5. Reference

(1) Siegel, D.; Hui, H. C.; Doerffler, E.; Clarke, M. O.; Chun, K.; Zhang, L.; Neville, S.; Carra, E.; Lew, W.; Ross, B.; Wang, Q.; Wolfe, L.; Jordan, R.; Soloveva, V.; Knox, J.; Perry, J.; Perron, M.; Stray, K. M.; Barauskas, O.; Feng, J. Y.; Xu, Y.; Lee, G.; Rheingold, A. L.; Ray, A. S.; Bannister, R.; Strickley, R.; Swaminathan, S.; Lee, W. A.; Bavari, S.; Cihlar, T.; Lo, M. K.; Warren, T. K.; Mackman, R. L. Discovery and Synthesis of a Phosphoramidate Prodrug of a Pyrrolo[2,1-f][Triazin-4-Amino] Adenine C-Nucleoside (GS-5734) for the Treatment of Ebola and Emerging Viruses. *J. Med. Chem.* **2017**, *60* (5), 1648–1661. https://doi.org/10.1021/acs.jmedchem.6b01594.

(2) Slusarczyk, M.; Serpi, M.; Ghazaly, E.; Kariuki, B. M.; McGuigan, C.; Pepper, C. Single Diastereomers of the Clinical Anticancer ProTide Agents NUC-1031 and NUC-3373 Preferentially Target Cancer Stem CellsIn Vitro. *J. Med. Chem.* **2021**, *64* (12), 8179–8193. https://doi.org/10.1021/acs.jmedchem.0c02194.

(3) Hara, R. I.; Saito, T.; Kogure, T.; Hamamura, Y.; Uchiyama, N.; Nukaga, Y.; Iwamoto, N.; Wada, T. Stereocontrolled Synthesis of Boranophosphate DNA by an Oxazaphospholidine Approach and Evaluation of Its Properties. *J. Org. Chem.* **2019**, *84* (12), 7971–7983. https://doi.org/10.1021/acs.joc.9b00658.

(4) Moriguchi, T.; Asai, N.; Okada, K.; Seio, K.; Sasaki, T.; Sekine, M. First Synthesis and Anticancer Activity of Phosmidosine and Its Related Compounds. *J. Org. Chem.* **2002**, *67* (10), 3290–3300. https://doi.org/10.1021/jo016176g.

(5) Pradere, U.; Amblard, F.; Coats, S. J.; Schinazi, R. F. Synthesis of 5′-Methylene-Phosphonate Furanonucleoside Prodrugs: Application to D-2′-Deoxy-2′-α-Fluoro-2′-β-C-Methyl Nucleosides. *Org. Lett.* **2012**, *14* (17), 4426–4429. https://doi.org/10.1021/ol301937v.
